# A draft genome sequence and functional screen reveals the repertoire of type III secreted proteins of *Pseudomonas syringae *pathovar *tabaci *11528

**DOI:** 10.1186/1471-2164-10-395

**Published:** 2009-08-24

**Authors:** David J Studholme, Selena Gimenez Ibanez, Daniel MacLean, Jeffery L Dangl, Jeff H Chang, John P Rathjen

**Affiliations:** 1 The Sainsbury Laboratory, Norwich, NR4 7UH, UK; 2 Department of Biology, CB# 3280, Coker Hall, The University of North Carolina at Chapel Hill, Chapel Hill, North Carolina 27599-3280, USA; 3 Department of Botany and Plant Pathology, Oregon State University, 2082 Cordley Hall, Corvallis, OR 97331, USA; 4 Center for Genome Research and Biocomputing, Oregon State University, 2082 Cordley Hall, Corvallis, OR 97331, USA

## Abstract

**Background:**

*Pseudomonas syringae *is a widespread bacterial pathogen that causes disease on a broad range of economically important plant species. Pathogenicity of *P. syringae *strains is dependent on the type III secretion system, which secretes a suite of up to about thirty virulence 'effector' proteins into the host cytoplasm where they subvert the eukaryotic cell physiology and disrupt host defences. *P. syringae *pathovar *tabaci *naturally causes disease on wild tobacco, the model member of the Solanaceae, a family that includes many crop species as well as on soybean.

**Results:**

We used the 'next-generation' Illumina sequencing platform and the Velvet short-read assembly program to generate a 145X deep 6,077,921 nucleotide draft genome sequence for *P. syringae *pathovar *tabaci *strain 11528. From our draft assembly, we predicted 5,300 potential genes encoding proteins of at least 100 amino acids long, of which 303 (5.72%) had no significant sequence similarity to those encoded by the three previously fully sequenced *P. syringae *genomes. Of the core set of Hrp Outer Proteins that are conserved in three previously fully sequenced *P. syringae *strains, most were also conserved in strain 11528, including AvrE1, HopAH2, HopAJ2, HopAK1, HopAN1, HopI, HopJ1, HopX1, HrpK1 and HrpW1. However, the *hrpZ1 *gene is partially deleted and *hopAF1 *is completely absent in 11528. The draft genome of strain 11528 also encodes close homologues of HopO1, HopT1, HopAH1, HopR1, HopV1, HopAG1, HopAS1, HopAE1, HopAR1, HopF1, and HopW1 and a degenerate HopM1'. Using a functional screen, we confirmed that *hopO1, hopT1, hopAH1*, *hopM1'*, *hopAE1*, *hopAR1*, and *hopAI1' *are part of the virulence-associated HrpL regulon, though the *hopAI1' *and *hopM1' *sequences were degenerate with premature stop codons. We also discovered two additional HrpL-regulated effector candidates and an HrpL-regulated distant homologue of *avrPto1*.

**Conclusion:**

The draft genome sequence facilitates the continued development of *P. syringae *pathovar *tabaci *on wild tobacco as an attractive model system for studying bacterial disease on plants. The catalogue of effectors sheds further light on the evolution of pathogenicity and host-specificity as well as providing a set of molecular tools for the study of plant defence mechanisms. We also discovered several large genomic regions in *Pta *11528 that do not share detectable nucleotide sequence similarity with previously sequenced *Pseudomonas *genomes. These regions may include horizontally acquired islands that possibly contribute to pathogenicity or epiphytic fitness of *Pta *11528.

## Background

*Pseudomonas syringae *is a widespread bacterial pathogen that causes disease on a broad range of economically important plant species. The species *P. syringae *is sub-divided into about 50 pathovars, each exhibiting characteristic disease symptoms and distinct host-specificities. *P. syringae *pathovar *tabaci *(*Pta*) causes wild-fire disease in soybean and tobacco plants [1,2], characterised by chlorotic halos surrounding necrotic spots on the leaves of infected plants. Formation of halos is dependent on the beta-lactam tabtoxin, which causes ammonia accumulation in the host cell by inhibition of glutamine synthetase [3]. However, whether tabtoxin is an essential component of the disease process is unclear [4,5].

Pathogenicity of *P. syringae *strains is dependent on the type III secretion system (T3SS). The T3SS secretes a suite of virulence 'effector' proteins into the host cytoplasm where they subvert the eukaryotic cell physiology and disrupt host defences [6-14]. Mutants lacking the T3SS do not secrete effectors, and as a consequence do not infect plants or induce disease symptoms. Thus, understanding effector action is central to understanding bacterial pathogenesis. A single *P. syringae *strain typically encodes about 30 different effectors [14]. However, different *P. syringae *strains have different complements of effector genes. The emerging view is that of a core of common effectors encoded by most strains, augmented by a variable set. Individual effectors appear to act redundantly with each other and are individually dispensable with a small or no loss to pathogen virulence [10]. Effectors are also thought to play an important role in determining host range. This is most clearly true when infections are restricted by host defences. Some plants have evolved specific mechanisms to recognise certain effectors; such recognition induces strong host defences which curtail infection. For example, expression of the T3SS effector HopQ1-1 from *P. syringae *pathovar *tomato *(*Pto*) DC3000 was sufficient to render *Pta *11528 avirulent on *Nicotiana benthamiana *[15]. The opposite situation, in which acquisition of a novel effector gene confers the ability to infect new host plants, has not been demonstrated and remains speculative. However, heterologous expression of the effector gene *avrPtoB *conferred a plasmid-cured strain of *P. syringae *pathovar *phaseolicola *(*Pph*) with increased virulence [16]. We hope that further identification and characterisation of effector repertoires of particular strains will shine new light on their roles in determining host range. Finally, bacterial virulence is also likely to be influenced by other non-T3SS-dependent virulence factors such as toxins which are often co-regulated with the T3SS [17].

Complete genome sequences are available for strains representing three *P. syringae *pathovars: *Pto*, pathovar *phaseolicola *(*Pph*) and pathovar *syringae *(*Psy*) [18-20]. Comparisons of these have led to the identification of core effector gene sets and to explain some of the differences in host-specificity between pathovars. However, these three sequenced strains are representatives of three distinct phylogroups within the species *P. syringae*, and as such are phylogenetically quite distant [21,22]. According to DNA-DNA hybridisation studies and ribotyping [21], *P. syringae *can be divided into 9 discrete genomospecies. Representative strains of *Psy*, *Pph *and *Pto *fell into genomospecies one, two and three respectively [21]. Recently, a strain of pathovar *oryzae *(genomospecies four) was sequenced [23]. A draft genome sequence was also published for *Pto *T1 [24], a strain closely related to *Pto *DC3000 but restricted to tomato hosts, whereas *Pto *DC3000 is able to cause disease on *Arabidopsis*. In the current study, we explore genetic differences at an intermediate phylogenetic resolution; that is, we compared the genome sequences of *Pta *11528 to that of *P. phaseolicola *(*Pph*) 1448A, which resides within the same phylogroup but possesses a distinct host range and causes different disease symptoms.

*Pto *DC3000 was the first plant-pathogenic pseudomonad to have its genome sequenced, helping to establish the *Arabidopsis*-*Pto *system as the primary model for plant-microbe interactions. However, *Arabidopsis *is not a natural host of *Pto*, and it is important to develop alternative systems given the genetic variability of *P. syringae *strains, particularly in regard to effectors. We work on the interaction between *Pta *and the wild tobacco plant *N. benthamiana*, which offers certain advantages over *Arabidopsis*. Firstly, *N. benthamiana *is an important model for the Solanaceae, which includes many important crop species. The *Pta*-*N. benthamiana *interaction is a natural pathosystem. Lastly, *N. benthamiana *is an important model plant that is more amenable to biochemistry-based approaches and facile manipulation of gene expression such as virus-induced gene silencing (VIGS). Thus *N. benthamiana *provides experimental options for understanding plant-bacterial interactions. Strains of *Pta *can cause disease on *N. benthamiana*, but relatively few genetic sequence data are available for this pathovar.

In this study we generated a draft complete genome sequence of *Pta *11528 and used a functional screen for HrpL-dependent genes to infer its repertoire of T3SS effectors and associated Hrp Outer Proteins (Hops), which differs significantly from that of its closest relative whose complete genome has previously been published (*Pph *1448A). *Pta *11528 does not encode functional homologues of HopAF1 or HrpZ1. This was surprising since HopAF1 was conserved in the three previously sequenced pathovars [18-20]. HrpZ1 is conserved in most strains of *P. syringae *that have been investigated, albeit with differences in amino acid sequence [25]. However, Pta strain 6605 and several other isolates from Japan, were previously shown to carry a major deletion leading to truncated HrpZ protein product [26]. *Pta *11528 encodes several novel potential T3SS effectors for which no close orthologues have been reported. We also discovered several large genomic regions in *Pta *11528 that do not share detectable nucleotide sequence similarity with previously sequenced *Pseudomonas *genomes. These regions may be horizontally acquired islands that possibly contribute to pathogenicity or epiphytic fitness of *Pta *11528.

## Results and discussion

### Sequencing and assembly of the *Pta *11528 genome

The Illumina sequencing platform provides a cost-effective and rapid means to generate nucleotide sequence data [27-29]. Although this method generates very short sequence reads, several recent studies have demonstrated that it is possible to assemble these short reads into good quality draft genome sequences [30-41].

We generated 12,096,631 pairs of 36-nucleotide reads for a total of 870,957,432 nucleotides. This represents approximately 145X depth of coverage assuming a genome size of six megabases. We used Velvet 0.7.18 [41] to assemble the reads *de novo*. Our resulting assembly had 71 supercontigs of mean length 85,604 nucleotides, an N_50 _number of eight, and N_50 _length of 317,167 nucleotides; that is, the eight longest supercontigs were all at least 317,167 nucleotides long and together covered more than 50% of the predicted genome size of six megabases. The largest supercontig was 606,547 nucleotides long. The total length of the 71 assembled supercontigs was 6,077,921 nucleotides. The G+C content of the assembly was 57.96%, similar to that of the previously sequenced *P. syringae *genomes (Table 1). The sequence data from this project have been deposited at DDBJ/EMBL/GenBank under the accession ACHU00000000. The version described in this paper is the first version, ACHU01000000. The data can also be accessed from the authors' website  and as Additional files submitted with this manuscript. In addition, an interactive genome browser is available from the authors' website .

**Table 1:** Comparison of *Pta *11528 genome properties with those of previously sequenced *P. syringae *genomes [18-20,83-85], [86-93].

**RefSeq accession number**	**Description**	**G+C content (%)**	**Length (nucleotides)**	**Nucleic acid sequence identity to *P. syringae *pv *tabaci *11528 draft assembly (%)**
n. a.	*P. syringae *pv. *tabaci *11528 draft genome assembly	57.96	6,077,921	100

NC_005773	*P. syringae *pv. *phaseolicola *1448A, chromosome	58.01	5,928,787	97.02

NC_007274	*P. syringae *pv. *phaseolicola *1448A large plasmid	55.14	73,661	91.59

NC_004632	*P. syringae *pv. *tomato *str. DC3000 plasmid pDC3000B	56.16	67,473	90.77

NC_007005	*P. syringa*e pv. *syringae *B728a, chromosome	59.23	6,093,698	89.42

NC_004633	*P. syringae *pv. *tomato *str. DC3000 plasmid pDC3000A	58.39	6,397,126	89.36

NC_007275	*P. syringae *pv. *phaseolicola *1448A small plasmid	54.10	131,950	89.09

NC_004578	*P. syringae *pv. *tomato *str. DC3000	58.39	6,397,126	87.65

Percentage identities were calculated over the alignable portion of the genomes using MUMMER [42].

We aligned the 71 *Pta *supercontigs against published complete *Pseudomonas *genome sequences using MUMMER [42]. The *Pta *11528 genome was most similar to that of *Pph *1448A, with 97.02% nucleotide sequence identity over the alignable portions. The next most similar genome was that of *Pto *DC3000, with less than 90% identity (Table 1). This pattern of sequence similarity is consistent with phylogenetic studies that placed strains of *Pta *in the same phylogroup as *Pph *and revealed a relatively distant relationship to *Pto *[21,22].

### Comparison of the protein complement of *Pta *11528 versus *Pph *1448A and other pseudomonads

Using the FgenesB annotation pipeline , we identified 6,057 potential protein-coding genes, of which 5,300 were predicted to encode proteins of at least 100 amino acids long. Of 5,300 predicted *Pta *11528 proteins, 575 (10.8%) had no detectable homology with *Pph *1448A proteins (based on our criterion of an E-value less than 1e-10 using BLASTP). Of these 575 sequences, 303 had no detectable homologues in *Psy *B728a nor *Pto *DC3000. These 303 *Pta*-specific sequences had a median length of 198 amino acids whereas the median length of the 5,300 sequences was 216 amino acids. Automated gene prediction is not infallible and inevitably a subset of the predictions will be incorrect. The reliability of gene predictions is poorer for short sequences than for longer ones. This slight enrichment for very short sequences among the *Pta*-specific gene predictions might be explained by the inclusion of some open reading frames that are not functional genes among those 303. However, many of the predicted proteins showed significant similarity to other proteins in the NCBI NR databases (See Additional file 1: Table S1), confirming that these are likely to be genuine conserved genes.

### Conservation of the T3SS apparatus and T3SS-dependent effectors

**Figure 1:**
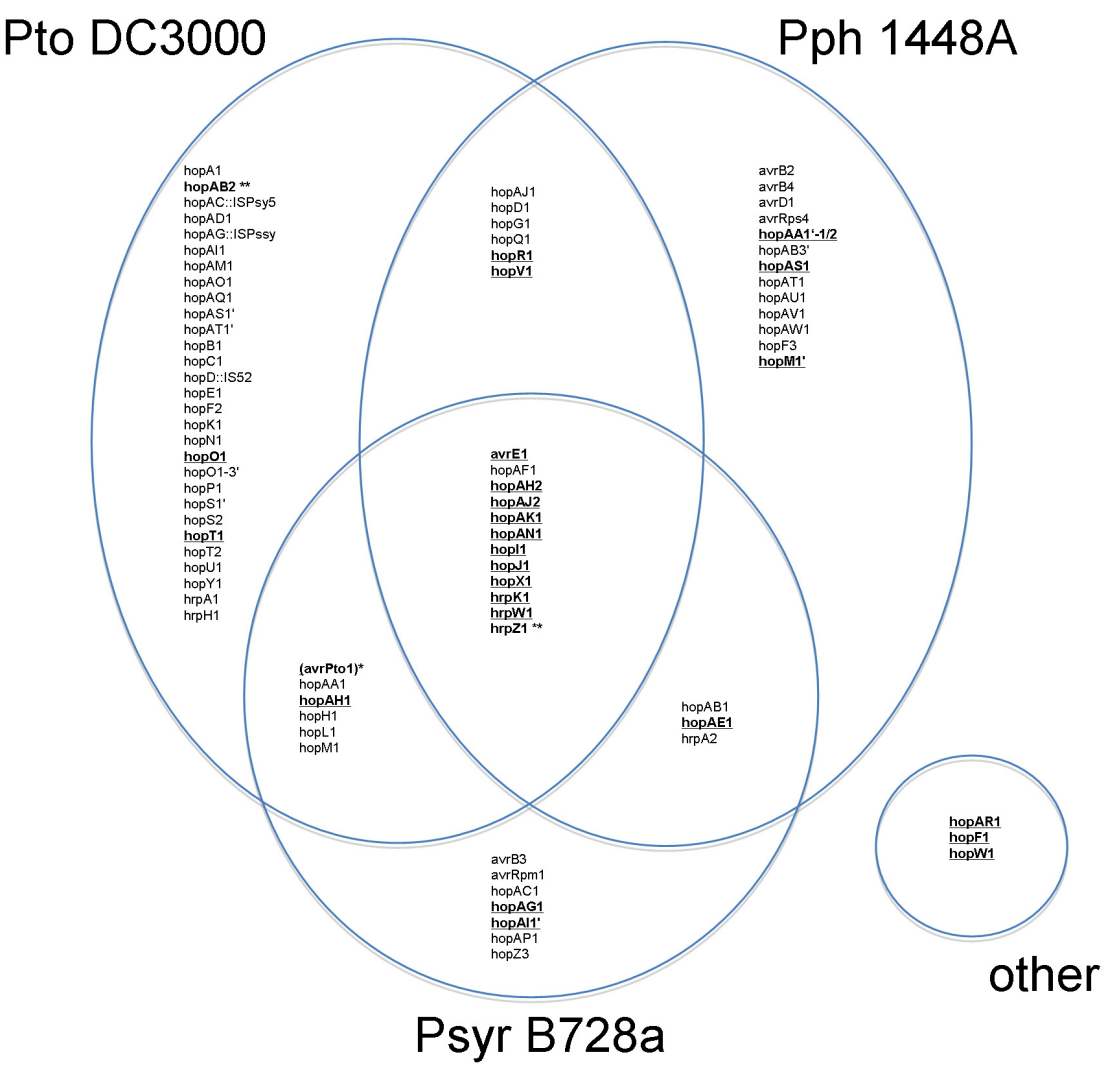
**Comparison of the *hop *gene complements of the three previously fully sequenced *P. syringae *genomes**. Those *hop *genes that are conserved in *Pta *11528 are shown in boldface and underlined. *Pta *11528 also contains three *hop *genes that do not have orthologues in the sequenced genomes: *hopAR1*, *hopF1 *and *hopW1*. * No close homologue of *avrPto1 *was found in *Pta *11528; however, there is a gene encoding a protein that shares 43% amino acid identity with Avr *Pto*1 from *Pto *DC3000. ** In the *Pta *11528 genome *hrpZ1 *appears to be a pseudogene.

The Hop Database (HopDB, ) provides a catalogue of confirmed and predicted *hop *genes [43]. Figure 1 lists the *hop *genes in HopDB for the three previously fully sequenced *P. syringae *genomes. A 'core' set of *hop *genes are conserved in all three previously sequenced pathovars: *avrE1*, *hopAF1*, *hopAH2*, *hopAJ2*, *hopAK1*, *hopAN1*, *hopI1*, *hopJ1*, *hopX1*, *hrpK1*, *hrpW1 *and *hrpZ1*. In addition to this core set, each genome contains additional *hop *genes that are found in only a subset of the sequenced strains. The *Pta *11528 homologues of *hop *genes are listed in Table 2. Figure 1 also indicates those *hop *genes for which a close homologue was found to be encoded in *Pta *11528.

**Table 2:** Homologues of known *hop *genes in *Pta *11528. Homologues were detected by searching the *Pta *11528 FgenesB-predicted protein sequences against HopDB  using BLASTP

**Effector gene**	**Gene in *Pta *11528 genome (location)**	**Hrp-box HMM score (bioinformatic evidence)**	**HrpL-dependent (functional screen)**	**Homologue in Pph 1448A**
*avrE1*	C1E_5333 (1087:342585..346532)	18.24	Yes	PSPPH_1268 (chromosome)

*avrPto1-like*	C1E_2039 (672:104030..104509)	26.02	Yes	None

*hopAB2*	C1E_3975 (955:85214..86053)	None	Yes	None

*hopAE1*	C1E_0512 (174:82348..85077)	17.91	Yes	PSPPH_4326 (chromosome)

*hopAG1*	C1E_2305 (679:71608..73584)	19.67	No	None

*hopAH1*	C1E_2306 (679:74209..74976)	None	No	None

*hopAH2*	C1E_3507 (891:229657..230907)	None	No	PSPPH_3036 (chromosome)

*hopAI1' *(degenerate)	C1E_2307 (679:75143..75466)	24.85	Yes	None

*hopAJ2*	C1E_0586 (174:157540..158877)	None	No	PSPPH_4398 (chromosome)

*hopAK1*	C1E_4764 (1053:316850..318520)	21.71	No	PSPPH_1424 (chromosome)

*hopAN1*	C1E_1908 (661:72932..74221)	None	No	PSPPH_0456 (chromosome)

*hopAR1*	C1E_2036 (672:101352..102155)	15.68	Yes	None

*hopAS1*	C1E_1389 (554:110458..114543)	18.77	No	PSPPH_4736 (chromosome)

*hopI1*	C1E_0551 (174:125987..126916)	21.66	Yes	PSPPH_4366 (chromosome)

*hopM1'*	C1E_5336 (1087:348226..350460)	13.65	Yes	PSPPH1266

*hopO1-1*	C1E_5022 (1087:78582..79433)	18.48	Yes	None

*hopR1*	C1E_3889 (955:5054..6763)	24.65	No	PSPPH_0171

*hopT1-1*	C1E_5021 (1087:77437..78576)	18.48	Yes	None

*hopV1*	C1E_2810 (733:27251..28225)	14.09	No	PSPPH_2351 (chromosome)

*hopW1*	C1E_3964 (955:74860..77184)	10.73	No	None (PSPPH is a truncated HopW1 homologue)

*hopX1*	C1E_5300 (1087:315085..316227)	27.96	No	PSPPH_1296 (chromosome)

*hrpK1*	C1E_5301 (1087:316323..318641)	27.96	No	PSPPH_1295 (chromosome)

*hrpW1*	C1E_5341 (1087:351970..352491)	20.57	No	PSPPH_1264 (chromosome)

*hrpZ1*	C1E_5325 (1087:337056..337478)	19.74	No	PSPPH_1273 (chromosome)
	C1E_5324 (1087:336767..337045)			

The locations of *Pph *1448A homologous genes is indicated, including an indication of whether they are located on the chromosome or on the large plasmid. Also indicated is whether each gene appeared in the functional screen for Hrp-dependent transcription.

**Figure 2:**
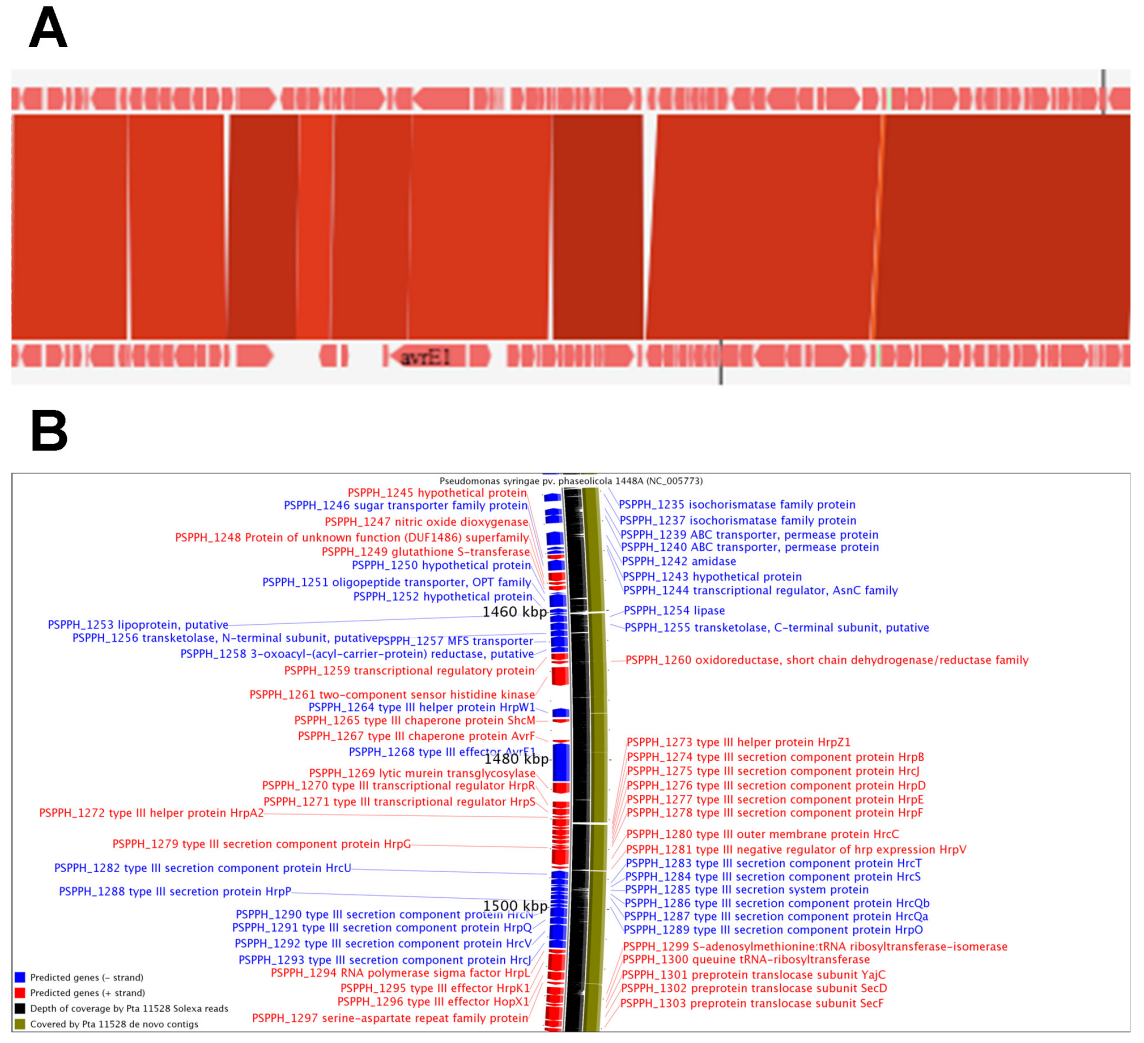
**Conservation of the *Hrp *pathogenicity island between *Pph *1448A and *Pta *11528**. Panel A shows an alignment of the *Pph *1448A *Hrp *pathogenicity island (lower track) against the homologous region in *Pta *11528 (upper track), prepared using GenomeMatcher, which indicates similarity values by colour with dark blue, green, yellow and red representing increasing degrees of similarity [78]. Panel B shows the MAQ [79] alignment of the *Pta *11528 Illumina reads (in black) and the BLASTN [80] alignment of the *Pta *11528 *de novo *assembly (in green) against the *Hrp *region of the *Pph *1448A genome.

In sequenced strains of *P. syringae*, the gene cluster encoding the T3SS apparatus is flanked by collections of effector genes termed the exchangeable effector locus (EEL) and the conserved effector locus (CEL). Together, these three genetic components comprise the *Hrp *pathogenicity island [44]. A core set of *hop *genes is located in the *Hrp *pathogenicity island [44], which is highly conserved between *Pta *11528 and *Pph *1448A (Figure 2), except that in *Pta *11528 there is a deletion in *hrpZ1 *and an insertion in the *hrpV*-*hrcU *intergenic region. The core *hop *genes *avrE1*, *hopAH2*, *hopAJ2*, *hopAK1*, *hopAN1*, *hopI1*, *hopJ1, hopX1 *and *hrpK1 *are conserved in *Pta *11528 and encode intact full-length proteins. *Pta *11528 encodes a full-length HrpW1 protein, albeit with insertions of 69 and 12 nucleotides relative to the *Pph *1448A sequence. However, there is a large deletion in *hrpZ1 *that likely renders it non-functional and *hopAF1 *is completely absent.

Besides the core conserved *hop *genes, the *Pta *11528 genome assembly contains full-length orthologues of *hopR1*, *hopAS1*, *hopAE1 *and *hopV1*, which are also found in *Pph *1448A but are absent from *Psy *B728a and/or *Pto *DC3000.

The *hrpZ1 *gene encodes a harpin, which is not classified as a type III effector because it is not injected directly into host cells. Harpins are characteristically acidic, heat-stable and enriched for glycine, lack cysteine residues [8] and can induce defences in both host and non-host plants [45,46]. HrpZ1 forms pores in the host membrane [47] suggesting a role in translocation of effectors across the host membrane. It also shows sequence-specific protein binding activity [48]. HrpZ1 can induce defences in both host and non-host plants and tobacco has been extensively used as the non-host plant species [45,46]. The inactivation of *hrpZ1 *in *Pta *11528 and other strains of *Pta *[26] may be an adaptive strategy and have been an important process in the stepwise progression towards compatibility, allowing *Pta *11528 to avoid detection by the tobacco host plant. This is reminiscent of the "black holes" and other processes that inactivate genes whose expressed products are detrimental to a pathogenic lifestyle [49,50]. One excellent example is the inactivation of *cadA *in genomes of *Shigella *species as compared to the genome of their closely related but non-pathogenic *Escherichia coli *strain [51,52].

*Pta *11528 contains highly conserved homologues of *hopAB2*, *hopW*, *hopO1-1*, *hopT1-1*, *hopAG1*, *hopAH1*, *hopF1 *and *hopAR1*, which are absent in *Pph *1448A. Although absent from the *Pph *1448A genome, *hopAR1 *and *hopF1 *have been identified in other strains of *Pph *[53-57]. In *Pph *1302A, *hopAR1 *is located on the pathogenicity island *PPH*GI-1, though its genomic location varies between strains [56,57]. *PPH*GI-1 is absent from the *Pph *1448A genome [57]. The *Pta *11528 genome (supercontig 1087) possesses a region of similarity to *PTPH*GI-1, but which contains a substantial number of insertions and deletions (Additional file 2: Figure S1). The *Pta *11528 *hopAR1 *homologue (C1E_2036) is not located in the *PPH*GI-1 region; it falls on supercontig 672 about two kilobases upstream of a gene encoding a protein (C1E_2039) sharing 43% amino acid identity with *Pto *DC3000 *avrPto1*. In contrast to AvrPto1 from *Pto *DC3000, the AvrPto1 homologue (C1E_2039) from *Pta *11528 is not recognised by the plant Pto/Prf system (S. Gimenez Ibanez and J. Rathjen, manuscript in preparation).

The homologues of *hopAG1, hopAH1 *and the degenerate *hopAI1' *are found within a region of the *Pta *11528 genome that shares synteny with the chromosome of *Psy *B728a. This region is also conserved in *Pto *DC3000A, albeit with several deletions and insertions, suggesting that these effector genes are ancestral to the divergence of the pathovars and have been lost in *Pph *1448A rather than having been laterally transferred laterally between *Pta *11528 and *Psy *B728a. In *Pto *DC3000, *hopAG1 *(PSPTO_0901) has been disrupted by an insertion sequence (IS) element. This is consistent with a model of lineage-specific loss of certain ancestral effectors.

**Figure 3:**
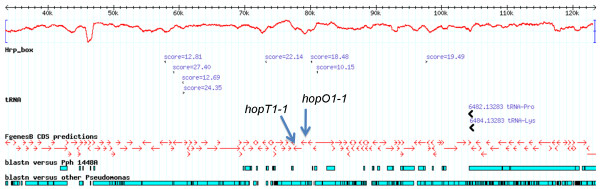
**A 90-kilobase region of the *Pta *11528 genome containing homologues of *hopT1-1 *and *hopO1-1***. The G+C content is indicated by the plot near the top of the figure.

In *Pto *DC3000, *hopO1-1 *and *hopT1-1 *are located on the large plasmid pDC3000A; homologues of these effector-encoding genes are not found in *Pph *1448A. The *Pta *11528 genome contains a three kilobase region of homology to pDC3000 comprising homologues of these two effector genes and a homologue of the ShcO1 chaperone-encoding gene. These three genes are situated in a large (at least 50 kilobase) region of the *Pta *11528 genome that has only limited sequence similarity with *Pph *1448A. Two tRNA genes (tRNA-Pro and tRNA-Lys) are located at the boundary of this region (Figure 3), which would be consistent with this comprising a mobile island.

In plasmid pMA4326B from *P. syringae *pathovar *maculicola *(*Pma*), the *hopW1 *effector gene is immediately adjacent to a three-gene cassette comprising a resolvase, an integrase and *exeA*. This cassette is also found in plasmids and chromosomes of several human-pathogenic Gram-negative bacteria [58]. We found a homologue of this cassette along with a *hopW1 *homologue on supercontig 955 of the *Pta *11528 genome assembly. Stavrinides and Guttman [58] proposed that the boundaries of the cassette lay upstream of the resolvase and upstream of *hopW1*. The presence of this four-gene unit in a completely different location in *Pta *11528 is indeed consistent with the hypothesis that it represents a discrete mobile unit.

**Figure 4:**
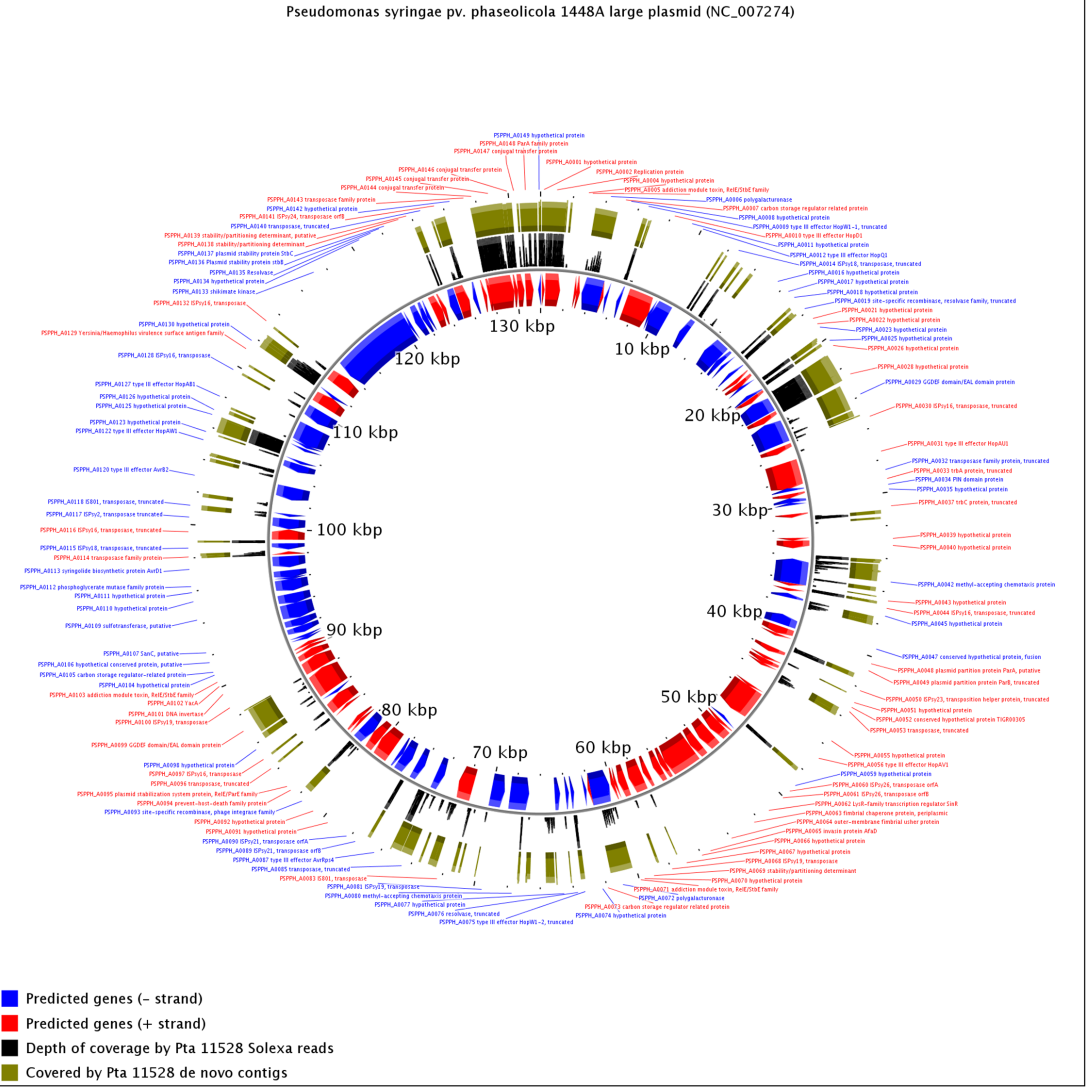
**Limited conservation between the *Pta *11528 genome sequence and the sequence of the *Pph *1448A large plasmid**. The MAQ [79] alignment of the *Pta *11528 Illumina reads is shown in black. The thickness of the black track is proportional to the depth of coverage by Illumina reads. The BLASTN [80] alignment of the *Pta *11528 *de novo *assembly against the plasmid sequence is shown in a green track, with the thickness of this single green track being proportional to sequence identity.

Several *hop *genes are located on the large plasmid of *Pph *1448A. We found no homologues of these genes in *Pta *11528, suggesting that the plasmid is not present in *Pta *11528. Consistent with this, only a small proportion of the plasmid was alignable to our 36-nucleotide Illumina sequence reads (Figure 4). This reveals that a large component of the pathogen's effector arsenal is determined by its complement of plasmids. However, simple loss or gain of a plasmid does not explain all of the differences in effector complement since *Pta *11528 lacks homologues of several *Pph *1448A chromosomally-located effector-encoding *hop *genes *hopG1*, *hopAF1*, *avrB4*, *hopF3 *and *hopAT1 *as well as the non-effector *hopAJ1*. It also lacks homologues of the *Pph *1448A degenerate effector gene *hopAB3*'.

The regions of the *Pph *1448A large plasmid that are apparently conserved in *Pta *11528 include genes encoding the conjugal transfer system, suggesting the presence of one or more plasmids in this strain. We found an open reading frame (C1E_3950, located on supercontig 955 coordinates 59126-60394) encoding a protein with about 97% sequence identity to the RepA proteins characteristically encoded on pT23A-family plasmids (*e.g*. AAW01447; reviewed in [59]), suggesting that this 236 kilobase supercontig might represent a plasmid.

### A functional screen for HrpL-regulated genes

We used a previously described functional screen [60] to complement our bioinformatics-based searches for type III effectors of *Pta *11528. Our functional screen was based on two steps. The first step was employed to identify genes whose expression was regulated by the T3SS alternative sigma factor, HrpL. The second step was used to identify the subset of HrpL-regulated genes that encoded effectors. For *Pta *11528, we employed only the first step to identify candidate effector genes based on induced expression by HrpL. A library was constructed from *Pta *11528 into a broad-host range vector carrying a promoter-less GFP and mobilized into *Pto *lacking its endogenous *hrpL *but conditionally complemented with an arabinose-inducible *hrpL*. We used a fluorescence activated cell sorter (FACS) to select clones that carried HrpL-inducible promoters based on expression of GFP after growth in arabinose. Clones were sequenced and sequences were assembled. Clones representative of assembled supercontigs were verified again for HrpL regulation using FACS. Among the genes whose expression was confirmed to be HrpL-dependent were those encoding effectors *hopAE1*, *hopI1*, *hopAR1*, the *avrPto1*-like gene, *hopF1*, *hopT1-1*, *hopO1-1*, *avrE1*, *hopX1*, and the degenerate *hopM1' and hopAI1' *as well as known T3SS-associated genes *hrpH *(ORF1 of the CEL; [61]) and *hrpW1*. Interestingly, the screen also confirmed HrpL-dependent regulation of genes encoding a major facilitator superfamily (MFS) permease and a putative peptidase (Table 3).

**Table 3:** *Pta *11528 genes confirmed by the functional screen to be under the transcriptional control of HrpL

**Gene**	**Gene in *Pta *11528 gnome**	**Hrp-box HMM score (bioinformatic evidence)**	**Orthologue in Pph 1448A**
*avrE1*	C1E_5333 (1087:342585..346532)	18.24	PSPPH_1268

*avrF*	C1E_5335 (1087:347759..348148)	13.65	PSPPH_1267

*avrPto1*	C1E_2039 (672:104030..104509)	26.02	None

*hopAB2*	C1E_3975 (955:85214..86053)	None	None

*hopAE1*	C1E_0512 (174:82348..85077)	17.91	PSPPH4326 (chromosome)

*hopAI1'*	C1E_2307 (679:75143..75466)	24.85	None

*hopAR1*	C1E_2036 (672:101352..102155)	15.68	None

*hopF1*	C1E_5009 (1087:72050..72664)	22.14	None

*hopI1*	C1E_0551 (174:125987..126916)	21.66	PSPPH_4366

*hopM1'*	(C1E_5336) 1087:348226..350460	13.65	PSPPH_1266

*hopO1-1*	C1E_5022 (1087:78582..79433)	18.48	None

*hopT1-1*	C1E_5021 (1087:77437..78576)	18.48	None

*hopW1*	C1E_3964 (955:74860..77184)	10.73	None

*hopX1*	C1E_5300 (1087:315085..316227)	27.96	PSPPH_1296

*hrpH*	C1E_5332 (1087:341340..342365)	17.46	PSPPH_1269

*hrpW1*	C1E_5339 (1087:351255..351707)	20.57	PSPPH_1264
	C1E_5341 (1087:351970..352491)		

Major facilitator superfamily permease	C1E_4990 1087:59133..60425	12.69	None

Putative M20 peptidase	C1E_1425 (554:155221..156516)	15.05	None

*schF*	C1E_5010 (1087:72735..73127)	22.14	None

*schO1*	C1E_5023 1087:79682..80245	10.50	None

### Other differences in predicted proteomes of *P. syringae *strains

Host range and pathogenicity are likely to be further influenced by genes other than those associated with type III secretion. Virulence determinants in *P. syringae *include toxins as well as epiphytic fitness; that is, the ability to acquire nutrients and survive on the leaf surface [14]. Epiphytic fitness depends on quorum-sensing [62], chemotaxis [63], osmo-protection, extracellular polysaccharides, glycosylation of extracellular structures [64] iron uptake [65] and the ability to form biofilms. Cell-wall-degrading hydrolytic enzymes play a role in virulence in at least some plant-pathogenic pseudomonads [66]). Secretion systems (including type I, type II, type IV, type V, type VI and twin arginine transporter) may also contribute to both virulence and epiphytic fitness [67], whilst multidrug efflux pumps may confer resistance to plant-derived antimicrobials [68].

To identify differences between *Pta *11528 and the previously sequenced *Pph *1448A, *Psy *B728a and *Pto *DC3000 with respect to their repertoires of virulence factors, we performed BLASTP searches between the predicted proteomes. We found no significant differences in the repertoires of secretion systems between the proteomes. However, we found that *Pta *11528 lacks homologues of several *Pph *1448A polysaccharide modifying enzymes (glycosyl transferase PSPPH_0951, polysaccharide lyase PSPPH_1510, glycosyl transferase PSPPH_3642). Conversely, *Pta *11528 encodes two glycosyl transferases (C1E_0355 and C1E_0361) and a thermostable glycosylase (C1E_4802) that do not have homologues in any of the three fully sequenced *P. syringae *genomes. This may imply differences in the extracellular polysaccharide profiles. In contrast to *Pph *1448A, *Pta *11528 lacks homologues of RhsA insecticidal toxins (PSPPH_4042 and PSPPH_4043). However, a tabtoxin biosynthesis gene cluster is found in the *Pta *11528 genome and shows a high degree of conservation with the previously sequenced *Pta *BR2 tabtoxin biosynthesis cluster [69].

*Pta *11528 encodes several enzymes that do not have homologues in any of the three fully sequenced *P. syringae *genomes (Table 4), including a predicted gluconolactonase (C1E_2553), a predicted dienelactone hydrolase (C1E_2589), a predicted nitroreductase (C1E_6026), and a sulphotransferase (C1E_6026). C1E_0903 shares 71.4% amino acid sequence identity with a predicted epoxide hydrolase (YP_745600.1) from *Granulibacter bethesdensis *CGDNIH1 [70] and has a significant match to the epoxide hydrolase N-terminal domain in the Pfam database (PF06441) [71,72]. Epoxide hydrolases are found in *P. aeruginosa *and *P. fluorescens *PfO-1, but not in any other pseudomonads. It is possible that this gene product has a function in detoxification of host-derived secondary metabolites.

**Table 4:** Proteins encoded by the draft *Pta *11528 genome that have no detectable homologues on three previously fully sequenced *P. syringae *genomes.

**Genomic coordinates**	**Locus tag**	**Length (amino acids)**	**Predicted function (FgenesB automated annotation)**
122:73423..74418	C1E_0355	331	Glycosyltransferase involved in cell wall biogenesis

122:82519..83649	C1E_0361	376	Glycosyltransferase

195:60030..60371	C1E_0654	113	Integrase

195:63783..67199	C1E_0659	1138	ATP-binding protein

195:67192..68034	C1E_0660	280	Phosphoadenosine phosphosulfate reductase

195:68040..68927	C1E_0661	295	Serine/threonine protein kinase

256:43490..44116	C1E_0901	208	TetR family ranscriptional regulator. 49% amino acid sequence identity to *G. bethesdensis *GbCGDNIH1_1777 [70].

256:45146..46300	C1E_0903	384	Hydrolases or acyltransferases (alpha/beta hydrolase superfamily)

419:2876..4135	C1E_1014	419	Biotin carboxylase

419:12230..12925	C1E_1023	231	Tabtoxin biosynthesis enzyme, TblA

419:14223..15053	C1E_1025	276	Tetrahydrodipicolinate N-succinyltransferase

554:156634..157137	C1E_1426	167	Histone acetyltransferase HPA2

554:289047..290207	C1E_1572	386	Integrase

554:300540..302342	C1E_1580	600	P-loop ATPase

661:62161..62487	C1E_1896	108	Amine oxidase, flavin-containing

672:22725..23156	C1E_1956	143	Rhs family protein

672:25358..25786	C1E_1961	142	RHS protein

672:118239..118676	C1E_2056	145	Xenobiotic response element family of transcriptional regulator. 37% amino acid sequence identity to *Xylella fastidiosa *PD0954 [81].

672:276912..277349	C1E_2209	145	Histone acetyltransferase HPA2

679:1273..1647	C1E_2227	124	Similar to Mucin-1 precursor (MUC-1)

679:22846..23553	C1E_2251	235	Site-specific recombinases, DNA invertase Pin homologs

679:27901..29442	C1E_2260	513	Phage integrase

679:50793..51164	C1E_2286	123	LacI family transcriptional regulator. 57% amino acid sequence identity to *Rhizobium leguminosarum *plasmid-encoded pRL1201 [82].

679:95310..95744	C1E_2329	144	Tfp pilus assembly protein, major pilin PilA. 42% amino acid sequence identity to P. aeruginosa UniProt:P17838 [74].

684:105988..106806	C1E_2502	272	Histone acetyltransferase HPA2

684:114136..118092	C1E_2506	1318	NTPase (NACHT family)

684:137829..138611	C1E_2527	260	Permeases of the major facilitator superfamily

684:150262..150585	C1E_2541	107	Short-chain dehydrogenase/reductase SDR

684:151896..152792	C1E_2545	298	Nucleoside-diphosphate-sugar

684:160330..161241	C1E_2553	303	Gluconolactonase

684:166181..167533	C1E_2556	450	ASPIC/UnbV domain-containing protein

684:174586..174987	C1E_2563	133	Xenobiotic response element family of transcriptional regulator. 38% amino acid sequence identity to *P. aeruginosa *PACL_0260 [83].

684:178452..178925	C1E_2570	157	Cro/CI family transcriptional regulator. 36% amino acid sequence identity to *Pto *DC3000 PSPTO_2855 [18].

684:192825..193367	C1E_2584	180	Methyl-accepting chemotaxis sensory transducer (C terminus)

684:193364..193720	C1E_2585	118	Methyl-accepting chemotaxis sensory transducer (N terminus)

684:197314..198498	C1E_2589	394	Dienelactone hydrolase

891:113887..115026	C1E_3396	379	Pectate lyase

891:121799..122809	C1E_3401	336	Type II secretory pathway, component PulK

955:27327..28100	C1E_3914	257	DNA-binding HTH domain-containing

955:37638..38900	C1E_3925	420	Outer membrane efflux protein

955:67953..68687	C1E_3957	244	Plasmid stability protein

1053:122043..123497	C1E_4568	484	Phage integrase family protein

1053:127685..128557	C1E_4572	290	Superfamily I DNA or RNA helicase

1053:130636..131022	C1E_4576	128	ATP-dependent DNA helicase, UvrD/Rep family

1053:363558..364328	C1E_4802	256	Thermostable 8-oxoguanine DNA glycosylase

1053:364986..365591	C1E_4804	201	PP-loop superfamily ATPase

1053:365588..366790	C1E_4805	400	Sugar kinase, ribokinase

1053:371237..371890	C1E_4809	217	Restriction endonuclease

1053:384339..385847	C1E_4823	502	ATPase

1053:387166..387564	C1E_4827	132	ATP-dependent DNA helicase, UvrD/Rep family

1053:403085..403480	C1E_4845	131	ATP-dependent DNA helicase, UvrD/Rep family

1053:405835..406488	C1E_4849	217	Restriction endonuclease

1087:462184..462693	C1E_5459	169	Histone acetyltransferase HPA2

1087:463060..463650	C1E_5461	196	Phage collar protein

1087:464015..464851	C1E_5462	278	Sulfotransferase

1087:466231..466944	C1E_5464	237	S-layer domain protein

1087:466941..471797	C1E_5465	1618	Pyrrolo-quinoline quinone

1102:92687..93796	C1E_5711	369	Major facilitator superfamily (MFS) permease

1102:97423..98163	C1E_5715	246	IclR-like transcriptional regulator. 62% amino acid sequence identity to *Acinetobacter baumanii *ACICU_01897 [84].

1160:302149..302877	C1E_6026	242	Nitroreductase

1160:303074..303616	C1E_6027	180	TetR family transcriptional regulator. 56% amino acid sequence identity to *Ralstonia solanacearum *RSc0820 [85].

Protein-coding genes were predicted and automatically annotated using the FgenesB pipeline . Only those proteins are shown for which a predicted function could be proposed.

*Pta *protein C1E_6026 has a significant match to the sulphotransferase domain (Pfam:PF00685). Examples of this protein domain have not been found in other pseudomonads except for *P. fluorescens *PfO-1. Sulphotransferase proteins include flavonyl 3-sulphotransferase, aryl sulphotransferase, alcohol sulphotransferase, estrogen sulphotransferase and phenol-sulphating phenol sulphotransferase. These enzymes are responsible for the transfer of sulphate groups to specific compounds. The sulphotransferase gene (C1E_6026, 82% amino acid identity to *P. fluorescens *Pfl01_0157) overlaps a two kilobase *Pta *11528-specific genomic island that also encodes a phage tail collar-protein encoding gene (C1E_5461, 61% amino acid identity to *P. fluorescens *Pfl01_0155) and an acetyltransferase (C1E_5459, 76% amino acid identity to *P. fluorescens *Pfl01_0148). We speculate that this region has been horizontally acquired in the *Pta *11528 lineage *via *a bacteriophage.

An 80 kilobase region of *Pta *11528 supercontig 684 contains two open reading frames (ORFs) (C1E_2584 and C1E_2585) whose respective predicted protein products show 48 and 55% amino acid identity to the C- and N-termini of a *P. putida *methyl-accepting chemotaxis protein (MCP) (PP_2643) and little similarity to any *P. syringae *protein. Since the N- and C-termini are divided into separate reading frames, this probably represents a degenerate pseudogene. Immediately downstream of these ORFs is a gene (C1E_2583) that specifies a MCP showing greatest sequence identity (70%) to PP_2643 from *P. putida*, whilst sharing only 65% identity to its closest homologue in *P. syringae *(PSPPH_4743). This region also encodes another MCP (C1E_2587) that shares only 50% amino acid identity with any previously sequenced *P. syringae *homologue. It remains to be tested whether these MCPs play a role in pathogenesis and/or epiphytic fitness.

Transcriptional regulators are not normally considered to be virulence factors. However, expression of virulence factors may be coordinated by and dependent on regulators. Moreover, heterologous expression of the RscS regulator was recently shown to be sufficient to transform a fish symbiont into a squid symbiont [73]. *Pta *11528 encodes several predicted transcriptional regulators that are not found in *Pto *DC3000, *Psy *B728a and *Pph *1448A. These include two predicted TetR-like proteins (C1E_0901 and C1E_6027), two predicted xenobiotic response element proteins (C1E_2056 and C1E_2563), a LacI-like protein (C1E_2286), a Cro/CI family protein (C1E_2570) and an IclR family protein (C1E_5715).

*Pta *11528 encodes a novel pilin (C1E_2329) not found in previously sequenced *P. syringae *strains but sharing significant sequence similarity with a type IV pilin from *P. aeruginosa *[74]. Pilin is the major protein component of the type IV pili, which have functions in forming micro-colonies and biofilms, host-cell adhesion, signalling, phage-attachment, DNA uptake and surface motility, and have been implicated as virulence factors in animal-pathogenic bacteria [75]. The precise function of the C1E_2329 pilin is unknown but it may be involved in epiphytic fitness or plant-pathogenesis or could even be involved in an interaction with an insect vector.

### *Pta*-specific genomic islands

We identified 102 genomic regions of at least one kilobase in length which gave no BLASTN matches against previously sequenced *Pseudomonas *genomes (Additional file 3: Table S2). Ten of the *Pta *11528-specific regions are longer than 10 kilobases, the longest being 37.7, 21.8, 18.7, 17.9 and 16.6 kilobases. The 16.6 kilobase region corresponds to the tabtoxin biosynthesis gene cluster [69]. These regions will be good candidates for further study of the genetic basis for association of *Pta *with the tobacco host. For example, several of the islands encode MFS transporters and other efflux proteins that might be involved in protection from plant-derived antimicrobials (Additional file 3: Table S2).

## Conclusion

We have generated a draft complete genome sequence for the *Pta *11528 a pathogen that naturally causes disease in wild tobacco, an important model system for studying plant disease and immunity. From this sequence, combined with a functional screen, we were able to deduce the pathogen's repertoire of T3SS-associated Hop proteins. This has revealed some important differences between *Pta *and other pathovars with respect to the arsenal of T3SS effectors at their disposal for use against the host plant. We also revealed more than a hundred *Pta*-specific genomic regions that are not conserved in any other sequenced *P. syringae*, providing many potential leads for the further study of the *Pta*-tobacco disease system.

## Methods

### Sequence data

The previously published sequences of *P. syringae *pathovar *phaseolicola *1448A [20], *P. syringae *pathovar *syringae *B728a [19], *P. syringae *pathovar *tomato *DC3000 [18] were downloaded from the NCBI FTP site . The NCBI non-redundant (NR) Proteins database was downloaded from the NCBI FTP site  on 10^th ^December 2008.

### *De novo *sequence assembly and annotation

Solexa sequence data were assembled using Velvet 0.7.18 [41]. We used Softberry's FgenesB pipeline  to predict genes encoding rRNAs, tDNAs and proteins. Annotation of protein-coding genes by FgenesB was based on the NCBI NR Proteins database.

### Prediction of HrpL-binding sites (Hrp boxes)

We built a profile hidden Markov model (HMM) based on a multiple sequence alignment of 26 known Hrp boxes from *Pto *DC3000 using *hmmb *from the HMMER 1.8.5 package . DNA sequence was scanned against this profile-HMM using *hmmls *from HMMER 1.8.5 with a bit-score cut-off of 12.0.

### Functional screen for candidate type III effectors

Library preparation and the Flow cytometric-based screen for HrpL-induced genes of *Pta *11528 were done according to [60].

### Visualisation of data

We generated graphical views of genome alignments using CGView [76]. To visualise the annotation draft genome assembly of *Pta*11528, we used the 'gbrowse' Generic Genome Browser [77].

### Library preparation for Illumina sequencing

DNA was prepared from bacteria grown in L-medium using the Puregene Genomic DNA Purification Kit (Gentra Systems, Inc., Minneapolis, USA) according to manufacturer's instructions. A library for Illumina Paired-End sequencing was prepared from 5 mg DNA using a Paired-End DNA Sample Prep Kit (Pe-102-1001, Illumina, Inc., Cambridge, UK). DNA was fragmented by nebulisation for 6 min at a pressure of 32 psi. For end-repair and phosphorylation, sheared DNA was purified using QIAquick Nucleotide Removal Kit (Quiagen, Crawley, UK). The end repaired DNA was A-tailed and ada *Pto*rs were ligated according to manufacturer's instructions.

Size fractionation and purification of ligation products was performed using a 5% polyacrylamide gel run in TBE at 180V for 120 min. Gel slices were cut containing DNA in the 500 to 10 bp range. DNA was than extracted using 0.3 M sodium acetate and 2 mM EDTA [pH 8.0] followed by ethanol precipitation. Using 18 PCR cycles with primer PE1.0 and PE2.0 supplied by Illumina, 5' ada *Pto*r extension and enrichment of the library was performed. The library was finally purified using a QIAquick PCR Purification Kit and adjusted to a concentration of 10 nM in 0.1% Tween. The stock was kept at -20°C until used.

### Sequencing

The flow cell was prepared according to manufacturer's instructions using a Paired-End Cluster Generation Kit (Pe-103-1001) and a Cluster Station. Sequencing reactions were performed on a 1G Genome Analyzer equipped with a Paired-End Module (Illumina, Inc., Cambridge, UK). 5 pM of the library were used to achieve ~20,000 to 25,000 clusters per tile. Capillary sequencing of *avrE*, *HrpW1 *and other individual genes was done on an ABI 3730. PCR products were directly sequenced after treatment with ExoI and SAP. Primer sequences are available upon request from JHC.

### Verification of Illumina sequence data

Three of the core *hop *genes in *Pta *11528 appeared to be degenerate, based on the *de novo *assembly of short Illumina sequence reads. The *avrE1 *gene appeared to have a 20-nucleotide deletion, *hrpZ1 *a 325-nucleotide deletion, whilst *hrpW1 *appeared to have three insertions of 22, 6 and 12 nucleotides. Currently, the reliability of *de novo *sequence assembly from short Illumina reads has not been fully characterised. In particular, repetitive and low-complexity sequence might generate artefacts in assembled supercontigs. Therefore, we checked these putative insertions and deletions by aligning the Illumina sequence reads against the relevant regions of both the *Pph *1448A reference genome sequence and our *Pta *11528 assembly. As an additional control, we also performed Velvet assembies on previously published Illumina short-read data from *Psy *B728a [35]. We found that the B728a *avreE1*, *hrpZ1*, *hrpW1 *and *hopAF1 *were assembled intact [Additional file 4: Figure S2], indicating that there is nothing inherently 'un-assemble-able' about these gene sequences. Sequence alignment is much more robust than *de novo *assembly and is not subject to assembly artefacts. The alignments supported the presence of a large deletion in *hrpZ1*. However, the alignments were not consistent with the assembly for *avrE1 *and *hrpW1*. Therefore, we amplified the *Pta *11528 *avrE1 *and *hrpW1 *genes by PCR and verified their sequences by capillary sequencing [Additional file 5: Table S3]. This confirmed that the apparent deletion in *avrE1 *was an artefact of the *de novo *assembly and that the *avrE1 *sequence encodes a full-length protein product. Furthermore, transient expression of *avrE1 *in *N. benthamiana *induces cell death (S. Gimenez Ibanez and J. Rathjen, unpublished). Capillary sequencing also confirmed that the *de novo *assembly of *hrpW1 *was incorrect and that *Pta *11528 encodes a full-length HrpW1 protein, albeit with repetitive sequence insertions of 69 and 12 nucleotides relative to the *Pph *1448A sequence.

The absence of *hopAF1 *from Pta 11528 is supported not only by the *de novo *assembly, but also by the absence of aligned (unassembled) reads. As an additional control for the degeneracy of *hopAF1 *and *hrpZ1*, we performed the same bioinformatics and sequencing protocols to *Psy *B728a [35] and recovered *hopAF1 *and *hrpZ1 *intact in the *de novo *assembly assembly (Additional file 4: Figure S1).

### Sequence data

In addition to the data available from Genbank accession ACHU00000000, the Velvet assembly and predicted protein sequences are provided in FastA format in Additional files 6 and Additional file 7.

### Bioinformatics tools

We used GenomeMatcher [78] for generating and visualising whole-genome alignments. For aligning short Illumina sequence reads against a reference genome, we used MAQ [79] and for other sequence alignments and searches we used BLAST [80]. We used previously published complete genomes as reference sequences for comparative analyses [81-85].

## List of abbreviations

CEL: conserved effector locus; EEL: exchangeable effector locus; HMM: hidden Markov model; HopDB: Hop database; MCP: methyl-accepting chemotaxis protein; PCR: polymerase chain reaction; *Pma*: *Pseudomonas syringae *pathovar *maculicola*; *Pph: Pseudomonas syringae *pathovar *phaseolicola*; *PPH*GI-1: *Pph *genomic island 1; *Pta: Pseudomonas syringae *pathovar *tabaci*; *Psy: Pseudomonas syringae *pathovar *syringae*; *Pto: Pseudomonas syringae *pathovar *tomato*; VIGS: virus-induced gene silencing; IS: insertion sequence.

## Authors' contributions

DJS and DM performed the sequence assembly and all subsequent bioinformatics analyses. SGI prepared the DNA libraries and performed phenotypic characterisation of *Pta *11528. JHC performed the functional screen for HrpL-dependent genes and analysed the resulting data. JR, DJS and JHC conceived of the study, participated in its design. DJS, JR and JHC wrote the manuscript. All authors read, approved and made contributions to the manuscript.

## Supplementary Material

Additional file 1 **Table S1. Proteins encoded in the *Pta *11528 draft with no detectable homologue in previously sequenced *P. syringae *genomes (*Pto *DC3000, *Psy *B728a and *Pph *1448A)**. Proteins implicated in mobile genetic elements are shaded in cyan. Other proteins for which a function could be predicted by homology are shaded in yellow. Click here for file

Additional file 2 **Figure S1. Alignment of the *Pph *1302A PPHGI-1 pathogenicity island against the *Pta *11528 genome assembly**. The *Pta *11528 genome sequence is in the upper track, aligned against the *Pph *1302A PPHGI-1 pathogenicity island sequence. (Genbank: AJ870974). Click here for file

Additional file 3 **Table S2. Regions of the *Pta *11528 genome with no nucleotide sequence similarity to the genomes of *Pto *DC3000, *Pss *B728a and *Pph *1448A**. Click here for file

Additional file 4 **Figure S2. The *avrE1*, *hrpZ1*, *hrpW1 *and *hopAF1 *genes are recovered intact in a *de novo *sequence assembly of Illumina short sequence reads from *Psy *B728a**. We assembled a 40 × deep dataset (reference 35) of paired 36-nucleotide reads from *Psy *B728a genomic DNA using Velvet 0.7.18, using the same protocol as for the *Pta *11528 data. Panel A shows the MAQ alignment of the B728a Illumina reads (in black) and the *blastn *alignment of the B728a *de novo *assembly (in green) against the *avrE1 *gene in the B728a genome. Panel B shows the alignments against *hrpZ1*. Panel C shows the alignments against *hrpW1*. Panel D shows the alignments against *hopAF1*. Click here for file

Additional file 5 **Table S3. Verification of predicted genes by capillary sequencing**. We verified a selection of genes predicted from the Illumina-based Pta11528 genome sequence assembly by capillary sequencing of cloned PCR products. Sequence reads were trimmed to remove poor quality nucleotide calls and the trimmed sequences were aligned against predicted proteins using TBLASTN. Click here for file

Additional file 6 **The *Pta *11528 draft genome assembly, in FastA format, generated using Velvet** Click here for file

Additional file 7 **Protein sequences, in FastA format, predicted in the *Pta *11528 draft genome assembly using FgenesB** Click here for file
