# A draft genome sequence and functional screen reveals the repertoire of type III secreted proteins of *Pseudomonas syringae *pathovar *tabaci *11528

**DOI:** 10.1186/1471-2164-10-569

**Published:** 2009-12-01

**Authors:** David J Studholme, Selena Gimenez Ibanez, Daniel MacLean, Jeffery L Dangl, Jeff H Chang, John P Rathjen

**Affiliations:** 1 The Sainsbury Laboratory, Norwich, NR4 7UH, UK; 2 Department of Biology, CB# 3280, Coker Hall, The University of North Carolina at Chapel Hill, Chapel Hill, North Carolina 27599-3280, USA; 3 Department of Botany and Plant Pathology, Oregon State University, 2082 Cordley Hall, Corvallis, OR 97331, USA; 4 Center for Genome Research and Biocomputing, Oregon State University, 2082 Cordley Hall, Corvallis, OR 97331, USA

## Correction

After the publication of this work [1], we became aware of several errors in our descriptions of proteins associated with the type III secretion system (T3SS) of *P. syringae *pathovar *tabaci *(*Pta*) strain 11528.

As mentioned in the text of the article [1], *Pta *11528 encodes a full-length homologue of HopAB2. Therefore, in Figure 1, HopAB2 should not have been marked with asterisks. A corrected summary of the Hop protein repertoire in *Pta *11528 is shown in Figure 1 of this Correction. We confirmed the presence of a full-length *hopAB2 *using capillary sequencing. Unfortunately, in the draft assembly of Illumina sequence data presented in the paper [1], there was a mis-assembly error that resulted in deletion of 271 nucleotides from the 5' end of the *hopAB2 *gene. This type of error is, unfortunately, not uncommon in assemblies of short sequence reads, though recent versions of the Velvet assembly software seem to be less prone to such errors. We are currently generating 454 GS-FLX sequence data from *Pta *11528 genomic DNA and hope to make public an improved genome assembly and annotation in due course.

**Figure 1:**
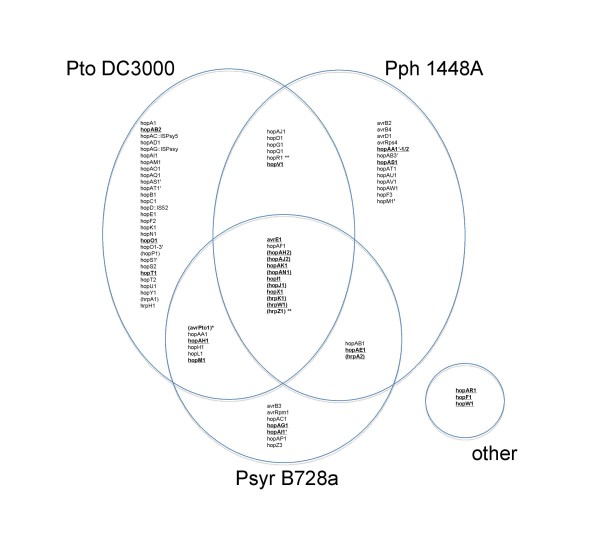
**Comparison of the complement of *Pta *11528 genes encoding candidate T3SS substrates with those of the three fully sequenced *P. syringae *genomes**. In addition to validated *hop *genes, also are included are several genes for T3SS helper proteins (*hrpA1*, *hrpA2*, *hrpZ1*, *hrpW1*, *hrpK1 *and *hopP1*) and several former candidates that are probably not true *hop *genes (*hopAH2*, *hopJ1*, *hopAJ2 *and *hopAN1*) (the HopDB website, http://www.pseudomonas-syringae.org); these genes are indicated by parentheses. Those genes that are conserved in *Pta *11528 are shown in boldface and underlined. *Pta *11528 also contains three *hop *genes that do not have orthologues in the three sequenced genomes: *hopAR1*, *hopF1 *and *hopW1*. * No close homologue of *avrPto1 *was found in *Pta *11528; however, there is a gene encoding a protein that shares 43% amino acid identity with Avr*Pto*1 from *Pto *DC3000. ** In the *Pta *11528 genome *hrpZ1 *and *hopR1 *appear to be degenerate pseudogenes.

*Pta *11528 encodes a full-length homologue of T3SS helper protein HrpA2 (Locus tag C1E_5326 in our annotation; RefSeq: ZP_05641290.1). Therefore *hrpA2 *should have been shown in boldface and underlined in Figure 1 of the manuscript [1]. This has been remedied in Figure 1 of this Correction

Contrary to the original manuscript [1], HopR1 is degenerate in *Pta *11528. In the *Pta *11528 draft assembly, the *hopR1 *gene was split into at least two open reading frames (RefSeq: ZP_05639788.1, ZP_05639787.1; locus tags C1E_3889, C1E_3890) suggesting that is a degenerate pseudogene. We confirmed the presence of an internal stop codon in *hopR1 *using capillary sequencing. This degeneracy should have been indicated by marking *hopR1 *with a double asterisk (**) in Figure 1. This has been remedied in Figure 1 of this Correction

*Pta *11528 encodes a full-length HopM1 homologue (RefSeq: ZP_05641297.1; locus tag C1E_5336; GenBank: ACR46722.1). The fact that HopM1 is intact and not degenerate should have been indicated in Figure 1 (by highlighting *hopM1 *in boldface and underlined) in the original manuscript [1]. This has been remedied in Figure 1 of this Correction.

We regret any inconvenience caused by these errors and are grateful to Dr Magdalen Lindeberg for bringing them to our attention.
