# Supplementary material for: A draft genome sequence and functional screen reveals the repertoire of type III secreted proteins of Pseudomonas syringae pathovar tabaci 11528
Source: BMC Genomics. 2009 Aug 24;10:395. doi: 10.1186/1471-2164-10-395 (PMC2745422; doi:10.1186/1471-2164-10-395)
Supplement: Additional file 1 — Table S1. Proteins encoded in the Pta 11528 draft with no detectable homologue in previously sequenced P. syringae genomes (Pto DC3000, Psy B728a and Pph 1448A). Proteins implicated in mobile genetic elements are shaded in cyan. Other proteins for which a function could be predicted by homology are shaded in yellow. [file 1471-2164-10-395-S1.html]

Comparison of Pto-and-Pss-and-Pph.faa and Pta11528.faa


# Proteins encoded by *Pta* 11528 with no homologue in *Pto* DC3000, *Pss* B728a and *Pph* 1448A

| Genes | Genes | Genes | Genes | Genes |
| --- | --- | --- | --- | --- |
| 1 | 554:272835..273335 | 1516 | 164 | gi|118591172|ref|ZP\_01548571.1| 50S ribosomal protein L14 |
| 3 | 46:3..789 | C1E\_0013 | 262 | gi|104779986|ref|YP\_606484.1| hypothetical protein PSEEN0746 |
| 4 | 88:4332..4814 | C1E\_0019 | 160 | Putative gene predicted by FgenesB |
| 6 | 88:7565..7918 | C1E\_0024 | 117 | Putative gene predicted by FgenesB |
| 11 | 98:73713..74684 | C1E\_0104 | 323 | Putative gene predicted by FgenesB |
| 12 | 98:77991..78878 | C1E\_0109 | 295 | Putative gene predicted by FgenesB |
| 13 | 98:78891..79826 | C1E\_0110 | 311 | gi|111021415|ref|YP\_704387.1| hypothetical protein RHA1\_ro04443 |
| 14 | 98:83238..84323 | C1E\_0112 | 361 | gi|111021417|ref|YP\_704389.1| hypothetical protein RHA1\_ro04445 |
| 16 | 98:122401..122721 | C1E\_0151 | 106 | Putative gene predicted by FgenesB |
| 19 | 98:200803..201381 | C1E\_0226 | 192 | gi|145301485|ref|YP\_001144324.1| hypothetical protein ASA\_P5G106 |
| 20 | 98:211627..212097 | C1E\_0241 | 156 | Putative gene predicted by FgenesB |
| 22 | 122:70893..73106 | C1E\_0354 | 737 | Putative gene predicted by FgenesB |
| 23 | 122:73423..74418 | C1E\_0355 | 331 | Glycosyltransferases involved in cell wall biogenesis COG0463 Glycosyltransferases involved in cell wall biogenesis |
| 24 | 122:74415..74753 | C1E\_0356 | 112 | gi|194290471|ref|YP\_002006378.1| hypothetical protein RALTA\_A2383 |
| 25 | 122:82519..83649 | C1E\_0361 | 376 | Glycosyltransferase COG0438 Glycosyltransferase |
| 29 | 174:121313..121714 | C1E\_0547 | 133 | Putative gene predicted by FgenesB |
| 32 | 195:60030..60371 | C1E\_0654 | 113 | Integrase COG0582 Integrase |
| 33 | 195:60228..60575 | C1E\_0655 | 115 | Putative gene predicted by FgenesB |
| 34 | 195:60572..61657 | C1E\_0656 | 361 | gi|77456971|ref|YP\_346476.1| hypothetical protein Pfl01\_0744 |
| 35 | 195:61755..62216 | C1E\_0657 | 153 | Putative gene predicted by FgenesB |
| 36 | 195:62954..63877 | C1E\_0658 | 307 | gi|121583152|ref|YP\_973593.1| hypothetical protein Pnap\_4583 |
| 37 | 195:63783..67199 | C1E\_0659 | 1138 | gi|121583153|ref|YP\_973594.1| putative ATP-binding protein |
| 38 | 195:67192..68034 | C1E\_0660 | 280 | gi|121583154|ref|YP\_973595.1| phosphoadenosine phosphosulfate reductase |
| 39 | 195:68040..68927 | C1E\_0661 | 295 | gi|32471645|ref|NP\_864638.1| serine/threonine protein kinase |
| 40 | 195:68945..70300 | C1E\_0662 | 451 | Uncharacterized conserved protein COG1479 Uncharacterized conserved protein |
| 41 | 195:75719..77272 | C1E\_0664 | 517 | gi|77957027|ref|ZP\_00821095.1| hypothetical protein YberA\_01002741 |
| 42 | 195:77269..79344 | C1E\_0665 | 691 | gi|124262720|ref|YP\_001023190.1| hypothetical protein Mpe\_B0179 |
| 43 | 195:79652..80974 | C1E\_0666 | 440 | gi|78189815|ref|YP\_380153.1| hypothetical protein Cag\_1860 |
| 48 | 256:35217..35525 | C1E\_0892 | 102 | Putative gene predicted by FgenesB |
| 49 | 256:40822..41487 | C1E\_0898 | 221 | gi|149908533|ref|ZP\_01897195.1| hypothetical protein PE36\_15944 |
| 51 | 256:42340..43254 | C1E\_0900 | 304 | gi|157411390|gb|ABV54348.1| hypothetical protein |
| 52 | 256:43490..44116 | C1E\_0901 | 208 | Transcriptional regulator COG1309 Transcriptional regulator |
| 53 | 256:44175..45149 | C1E\_0902 | 324 | gi|114328442|ref|YP\_745599.1| putative cytoplasmic protein |
| 54 | 256:45146..46300 | C1E\_0903 | 384 | Predicted hydrolases or acyltransferases (alpha/beta hydrolase superfamily) COG0596 Predicted hydrolases or acyltransferases (alpha/beta hydrolase superfamily) |
| 55 | 256:57751..58071 | C1E\_0918 | 106 | gi|213971559|ref|ZP\_03399669.1| hypothetical protein PSPTOT1\_4657 |
| 59 | 256:116905..117243 | C1E\_0977 | 112 | Putative gene predicted by FgenesB |
| 63 | 419:2876..4135 | C1E\_1014 | 419 | Biotin carboxylase COG0439 Biotin carboxylase |
| 64 | 419:4132..4611 | C1E\_1015 | 159 | gi|30060191|gb|AAP13070.1| putative membrane protein |
| 65 | 419:4566..5096 | C1E\_1016 | 176 | gi|30060191|gb|AAP13070.1| putative membrane protein |
| 67 | 419:10842..11651 | C1E\_1021 | 269 | gi|21693557|gb|AAM75344.1|AF519896\_1 tabtoxin biosynthetic region hypothetical protein |
| 68 | 419:11599..12213 | C1E\_1022 | 204 | gi|21693557|gb|AAM75344.1|AF519896\_1 tabtoxin biosynthetic region hypothetical protein |
| 69 | 419:12230..12925 | C1E\_1023 | 231 | gi|2506922|sp|P31850|TBLA\_PSESZ Tabtoxin biosynthesis enzyme tabtoxin biosynthesis enzyme TblA |
| 70 | 419:14223..15053 | C1E\_1025 | 276 | Tetrahydrodipicolinate N-succinyltransferase COG2171 Tetrahydrodipicolinate N-succinyltransferase |
| 77 | 479:145750..146418 | C1E\_1183 | 222 | gi|153889859|ref|ZP\_02010963.1| hypothetical protein ObacDRAFT\_2574 |
| 78 | 479:147271..148398 | C1E\_1185 | 375 | gi|114330127|ref|YP\_746349.1| hypothetical protein Neut\_0096 |
| 88 | 554:156634..157137 | C1E\_1426 | 167 | Histone acetyltransferase HPA2 and related acetyltransferases COG0454 Histone acetyltransferase HPA2 and related acetyltransferases |
| 90 | 554:165731..166177 | C1E\_1434 | 148 | Putative gene predicted by FgenesB |
| 91 | 554:166216..166695 | C1E\_1435 | 159 | Putative gene predicted by FgenesB |
| 93 | 554:276328..276648 | C1E\_1557 | 106 | Putative gene predicted by FgenesB |
| 94 | 554:289047..290207 | C1E\_1572 | 386 | Integrase COG0582 Integrase |
| 95 | 554:293773..294228 | C1E\_1575 | 151 | gi|77166473|ref|YP\_344998.1| hypothetical protein Noc\_3026 |
| 96 | 554:294354..294992 | C1E\_1576 | 212 | Putative gene predicted by FgenesB |
| 97 | 554:295288..296598 | C1E\_1577 | 436 | gi|59713723|ref|YP\_206498.1| hypothetical protein VF\_A0540 |
| 98 | 554:297052..297876 | C1E\_1578 | 274 | gi|186471663|ref|YP\_001862981.1| hypothetical protein Bphy\_6925 |
| 99 | 554:298124..300304 | C1E\_1579 | 726 | gi|109899890|ref|YP\_663145.1| hypothetical protein Patl\_3589 |
| 100 | 554:300540..302342 | C1E\_1580 | 600 | Predicted P-loop ATPase COG4928 Predicted P-loop ATPase |
| 101 | 554:302342..303097 | C1E\_1581 | 251 | gi|26250335|ref|NP\_756375.1| hypothetical protein c4513 |
| 102 | 554:303076..304404 | C1E\_1582 | 442 | gi|169796893|ref|YP\_001714686.1| hypothetical protein ABAYE2885 |
| 103 | 554:306489..307493 | C1E\_1584 | 334 | gi|200387411|ref|ZP\_03214023.1| hypothetical protein SeV\_B2346 |
| 104 | 554:307714..308073 | C1E\_1585 | 119 | Putative gene predicted by FgenesB |
| 105 | 554:308802..309194 | C1E\_1586 | 130 | Putative gene predicted by FgenesB |
| 106 | 554:309251..309817 | C1E\_1587 | 188 | Putative gene predicted by FgenesB |
| 109 | 554:354015..354389 | C1E\_1636 | 124 | gi|148545935|ref|YP\_001266037.1| hypothetical protein Pput\_0689 |
| 110 | 649:17796..19022 | C1E\_1763 | 408 | gi|163797804|ref|ZP\_02191750.1| hypothetical protein BAL199\_22217 |
| 113 | 661:62161..62487 | C1E\_1896 | 108 | gi|71734218|ref|YP\_272749.1| amine oxidase, flavin-containing |
| 116 | 672:18795..19778 | C1E\_1952 | 327 | gi|194565434|ref|YP\_002100456.1| hypothetical protein BDAG\_03748 |
| 117 | 672:22200..22715 | C1E\_1955 | 171 | gi|170721705|ref|YP\_001749393.1| hypothetical protein PputW619\_2526 |
| 118 | 672:22725..23156 | C1E\_1956 | 143 | Rhs family protein COG3209 Rhs family protein |
| 120 | 672:24879..25358 | C1E\_1960 | 159 | gi|161506541|ref|YP\_001573662.1| hypothetical protein Bmul\_6209 |
| 121 | 672:25358..25786 | C1E\_1961 | 142 | gi|161506542|ref|YP\_001573663.1| RHS protein |
| 122 | 672:26592..27185 | C1E\_1962 | 197 | gi|170721709|ref|YP\_001749397.1| hypothetical protein PputW619\_2532 |
| 123 | 672:27199..27570 | C1E\_1963 | 123 | gi|83749882|ref|ZP\_00946847.1| Core protein |
| 131 | 672:70562..71017 | C1E\_2003 | 151 | gi|71733264|ref|YP\_272447.1| hypothetical protein PSPPH\_0138 |
| 134 | 672:98049..98609 | C1E\_2033 | 186 | Uncharacterized protein conserved in bacteria COG3575 Uncharacterized protein conserved in bacteria |
| 135 | 672:102533..103237 | C1E\_2037 | 234 | gi|152988629|ref|YP\_001350411.1| hypothetical protein PSPA7\_5075 |
| 136 | 672:105301..105762 | C1E\_2041 | 153 | Putative gene predicted by FgenesB |
| 138 | 672:113351..113755 | C1E\_2051 | 134 | Putative gene predicted by FgenesB |
| 139 | 672:118239..118676 | C1E\_2056 | 145 | Predicted transcriptional regulator COG2944 Predicted transcriptional regulator |
| 140 | 672:118679..118996 | C1E\_2057 | 105 | Putative gene predicted by FgenesB |
| 148 | 672:276912..277349 | C1E\_2209 | 145 | Histone acetyltransferase HPA2 and related acetyltransferases COG0454 Histone acetyltransferase HPA2 and related acetyltransferases |
| 150 | 672:289762..290181 | C1E\_2222 | 139 | gi|197295498|ref|YP\_002154039.1| hypothetical protein BCAS0661C |
| 152 | 672:291027..291458 | C1E\_2224 | 143 | gi|197295498|ref|YP\_002154039.1| hypothetical protein BCAS0661C |
| 153 | 679:1273..1647 | C1E\_2227 | 124 | gi|194666913|ref|XP\_001250651.2| PREDICTED: similar to Mucin-1 precursor (MUC-1) (Polymorphic epithelial mucin) (PEM) (PEMT) (Episialin) (Tumor-associated mucin) (Carcinoma-associated mucin) (Tumor-associated epithelial membrane antigen) (EMA) (H23AG) (Peanut-reactive urinary mucin) (PUM) (B |
| 154 | 679:22343..22657 | C1E\_2250 | 104 | gi|77459725|ref|YP\_349232.1| hypothetical protein Pfl01\_3503 |
| 155 | 679:22846..23553 | C1E\_2251 | 235 | Site-specific recombinases, DNA invertase Pin homologs COG1961 Site-specific recombinases, DNA invertase Pin homologs |
| 158 | 679:24325..24690 | C1E\_2254 | 121 | Putative gene predicted by FgenesB |
| 159 | 679:24690..25163 | C1E\_2255 | 157 | Putative gene predicted by FgenesB |
| 161 | 679:25486..26739 | C1E\_2257 | 417 | Uncharacterized conserved protein COG4983 Uncharacterized conserved protein |
| 164 | 679:27901..29442 | C1E\_2260 | 513 | gi|71908115|ref|YP\_285702.1| Phage integrase |
| 165 | 679:50793..51164 | C1E\_2286 | 123 | Transcriptional regulators COG1609 Transcriptional regulators |
| 170 | 679:88361..89767 | C1E\_2322 | 468 | FOG: TPR repeat, SEL1 subfamily COG0790 FOG: TPR repeat, SEL1 subfamily |
| 171 | 679:89872..90330 | C1E\_2323 | 152 | Putative gene predicted by FgenesB |
| 172 | 679:95310..95744 | C1E\_2329 | 144 | Tfp pilus assembly protein, major pilin PilA COG4969 Tfp pilus assembly protein, major pilin PilA |
| 175 | 684:27116..27595 | C1E\_2421 | 159 | Putative gene predicted by FgenesB |
| 181 | 684:103020..103904 | C1E\_2500 | 294 | Putative gene predicted by FgenesB |
| 182 | 684:105988..106806 | C1E\_2502 | 272 | Histone acetyltransferase HPA2 and related acetyltransferases COG0454 Histone acetyltransferase HPA2 and related acetyltransferases |
| 183 | 684:107263..107859 | C1E\_2503 | 198 | gi|163754668|ref|ZP\_02161790.1| hypothetical protein KAOT1\_17273 |
| 184 | 684:108255..109205 | C1E\_2504 | 316 | gi|37528687|ref|NP\_932032.1| hypothetical protein plu4879 |
| 185 | 684:109553..113494 | C1E\_2505 | 1313 | gi|104781858|ref|YP\_608356.1| hypothetical protein PSEEN2777 |
| 186 | 684:114136..118092 | C1E\_2506 | 1318 | Predicted NTPase (NACHT family) COG5635 Predicted NTPase (NACHT family) |
| 189 | 684:123738..124244 | C1E\_2514 | 168 | Putative gene predicted by FgenesB |
| 190 | 684:124987..126378 | C1E\_2516 | 463 | gi|197123729|ref|YP\_002135680.1| hypothetical protein AnaeK\_3335 |
| 191 | 684:126368..128083 | C1E\_2517 | 571 | gi|38637815|ref|NP\_942789.1| hypothetical protein PHG151 |
| 194 | 684:137829..138611 | C1E\_2527 | 260 | Permeases of the major facilitator superfamily COG0477 Permeases of the major facilitator superfamily |
| 195 | 684:142452..142781 | C1E\_2531 | 109 | Putative gene predicted by FgenesB |
| 196 | 684:142867..144540 | C1E\_2532 | 557 | gi|197787299|ref|YP\_002209967.1| conserved hypothetical protein |
| 197 | 684:144799..145284 | C1E\_2533 | 161 | gi|146276858|ref|YP\_001167017.1| hypothetical protein Rsph17025\_0806 |
| 198 | 684:145288..146565 | C1E\_2534 | 425 | gi|146276859|ref|YP\_001167018.1| hypothetical protein Rsph17025\_0807 |
| 203 | 684:150262..150585 | C1E\_2541 | 107 | gi|196238562|ref|ZP\_03137337.1| short-chain dehydrogenase/reductase SDR |
| 206 | 684:151896..152792 | C1E\_2545 | 298 | Nucleoside-diphosphate-sugar epimerases COG0451 Nucleoside-diphosphate-sugar epimerases |
| 207 | 684:160330..161241 | C1E\_2553 | 303 | Gluconolactonase COG3386 Gluconolactonase |
| 208 | 684:166181..167533 | C1E\_2556 | 450 | gi|116625141|ref|YP\_827297.1| ASPIC/UnbV domain-containing protein |
| 209 | 684:170153..170638 | C1E\_2559 | 161 | Uncharacterized conserved protein COG1430 Uncharacterized conserved protein |
| 210 | 684:172555..173349 | C1E\_2561 | 264 | gi|26990408|ref|NP\_745833.1| hypothetical protein PP\_3700 |
| 211 | 684:174586..174987 | C1E\_2563 | 133 | Predicted transcriptional regulators COG1396 Predicted transcriptional regulators |
| 213 | 684:175842..176558 | C1E\_2566 | 238 | Putative gene predicted by FgenesB |
| 214 | 684:176623..176949 | C1E\_2567 | 108 | Putative gene predicted by FgenesB |
| 216 | 684:178452..178925 | C1E\_2570 | 157 | gi|26991154|ref|NP\_746579.1| Cro/CI family transcriptional regulator |
| 217 | 684:180949..182670 | C1E\_2573 | 573 | gi|82617198|emb|CAI64105.1| hypothetical protein |
| 218 | 684:182770..183552 | C1E\_2574 | 260 | gi|91200149|emb|CAJ73193.1| hypothetical protein |
| 220 | 684:184032..184523 | C1E\_2576 | 163 | Putative gene predicted by FgenesB |
| 221 | 684:185349..185774 | C1E\_2577 | 141 | Putative gene predicted by FgenesB |
| 222 | 684:185946..187847 | C1E\_2578 | 633 | Putative gene predicted by FgenesB |
| 223 | 684:188197..188988 | C1E\_2579 | 263 | Putative gene predicted by FgenesB |
| 224 | 684:190991..191497 | C1E\_2581 | 168 | Putative gene predicted by FgenesB |
| 226 | 684:192825..193367 | C1E\_2584 | 180 | gi|26989362|ref|NP\_744787.1| methyl-accepting chemotaxis sensory transducer |
| 227 | 684:193364..193720 | C1E\_2585 | 118 | gi|167034181|ref|YP\_001669412.1| methyl-accepting chemotaxis sensory transducer |
| 229 | 684:197314..198498 | C1E\_2589 | 394 | Dienelactone hydrolase and related enzymes COG0412 Dienelactone hydrolase and related enzymes |
| 230 | 684:198538..198891 | C1E\_2590 | 117 | Putative gene predicted by FgenesB |
| 232 | 684:275222..275761 | C1E\_2667 | 179 | Putative gene predicted by FgenesB |
| 233 | 684:275841..276167 | C1E\_2668 | 108 | Putative gene predicted by FgenesB |
| 235 | 684:305361..305918 | C1E\_2696 | 185 | Putative gene predicted by FgenesB |
| 239 | 684:317682..321773 | C1E\_2712 | 1363 | gi|186473776|ref|YP\_001861118.1| hypothetical protein Bphy\_4982 |
| 240 | 684:322270..324030 | C1E\_2713 | 586 | gi|167456887|ref|ZP\_02323103.1| hypothetical protein A2cp1DRAFT\_1523 |
| 241 | 684:324035..324748 | C1E\_2714 | 237 | Putative gene predicted by FgenesB |
| 243 | 684:378102..378755 | C1E\_2778 | 217 | Putative gene predicted by FgenesB |
| 245 | 733:48910..49776 | C1E\_2840 | 288 | Putative gene predicted by FgenesB |
| 247 | 733:78772..79236 | C1E\_2861 | 154 | Putative gene predicted by FgenesB |
| 249 | 832:95528..95959 | C1E\_2970 | 143 | Putative gene predicted by FgenesB |
| 253 | 832:200880..201572 | C1E\_3083 | 230 | Putative gene predicted by FgenesB |
| 259 | 891:68249..68755 | C1E\_3346 | 168 | Putative gene predicted by FgenesB |
| 261 | 891:113887..115026 | C1E\_3396 | 379 | Pectate lyase COG3866 Pectate lyase |
| 262 | 891:121799..122809 | C1E\_3401 | 336 | Type II secretory pathway, component PulK COG3156 Type II secretory pathway, component PulK |
| 264 | 891:140212..140646 | C1E\_3422 | 144 | Putative gene predicted by FgenesB |
| 267 | 891:175249..175725 | C1E\_3448 | 158 | Putative gene predicted by FgenesB |
| 274 | 928:10724..11071 | C1E\_3694 | 115 | Putative gene predicted by FgenesB |
| 275 | 928:17435..17749 | C1E\_3701 | 104 | gi|66043318|ref|YP\_233159.1| hypothetical protein Psyr\_0047 |
| 276 | 928:22297..23775 | C1E\_3706 | 492 | gi|126659740|ref|ZP\_01730868.1| hypothetical protein CY0110\_23281 |
| 277 | 928:136675..137145 | C1E\_3809 | 156 | gi|70728392|ref|YP\_258141.1| hypothetical protein PFL\_1010 |
| 282 | 955:13486..13803 | C1E\_3896 | 105 | Putative gene predicted by FgenesB |
| 284 | 955:27327..28100 | C1E\_3914 | 257 | DNA-binding HTH domain-containing proteins COG2771 DNA-binding HTH domain-containing proteins |
| 285 | 955:28152..29267 | C1E\_3915 | 371 | gi|170697494|ref|ZP\_02888585.1| hypothetical protein BamIOP4010DRAFT\_0647 |
| 286 | 955:31248..31643 | C1E\_3918 | 131 | Uncharacterized protein conserved in bacteria COG3544 Uncharacterized protein conserved in bacteria |
| 288 | 955:37638..38900 | C1E\_3925 | 420 | gi|146307237|ref|YP\_001187702.1| outer membrane efflux protein |
| 289 | 955:50887..51417 | C1E\_3935 | 176 | gi|116622258|ref|YP\_824414.1| hypothetical protein Acid\_3152 |
| 290 | 955:51433..52014 | C1E\_3936 | 193 | gi|153801432|ref|ZP\_01956018.1| conserved hypothetical protein |
| 292 | 955:53244..53756 | C1E\_3939 | 170 | gi|145301485|ref|YP\_001144324.1| hypothetical protein ASA\_P5G106 |
| 297 | 955:56890..57210 | C1E\_3947 | 106 | gi|38257040|ref|NP\_940694.1| Orf8 |
| 298 | 955:61005..61358 | C1E\_3951 | 117 | Putative gene predicted by FgenesB |
| 299 | 955:66935..67357 | C1E\_3955 | 140 | gi|49188565|ref|YP\_025663.1| hypothetical protein PMA4326A45 |
| 300 | 955:67384..67956 | C1E\_3956 | 190 | gi|49188564|ref|YP\_025662.1| hypothetical protein PMA4326A44 |
| 301 | 955:67953..68687 | C1E\_3957 | 244 | gi|38257083|ref|NP\_940737.1| stability protein |
| 302 | 955:72399..72890 | C1E\_3961 | 163 | Putative gene predicted by FgenesB |
| 308 | 1039:64946..65386 | C1E\_4182 | 146 | Putative gene predicted by FgenesB |
| 314 | 1039:129983..131953 | C1E\_4256 | 656 | Putative gene predicted by FgenesB |
| 317 | 1039:134549..135163 | C1E\_4260 | 204 | gi|213967435|ref|ZP\_03395583.1| hypothetical protein PSPTOT1\_3778 |
| 319 | 1039:137505..137858 | C1E\_4264 | 117 | Putative gene predicted by FgenesB |
| 320 | 1039:138108..138584 | C1E\_4265 | 158 | Putative gene predicted by FgenesB |
| 322 | 1039:172436..172876 | C1E\_4309 | 146 | Putative gene predicted by FgenesB |
| 335 | 1053:104398..104979 | C1E\_4554 | 193 | gi|70728944|ref|YP\_258683.1| hypothetical protein PFL\_1557 |
| 336 | 1053:108152..109219 | C1E\_4558 | 355 | Putative gene predicted by FgenesB |
| 337 | 1053:111001..111462 | C1E\_4560 | 153 | Putative gene predicted by FgenesB |
| 338 | 1053:111487..111834 | C1E\_4561 | 115 | gi|21242949|ref|NP\_642531.1| hypothetical protein XAC2214 |
| 339 | 1053:112168..113292 | C1E\_4562 | 374 | gi|197123729|ref|YP\_002135680.1| hypothetical protein AnaeK\_3335 |
| 340 | 1053:113543..115261 | C1E\_4563 | 572 | gi|190895190|ref|YP\_001985483.1| hypothetical protein RHECIAT\_PC0000863 |
| 341 | 1053:115432..117246 | C1E\_4564 | 604 | Putative gene predicted by FgenesB |
| 342 | 1053:117480..118004 | C1E\_4565 | 174 | Putative gene predicted by FgenesB |
| 343 | 1053:117976..118593 | C1E\_4566 | 205 | Putative gene predicted by FgenesB |
| 344 | 1053:118669..122046 | C1E\_4567 | 1125 | gi|94311109|ref|YP\_584319.1| hypothetical protein Rmet\_2171 |
| 345 | 1053:122043..123497 | C1E\_4568 | 484 | gi|119946224|ref|YP\_943904.1| phage integrase family protein |
| 346 | 1053:123994..125037 | C1E\_4569 | 347 | gi|16763096|ref|NP\_458713.1| hypothetical protein STY4631 |
| 347 | 1053:125039..126217 | C1E\_4570 | 392 | gi|56414689|ref|YP\_151764.1| hypothetical protein SPA2585 |
| 348 | 1053:126214..126900 | C1E\_4571 | 228 | Putative gene predicted by FgenesB |
| 349 | 1053:127685..128557 | C1E\_4572 | 290 | Superfamily I DNA and RNA helicases COG0210 Superfamily I DNA and RNA helicases |
| 350 | 1053:129461..129775 | C1E\_4574 | 104 | Putative gene predicted by FgenesB |
| 351 | 1053:129937..130434 | C1E\_4575 | 165 | Putative gene predicted by FgenesB |
| 352 | 1053:130636..131022 | C1E\_4576 | 128 | gi|213970986|ref|ZP\_03399107.1| ATP-dependent DNA helicase, UvrD/Rep family |
| 353 | 1053:131116..131574 | C1E\_4577 | 152 | Putative gene predicted by FgenesB |
| 354 | 1053:131662..132558 | C1E\_4578 | 298 | gi|21244022|ref|NP\_643604.1| hypothetical protein XAC3296 |
| 359 | 1053:146549..147847 | C1E\_4596 | 432 | gi|186474382|ref|YP\_001863353.1| FRG domain-containing protein |
| 360 | 1053:148022..149089 | C1E\_4597 | 355 | Putative gene predicted by FgenesB |
| 362 | 1053:149537..150328 | C1E\_4599 | 263 | gi|34496298|ref|NP\_900513.1| hypothetical protein CV\_0843 |
| 363 | 1053:150404..151306 | C1E\_4600 | 300 | Putative gene predicted by FgenesB |
| 364 | 1053:151371..151727 | C1E\_4601 | 118 | Putative gene predicted by FgenesB |
| 365 | 1053:151976..153244 | C1E\_4602 | 422 | gi|213970594|ref|ZP\_03398720.1| hypothetical protein PSPTOT1\_2837 |
| 367 | 1053:154749..155555 | C1E\_4605 | 268 | gi|158423769|ref|YP\_001525061.1| hypothetical protein AZC\_2145 |
| 369 | 1053:262741..263172 | C1E\_4709 | 143 | Putative gene predicted by FgenesB |
| 370 | 1053:271421..271957 | C1E\_4718 | 178 | Putative gene predicted by FgenesB |
| 373 | 1053:350044..351162 | C1E\_4786 | 372 | FOG: TPR repeat, SEL1 subfamily COG0790 FOG: TPR repeat, SEL1 subfamily |
| 376 | 1053:353894..354739 | C1E\_4790 | 281 | gi|104782308|ref|YP\_608806.1| hypothetical protein PSEEN3258 |
| 377 | 1053:355233..357002 | C1E\_4791 | 589 | gi|163854509|ref|YP\_001628807.1| hypothetical protein Bpet0205 |
| 378 | 1053:357185..357559 | C1E\_4792 | 124 | gi|148550219|ref|YP\_001270321.1| hypothetical protein Pput\_5017 |
| 379 | 1053:358378..359103 | C1E\_4794 | 241 | gi|153000097|ref|YP\_001365778.1| hypothetical protein Shew185\_1568 |
| 380 | 1053:362932..363558 | C1E\_4801 | 208 | gi|116006770|ref|YP\_787954.1| hypothetical protein pBP136\_p34 |
| 381 | 1053:363558..364328 | C1E\_4802 | 256 | Thermostable 8-oxoguanine DNA glycosylase COG1059 Thermostable 8-oxoguanine DNA glycosylase |
| 382 | 1053:364325..364909 | C1E\_4803 | 194 | Putative gene predicted by FgenesB |
| 383 | 1053:364986..365591 | C1E\_4804 | 201 | Predicted PP-loop superfamily ATPase COG0603 Predicted PP-loop superfamily ATPase |
| 384 | 1053:365588..366790 | C1E\_4805 | 400 | Sugar kinases, ribokinase family COG0524 Sugar kinases, ribokinase family |
| 385 | 1053:366793..367134 | C1E\_4806 | 113 | Putative gene predicted by FgenesB |
| 386 | 1053:367367..369295 | C1E\_4807 | 642 | gi|167361405|ref|ZP\_02295988.1| conserved hypothetical protein |
| 387 | 1053:369629..371053 | C1E\_4808 | 474 | gi|169344607|ref|ZP\_02865573.1| hypothetical protein CPC\_A0224 |
| 388 | 1053:371237..371890 | C1E\_4809 | 217 | Restriction endonuclease COG1403 Restriction endonuclease |
| 389 | 1053:373453..373848 | C1E\_4812 | 131 | gi|26990418|ref|NP\_745843.1| hypothetical protein PP\_3710 |
| 390 | 1053:373903..374349 | C1E\_4813 | 148 | gi|213969790|ref|ZP\_03397925.1| hypothetical protein PSPTOT1\_1216 |
| 391 | 1053:374440..374823 | C1E\_4814 | 127 | gi|213969791|ref|ZP\_03397926.1| hypothetical protein PSPTOT1\_1217 |
| 393 | 1053:375518..377353 | C1E\_4816 | 611 | gi|121611799|ref|YP\_999606.1| hypothetical protein Veis\_4900 |
| 394 | 1053:377543..378181 | C1E\_4817 | 212 | Putative gene predicted by FgenesB |
| 395 | 1053:378208..378966 | C1E\_4818 | 252 | gi|177666441|ref|ZP\_02942236.1| hypothetical protein PCC8801DRAFT\_3119 |
| 397 | 1053:383462..384286 | C1E\_4822 | 274 | gi|149910982|ref|ZP\_01899612.1| hypothetical protein PE36\_08111 |
| 398 | 1053:384339..385847 | C1E\_4823 | 502 | gi|77462592|ref|YP\_352096.1| ATPase |
| 401 | 1053:387166..387564 | C1E\_4827 | 132 | gi|213970986|ref|ZP\_03399107.1| ATP-dependent DNA helicase, UvrD/Rep family |
| 403 | 1053:387790..388155 | C1E\_4829 | 121 | gi|213970982|ref|ZP\_03399103.1| hypothetical protein PSPTOT1\_4224 |
| 404 | 1053:388437..389303 | C1E\_4830 | 288 | Putative gene predicted by FgenesB |
| 407 | 1053:390320..391528 | C1E\_4834 | 402 | gi|67153547|ref|ZP\_00415292.1| hypothetical protein AvinDRAFT\_5818 |
| 408 | 1053:391702..396003 | C1E\_4835 | 1433 | gi|71907006|ref|YP\_284593.1| hypothetical protein Daro\_1374 |
| 410 | 1053:401685..402332 | C1E\_4843 | 215 | gi|191164129|ref|ZP\_03025998.1| hypothetical protein GM21DRAFT\_3410 |
| 411 | 1053:402589..403092 | C1E\_4844 | 167 | gi|213970972|ref|ZP\_03399093.1| hypothetical protein PSPTOT1\_4214 |
| 412 | 1053:403085..403480 | C1E\_4845 | 131 | gi|213970986|ref|ZP\_03399107.1| ATP-dependent DNA helicase, UvrD/Rep family |
| 413 | 1053:405158..405835 | C1E\_4848 | 225 | Putative gene predicted by FgenesB |
| 414 | 1053:405835..406488 | C1E\_4849 | 217 | Restriction endonuclease COG1403 Restriction endonuclease |
| 415 | 1053:406607..407221 | C1E\_4850 | 204 | gi|134288364|ref|YP\_001110527.1| hypothetical protein Bcep1808\_6837 |
| 416 | 1053:407364..409697 | C1E\_4851 | 777 | gi|148977726|ref|ZP\_01814287.1| hypothetical purine NTPase |
| 417 | 1053:409672..410337 | C1E\_4852 | 221 | gi|98978971|gb|ABF59983.1| hypothetical protein QG7\_0013 |
| 418 | 1053:410318..411550 | C1E\_4853 | 410 | gi|98978972|gb|ABF59984.1| conserved hypothetical protein |
| 419 | 1053:412195..413715 | C1E\_4854 | 506 | Putative gene predicted by FgenesB |
| 420 | 1053:415134..416093 | C1E\_4855 | 319 | gi|168136318|ref|ZP\_02579547.1| hypothetical protein BcerB\_15332 |
| 421 | 1053:416676..418202 | C1E\_4857 | 508 | Putative gene predicted by FgenesB |
| 422 | 1053:418348..418788 | C1E\_4858 | 146 | gi|148550222|ref|YP\_001270324.1| hypothetical protein Pput\_5020 |
| 423 | 1053:418804..419220 | C1E\_4859 | 138 | gi|213970971|ref|ZP\_03399092.1| hypothetical protein PSPTOT1\_4213 |
| 425 | 1053:419690..420322 | C1E\_4861 | 210 | gi|157962451|ref|YP\_001502485.1| hypothetical protein Spea\_2630 |
| 426 | 1053:420443..420958 | C1E\_4862 | 171 | gi|213970989|ref|ZP\_03399110.1| hypothetical protein PSPTOT1\_4231 |
| 427 | 1053:421060..421758 | C1E\_4863 | 232 | Putative gene predicted by FgenesB |
| 428 | 1053:422487..423101 | C1E\_4864 | 204 | Putative gene predicted by FgenesB |
| 429 | 1053:423670..424272 | C1E\_4865 | 200 | gi|17975120|ref|NP\_536642.1| hypothetical protein K139p15 |
| 430 | 1053:425086..425490 | C1E\_4866 | 134 | Putative gene predicted by FgenesB |
| 432 | 1087:183..716 | C1E\_4905 | 177 | Putative gene predicted by FgenesB |
| 438 | 1087:10875..11312 | C1E\_4920 | 145 | Putative gene predicted by FgenesB |
| 441 | 1087:23115..23480 | C1E\_4938 | 121 | Putative gene predicted by FgenesB |
| 442 | 1087:24572..25057 | C1E\_4941 | 161 | Putative gene predicted by FgenesB |
| 446 | 1087:28710..29171 | C1E\_4949 | 153 | Putative gene predicted by FgenesB |
| 448 | 1087:32655..33341 | C1E\_4953 | 228 | gi|213971942|ref|ZP\_03400041.1| hypothetical protein PSPTOT1\_4929 |
| 449 | 1087:33563..34231 | C1E\_4954 | 222 | Putative gene predicted by FgenesB |
| 452 | 1087:43141..43557 | C1E\_4966 | 138 | Putative gene predicted by FgenesB |
| 453 | 1087:43554..44375 | C1E\_4967 | 273 | Putative gene predicted by FgenesB |
| 455 | 1087:44614..44940 | C1E\_4969 | 108 | Putative gene predicted by FgenesB |
| 456 | 1087:45563..45919 | C1E\_4971 | 118 | Putative gene predicted by FgenesB |
| 459 | 1087:58477..58791 | C1E\_4988 | 104 | Putative gene predicted by FgenesB |
| 460 | 1087:58713..59174 | C1E\_4989 | 153 | Putative gene predicted by FgenesB |
| 463 | 1087:70639..71220 | C1E\_5006 | 193 | gi|213972165|ref|ZP\_03400252.1| hypothetical protein PSPTOT1\_2819 |
| 470 | 1087:80451..80819 | C1E\_5024 | 122 | gi|55978498|gb|AAV68748.1| unknown |
| 481 | 1087:89521..90237 | C1E\_5042 | 238 | Putative gene predicted by FgenesB |
| 499 | 1087:134052..134390 | C1E\_5105 | 112 | Putative gene predicted by FgenesB |
| 500 | 1087:149624..149938 | C1E\_5124 | 104 | Putative gene predicted by FgenesB |
| 503 | 1087:166321..166749 | C1E\_5143 | 142 | Putative gene predicted by FgenesB |
| 506 | 1087:198105..198623 | C1E\_5176 | 172 | Putative gene predicted by FgenesB |
| 507 | 1087:210429..210797 | C1E\_5190 | 122 | Putative gene predicted by FgenesB |
| 508 | 1087:210998..211483 | C1E\_5191 | 161 | Putative gene predicted by FgenesB |
| 509 | 1087:219577..219972 | C1E\_5201 | 131 | Putative gene predicted by FgenesB |
| 510 | 1087:223312..223797 | C1E\_5205 | 161 | Putative gene predicted by FgenesB |
| 514 | 1087:252600..253277 | C1E\_5228 | 225 | Putative gene predicted by FgenesB |
| 515 | 1087:261817..262212 | C1E\_5240 | 131 | Putative gene predicted by FgenesB |
| 519 | 1087:282694..283593 | C1E\_5262 | 299 | Putative gene predicted by FgenesB |
| 521 | 1087:298209..299681 | C1E\_5278 | 490 | gi|26248882|ref|NP\_754922.1| hypothetical protein c3040 |
| 523 | 1087:308132..308668 | C1E\_5292 | 178 | Putative gene predicted by FgenesB |
| 524 | 1087:325201..325995 | C1E\_5307 | 264 | Putative gene predicted by FgenesB |
| 525 | 1087:330128..330685 | C1E\_5314 | 185 | Putative gene predicted by FgenesB |
| 528 | 1087:350559..351170 | C1E\_5338 | 203 | Putative gene predicted by FgenesB |
| 530 | 1087:353906..354343 | C1E\_5344 | 145 | gi|213968287|ref|ZP\_03396431.1| hypothetical protein PSPTOT1\_0678 |
| 534 | 1087:385039..385395 | C1E\_5378 | 118 | Putative gene predicted by FgenesB |
| 537 | 1087:386402..386815 | C1E\_5382 | 137 | Putative gene predicted by FgenesB |
| 544 | 1087:440837..441631 | C1E\_5440 | 264 | Putative gene predicted by FgenesB |
| 547 | 1087:457978..458757 | C1E\_5456 | 259 | Membrane-fusion protein COG0845 Membrane-fusion protein |
| 548 | 1087:458754..460058 | C1E\_5457 | 434 | gi|77456374|ref|YP\_345879.1| hypothetical protein Pfl01\_0146 |
| 549 | 1087:460058..462154 | C1E\_5458 | 698 | gi|77456375|ref|YP\_345880.1| peptidase M50 |
| 550 | 1087:462184..462693 | C1E\_5459 | 169 | Histone acetyltransferase HPA2 and related acetyltransferases COG0454 Histone acetyltransferase HPA2 and related acetyltransferases |
| 551 | 1087:462694..463005 | C1E\_5460 | 103 | gi|77456382|ref|YP\_345887.1| hypothetical protein Pfl01\_0154 |
| 552 | 1087:463060..463650 | C1E\_5461 | 196 | Microcystin-dependent protein COG4675 Microcystin-dependent protein |
| 553 | 1087:464015..464851 | C1E\_5462 | 278 | gi|77456385|ref|YP\_345890.1| sulfotransferase |
| 554 | 1087:464895..466241 | C1E\_5463 | 448 | gi|119489070|ref|ZP\_01621976.1| hypothetical protein L8106\_22246 |
| 555 | 1087:466231..466944 | C1E\_5464 | 237 | gi|192809377|ref|ZP\_03038052.1| S-layer domain protein |
| 556 | 1087:466941..471797 | C1E\_5465 | 1618 | gi|170719861|ref|YP\_001747549.1| Pyrrolo-quinoline quinone |
| 557 | 1087:478323..478733 | C1E\_5470 | 136 | Putative gene predicted by FgenesB |
| 561 | 1087:510978..511289 | C1E\_5510 | 103 | Putative gene predicted by FgenesB |
| 562 | 1087:544202..544711 | C1E\_5540 | 169 | Putative gene predicted by FgenesB |
| 563 | 1087:546899..547399 | C1E\_5542 | 166 | Putative gene predicted by FgenesB |
| 565 | 1087:547648..547953 | C1E\_5544 | 101 | Putative gene predicted by FgenesB |
| 574 | 1102:92687..93796 | C1E\_5711 | 369 | Permeases of the major facilitator superfamily COG0477 Permeases of the major facilitator superfamily |
| 575 | 1102:97423..98163 | C1E\_5715 | 246 | Transcriptional regulator COG1414 Transcriptional regulator |
| 579 | 1160:59176..59550 | C1E\_5786 | 124 | Putative gene predicted by FgenesB |
| 584 | 1160:241407..241802 | C1E\_5962 | 131 | Predicted small integral membrane protein COG5478 Predicted small integral membrane protein |
| 585 | 1160:302149..302877 | C1E\_6026 | 242 | Nitroreductase COG0778 Nitroreductase |
| 586 | 1160:303074..303616 | C1E\_6027 | 180 | Transcriptional regulator COG1309 Transcriptional regulator |
| 588 | 1160:376576..377310 | C1E\_6101 | 244 | gi|213969764|ref|ZP\_03397899.1| hypothetical protein PSPTOT1\_1190 |
| 589 | 1160:379366..379923 | C1E\_6104 | 185 | gi|213971093|ref|ZP\_03399213.1| hypothetical protein PSPTOT1\_1398 |
| 590 | 1160:379920..380630 | C1E\_6105 | 236 | gi|120556622|ref|YP\_960973.1| hypothetical protein Maqu\_3717 |
| 591 | 1160:380479..383868 | C1E\_6106 | 1129 | gi|120556230|ref|YP\_960581.1| hypothetical protein Maqu\_3323 |
