# Supplementary figures and images for: A draft genome sequence and functional screen reveals the repertoire of type III secreted proteins of Pseudomonas syringae pathovar tabaci 11528
Source: BMC Genomics. 2009 Aug 24;10:395. doi: 10.1186/1471-2164-10-395 (PMC2745422; doi:10.1186/1471-2164-10-395)

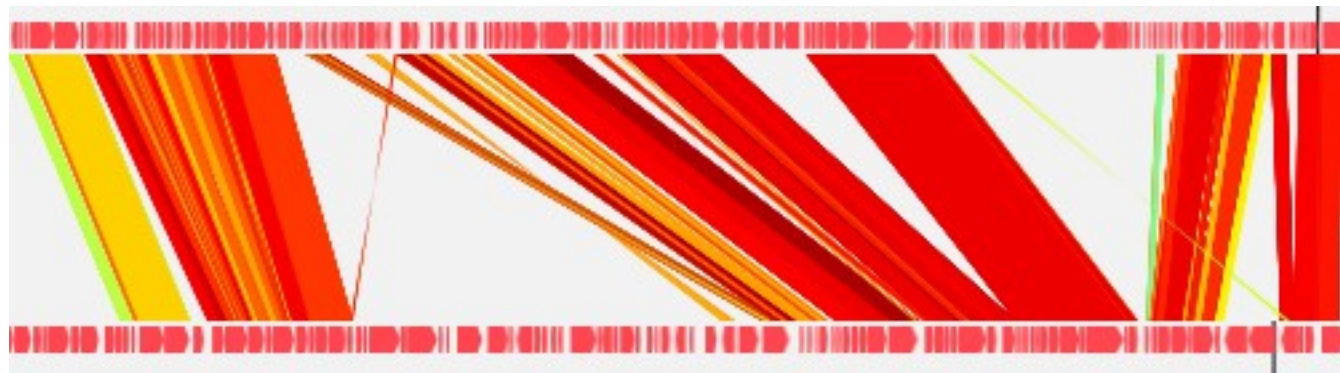

Supplement: Additional file 2 — Figure S1. Alignment of the Pph 1302A PPHGI-1 pathogenicity island against the Pta 11528 genome assembly. The Pta 11528 genome sequence is in the upper track, aligned against the Pph 1302A PPHGI-1 pathogenicity island sequence. (Genbank: AJ870974). [file 1471-2164-10-395-S2.pdf]

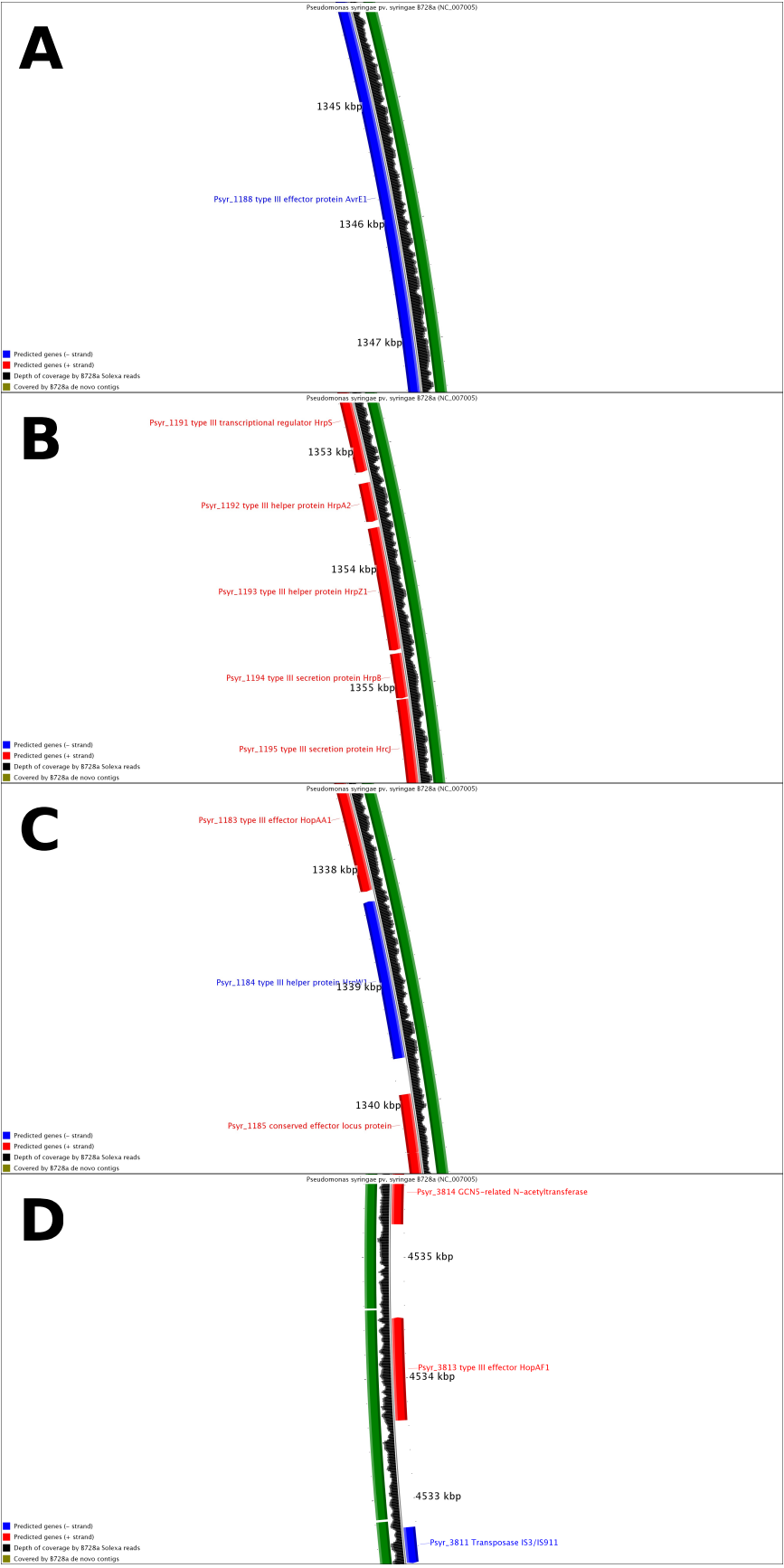

Supplement: Additional file 4 — Figure S2. The avrE1, hrpZ1, hrpW1 and hopAF1 genes are recovered intact in a de novo sequence assembly of Illumina short sequence reads from Psy B728a. We assembled a 40 × deep dataset (reference 35) of paired 36-nucleotide reads from Psy B728a genomic DNA using Velvet 0.7.18, using the same protocol as for the Pta 11528 data. Panel A shows the MAQ alignment of the B728a Illumina reads (in black) and the blastn alignment of the B728a de novo assembly (in green) against the avrE1 gene in the B728a genome. Panel B shows the alignments against hrpZ1. Panel C shows the alignments against hrpW1. Panel D shows the alignments against hopAF1. [file 1471-2164-10-395-S4.pdf]
