# Supplementary material for: A draft genome sequence and functional screen reveals the repertoire of type III secreted proteins of Pseudomonas syringae pathovar tabaci 11528
Source: BMC Genomics. 2009 Aug 24;10:395. doi: 10.1186/1471-2164-10-395 (PMC2745422; doi:10.1186/1471-2164-10-395)
Supplement: Additional file 3 — Table S2. Regions of the Pta 11528 genome with no nucleotide sequence similarity to the genomes of Pto DC3000, Pss B728a and Pph 1448A. [file 1471-2164-10-395-S3.html]

Pseudomonas\_syringae\_pv\_tabaci\_11528: regions with no blast hits


| Rank | Length | Position | Genes |
| --- | --- | --- | --- |
| 1 | 37734 | 684:113661..151394 | **C1E\_2506** ( Predicted NTPase (NACHT family) COG5635 Predicted NTPase (NACHT family) );  **C1E\_2507** ( gi|77459152|ref|YP\_348658.1| cointegrate resolution protein T );  **C1E\_2508** ( Integrase COG0582 Integrase );  **C1E\_2509** ( gi|28871887|ref|NP\_794506.1| hypothetical protein PSPTO\_4771 );  **C1E\_2510** ( gi|28871887|ref|NP\_794506.1| hypothetical protein PSPTO\_4771 );  **C1E\_2511** ( Putative gene predicted by FgenesB );  **C1E\_2512** ( NADH:flavin oxidoreductases, Old Yellow Enzyme family COG1902 NADH:flavin oxidoreductases, Old Yellow Enzyme family );  **C1E\_2513** ( Putative gene predicted by FgenesB );  **C1E\_2514** ( Putative gene predicted by FgenesB );  **C1E\_2515** ( gi|213970979|ref|ZP\_03399100.1| DNA-binding protein );  **C1E\_2516** ( gi|197123729|ref|YP\_002135680.1| hypothetical protein AnaeK\_3335 );  **C1E\_2517** ( gi|38637815|ref|NP\_942789.1| hypothetical protein PHG151 );  **C1E\_2518** ( Putative gene predicted by FgenesB );  **C1E\_2519** ( Choline dehydrogenase and related flavoproteins COG2303 Choline dehydrogenase and related flavoproteins );  **C1E\_2520** ( Uncharacterized homolog of gamma-carboxymuconolactone decarboxylase subunit COG0599 Uncharacterized homolog of gamma-carboxymuconolactone decarboxylase subunit );  **C1E\_2521** ( 3-hydroxyisobutyrate dehydrogenase and related beta-hydroxyacid dehydrogenases COG2084 3-hydroxyisobutyrate dehydrogenase and related beta-hydroxyacid dehydrogenases );  **C1E\_2522** ( NAD-dependent aldehyde dehydrogenases COG1012 NAD-dependent aldehyde dehydrogenases );  **C1E\_2523** ( NAD-dependent aldehyde dehydrogenases COG1012 NAD-dependent aldehyde dehydrogenases );  **C1E\_2524** ( Permeases of the major facilitator superfamily COG0477 Permeases of the major facilitator superfamily );  **C1E\_2525** ( Transcriptional regulators COG1802 Transcriptional regulators );  **C1E\_2526** ( Putative gene predicted by FgenesB );  **C1E\_2527** ( Permeases of the major facilitator superfamily COG0477 Permeases of the major facilitator superfamily );  **C1E\_2528** ( Permeases of the major facilitator superfamily COG0477 Permeases of the major facilitator superfamily );  **C1E\_2529** ( NAD-dependent aldehyde dehydrogenases COG1012 NAD-dependent aldehyde dehydrogenases );  **C1E\_2530** ( Zn-dependent alcohol dehydrogenases, class III COG1062 Zn-dependent alcohol dehydrogenases, class III );  **C1E\_2531** ( Putative gene predicted by FgenesB );  **C1E\_2532** ( gi|197787299|ref|YP\_002209967.1| conserved hypothetical protein );  **C1E\_2533** ( gi|146276858|ref|YP\_001167017.1| hypothetical protein Rsph17025\_0806 );  **C1E\_2534** ( gi|146276859|ref|YP\_001167018.1| hypothetical protein Rsph17025\_0807 );  **C1E\_2535** ( Putative gene predicted by FgenesB );  **C1E\_2536** ( Putative gene predicted by FgenesB );  **C1E\_2537** ( Predicted hydrolases or acyltransferases (alpha/beta hydrolase superfamily) COG0596 Predicted hydrolases or acyltransferases (alpha/beta hydrolase superfamily) );  **C1E\_2538** ( Short-chain dehydrogenases of various substrate specificities COG0300 Short-chain dehydrogenases of various substrate specificities );  **C1E\_2539** ( Dehydrogenases with different specificities (related to short-chain alcohol dehydrogenases) COG1028 Dehydrogenases with different specificities (related to short-chain alcohol dehydrogenases) );  **C1E\_2540** ( gi|188580487|ref|YP\_001923932.1| short-chain dehydrogenase/reductase SDR );  **C1E\_2541** ( gi|196238562|ref|ZP\_03137337.1| short-chain dehydrogenase/reductase SDR );  **C1E\_2542** ( gi|115375402|ref|ZP\_01462664.1| dehydrogenase DhgA );  **C1E\_2543** ( Putative gene predicted by FgenesB );  **C1E\_2544** ( Predicted hydrolase (HAD superfamily) COG1011 Predicted hydrolase (HAD superfamily) ); |
| 2 | 21788 | 554:288778..310565 | **C1E\_1572** ( Integrase COG0582 Integrase );  **C1E\_1573** ( Integrase COG0582 Integrase );  **C1E\_1574** ( Uncharacterized protein conserved in bacteria COG4688 Uncharacterized protein conserved in bacteria );  **C1E\_1575** ( gi|77166473|ref|YP\_344998.1| hypothetical protein Noc\_3026 );  **C1E\_1576** ( Putative gene predicted by FgenesB );  **C1E\_1577** ( gi|59713723|ref|YP\_206498.1| hypothetical protein VF\_A0540 );  **C1E\_1578** ( gi|186471663|ref|YP\_001862981.1| hypothetical protein Bphy\_6925 );  **C1E\_1579** ( gi|109899890|ref|YP\_663145.1| hypothetical protein Patl\_3589 );  **C1E\_1580** ( Predicted P-loop ATPase COG4928 Predicted P-loop ATPase );  **C1E\_1581** ( gi|26250335|ref|NP\_756375.1| hypothetical protein c4513 );  **C1E\_1582** ( gi|169796893|ref|YP\_001714686.1| hypothetical protein ABAYE2885 );  **C1E\_1583** ( Uncharacterized conserved protein COG1479 Uncharacterized conserved protein );  **C1E\_1584** ( gi|200387411|ref|ZP\_03214023.1| hypothetical protein SeV\_B2346 );  **C1E\_1585** ( Putative gene predicted by FgenesB );  **C1E\_1586** ( Putative gene predicted by FgenesB );  **C1E\_1587** ( Putative gene predicted by FgenesB );  **C1E\_1588** ( Putative gene predicted by FgenesB ); |
| 3 | 18740 | 195:62431..81170 | **C1E\_0658** ( gi|121583152|ref|YP\_973593.1| hypothetical protein Pnap\_4583 );  **C1E\_0659** ( gi|121583153|ref|YP\_973594.1| putative ATP-binding protein );  **C1E\_0660** ( gi|121583154|ref|YP\_973595.1| phosphoadenosine phosphosulfate reductase );  **C1E\_0661** ( gi|32471645|ref|NP\_864638.1| serine/threonine protein kinase );  **C1E\_0662** ( Uncharacterized conserved protein COG1479 Uncharacterized conserved protein );  **C1E\_0663** ( DNA segregation ATPase FtsK/SpoIIIE and related proteins COG1674 DNA segregation ATPase FtsK/SpoIIIE and related proteins );  **C1E\_0664** ( gi|77957027|ref|ZP\_00821095.1| hypothetical protein YberA\_01002741 );  **C1E\_0665** ( gi|124262720|ref|YP\_001023190.1| hypothetical protein Mpe\_B0179 );  **C1E\_0666** ( gi|78189815|ref|YP\_380153.1| hypothetical protein Cag\_1860 ); |
| 4 | 17877 | 1053:115552..133428 | **C1E\_4564** ( Putative gene predicted by FgenesB );  **C1E\_4565** ( Putative gene predicted by FgenesB );  **C1E\_4566** ( Putative gene predicted by FgenesB );  **C1E\_4567** ( gi|94311109|ref|YP\_584319.1| hypothetical protein Rmet\_2171 );  **C1E\_4568** ( gi|119946224|ref|YP\_943904.1| phage integrase family protein );  **C1E\_4569** ( gi|16763096|ref|NP\_458713.1| hypothetical protein STY4631 );  **C1E\_4570** ( gi|56414689|ref|YP\_151764.1| hypothetical protein SPA2585 );  **C1E\_4571** ( Putative gene predicted by FgenesB );  **C1E\_4572** ( Superfamily I DNA and RNA helicases COG0210 Superfamily I DNA and RNA helicases );  **C1E\_4573** ( gi|26991154|ref|NP\_746579.1| Cro/CI family transcriptional regulator );  **C1E\_4574** ( Putative gene predicted by FgenesB );  **C1E\_4575** ( Putative gene predicted by FgenesB );  **C1E\_4576** ( gi|213970986|ref|ZP\_03399107.1| ATP-dependent DNA helicase, UvrD/Rep family );  **C1E\_4577** ( Putative gene predicted by FgenesB );  **C1E\_4578** ( gi|21244022|ref|NP\_643604.1| hypothetical protein XAC3296 ); |
| 5 | 16579 | 419:816..17394 | **C1E\_1011** ( gi|29123572|gb|AAO63154.1| STY4579 );  **C1E\_1012** ( gi|68637929|emb|CAI36134.1| hypothetical protein );  **C1E\_1013** ( Permeases of the major facilitator superfamily COG0477 Permeases of the major facilitator superfamily );  **C1E\_1014** ( Biotin carboxylase COG0439 Biotin carboxylase );  **C1E\_1015** ( gi|30060191|gb|AAP13070.1| putative membrane protein );  **C1E\_1016** ( gi|30060191|gb|AAP13070.1| putative membrane protein );  **C1E\_1017** ( Choline dehydrogenase and related flavoproteins COG2303 Choline dehydrogenase and related flavoproteins );  **C1E\_1018** ( Probable taurine catabolism dioxygenase COG2175 Probable taurine catabolism dioxygenase );  **C1E\_1019** ( Asparagine synthase (glutamine-hydrolyzing) COG0367 Asparagine synthase (glutamine-hydrolyzing) );  **C1E\_1020** ( Putative gene predicted by FgenesB );  **C1E\_1021** ( gi|21693557|gb|AAM75344.1|AF519896\_1 tabtoxin biosynthetic region hypothetical protein );  **C1E\_1022** ( gi|21693557|gb|AAM75344.1|AF519896\_1 tabtoxin biosynthetic region hypothetical protein );  **C1E\_1023** ( gi|2506922|sp|P31850|TBLA\_PSESZ Tabtoxin biosynthesis enzyme tabtoxin biosynthesis enzyme TblA );  **C1E\_1024** ( Diaminopimelate decarboxylase COG0019 Diaminopimelate decarboxylase );  **C1E\_1025** ( Tetrahydrodipicolinate N-succinyltransferase COG2171 Tetrahydrodipicolinate N-succinyltransferase );  **C1E\_1026** ( Aspartate/tyrosine/aromatic aminotransferase COG0436 Aspartate/tyrosine/aromatic aminotransferase );  **C1E\_1027** ( Metal-dependent amidase/aminoacylase/carboxypeptidase COG1473 Metal-dependent amidase/aminoacylase/carboxypeptidase ); |
| 6 | 13414 | 1053:372540..385953 | **C1E\_4810** ( Small-conductance mechanosensitive channel COG0668 Small-conductance mechanosensitive channel );  **C1E\_4811** ( gi|213969788|ref|ZP\_03397923.1| DNA-binding protein );  **C1E\_4812** ( gi|26990418|ref|NP\_745843.1| hypothetical protein PP\_3710 );  **C1E\_4813** ( gi|213969790|ref|ZP\_03397925.1| hypothetical protein PSPTOT1\_1216 );  **C1E\_4814** ( gi|213969791|ref|ZP\_03397926.1| hypothetical protein PSPTOT1\_1217 );  **C1E\_4815** ( Putative gene predicted by FgenesB );  **C1E\_4816** ( gi|121611799|ref|YP\_999606.1| hypothetical protein Veis\_4900 );  **C1E\_4817** ( Putative gene predicted by FgenesB );  **C1E\_4818** ( gi|177666441|ref|ZP\_02942236.1| hypothetical protein PCC8801DRAFT\_3119 );  **C1E\_4819** ( Putative gene predicted by FgenesB );  **C1E\_4820** ( gi|187930242|ref|YP\_001900729.1| SMC domain protein );  **C1E\_4821** ( Superfamily I DNA and RNA helicases COG0210 Superfamily I DNA and RNA helicases );  **C1E\_4822** ( gi|149910982|ref|ZP\_01899612.1| hypothetical protein PE36\_08111 );  **C1E\_4823** ( gi|77462592|ref|YP\_352096.1| ATPase );  **C1E\_4824** ( gi|38201477|emb|CAD98777.1| H-NS ); |
| 7 | 13318 | 1053:418757..432074 | **C1E\_4858** ( gi|148550222|ref|YP\_001270324.1| hypothetical protein Pput\_5020 );  **C1E\_4859** ( gi|213970971|ref|ZP\_03399092.1| hypothetical protein PSPTOT1\_4213 );  **C1E\_4860** ( gi|213971694|ref|ZP\_03399801.1| hypothetical protein PSPTOT1\_5428 );  **C1E\_4861** ( gi|157962451|ref|YP\_001502485.1| hypothetical protein Spea\_2630 );  **C1E\_4862** ( gi|213970989|ref|ZP\_03399110.1| hypothetical protein PSPTOT1\_4231 );  **C1E\_4863** ( Putative gene predicted by FgenesB );  **C1E\_4864** ( Putative gene predicted by FgenesB );  **C1E\_4865** ( gi|17975120|ref|NP\_536642.1| hypothetical protein K139p15 );  **C1E\_4866** ( Putative gene predicted by FgenesB );  **C1E\_4867** ( Putative gene predicted by FgenesB );  **C1E\_4868** ( gi|148807440|gb|ABR13512.1| site-specific recombinase );  **C1E\_4869** ( gi|213970991|ref|ZP\_03399112.1| hypothetical protein PSPTOT1\_4233 );  **C1E\_4870** ( gi|187921123|ref|YP\_001890155.1| integrase family protein );  **C1E\_4871** ( Small-conductance mechanosensitive channel COG0668 Small-conductance mechanosensitive channel ); |
| 8 | 12005 | 1053:142165..154169 | **C1E\_4592** ( Putative gene predicted by FgenesB );  **C1E\_4593** ( Uncharacterized protein conserved in bacteria COG3124 Uncharacterized protein conserved in bacteria );  **C1E\_4594** ( Putative gene predicted by FgenesB );  **C1E\_4595** ( Uncharacterized protein conserved in bacteria COG4694 Uncharacterized protein conserved in bacteria );  **C1E\_4596** ( gi|186474382|ref|YP\_001863353.1| FRG domain-containing protein );  **C1E\_4597** ( Putative gene predicted by FgenesB );  **C1E\_4598** ( Putative gene predicted by FgenesB );  **C1E\_4599** ( gi|34496298|ref|NP\_900513.1| hypothetical protein CV\_0843 );  **C1E\_4600** ( Putative gene predicted by FgenesB );  **C1E\_4601** ( Putative gene predicted by FgenesB );  **C1E\_4602** ( gi|213970594|ref|ZP\_03398720.1| hypothetical protein PSPTOT1\_2837 );  **C1E\_4603** ( gi|88799073|ref|ZP\_01114654.1| hypothetical protein MED297\_02702 );  **C1E\_4604** ( Transcriptional regulator COG1309 Transcriptional regulator ); |
| 9 | 11214 | 122:70532..81745 | **C1E\_0354** ( Putative gene predicted by FgenesB );  **C1E\_0355** ( Glycosyltransferases involved in cell wall biogenesis COG0463 Glycosyltransferases involved in cell wall biogenesis );  **C1E\_0356** ( gi|194290471|ref|YP\_002006378.1| hypothetical protein RALTA\_A2383 );  **C1E\_0357** ( Glycosyltransferases involved in cell wall biogenesis COG0463 Glycosyltransferases involved in cell wall biogenesis );  **C1E\_0358** ( ABC-type polysaccharide/polyol phosphate export systems, permease component COG1682 ABC-type polysaccharide/polyol phosphate export systems, permease component );  **C1E\_0359** ( ABC-type polysaccharide/polyol phosphate transport system, ATPase component COG1134 ABC-type polysaccharide/polyol phosphate transport system, ATPase component );  **C1E\_0360** ( Lipopolysaccharide biosynthesis protein COG3754 Lipopolysaccharide biosynthesis protein ); |
| 10 | 10716 | 1053:386294..397009 | **C1E\_4824** ( gi|38201477|emb|CAD98777.1| H-NS );  **C1E\_4825** ( Putative gene predicted by FgenesB );  **C1E\_4826** ( Putative gene predicted by FgenesB );  **C1E\_4827** ( gi|213970986|ref|ZP\_03399107.1| ATP-dependent DNA helicase, UvrD/Rep family );  **C1E\_4828** ( gi|213970987|ref|ZP\_03399108.1| hypothetical protein PSPTOT1\_4229 );  **C1E\_4829** ( gi|213970982|ref|ZP\_03399103.1| hypothetical protein PSPTOT1\_4224 );  **C1E\_4830** ( Putative gene predicted by FgenesB );  **C1E\_4831** ( gi|66047874|ref|YP\_237715.1| hypothetical protein Psyr\_4647 );  **C1E\_4832** ( Putative gene predicted by FgenesB );  **C1E\_4833** ( Putative gene predicted by FgenesB );  **C1E\_4834** ( gi|67153547|ref|ZP\_00415292.1| hypothetical protein AvinDRAFT\_5818 );  **C1E\_4835** ( gi|71907006|ref|YP\_284593.1| hypothetical protein Daro\_1374 );  **C1E\_4836** ( gi|26991106|ref|NP\_746531.1| hypothetical protein PP\_4418 );  **C1E\_4837** ( gi|66045886|ref|YP\_235727.1| hypothetical protein Psyr\_2650 ); |
| 11 | 9743 | 684:153693..163435 | **C1E\_2546** ( Predicted oxidoreductases (related to aryl-alcohol dehydrogenases) COG0667 Predicted oxidoreductases (related to aryl-alcohol dehydrogenases) );  **C1E\_2547** ( Aldo/keto reductases, related to diketogulonate reductase COG0656 Aldo/keto reductases, related to diketogulonate reductase );  **C1E\_2548** ( Arabinose efflux permease COG2814 Arabinose efflux permease );  **C1E\_2549** ( Dehydrogenases with different specificities (related to short-chain alcohol dehydrogenases) COG1028 Dehydrogenases with different specificities (related to short-chain alcohol dehydrogenases) );  **C1E\_2550** ( Predicted phosphohydrolases COG1408 Predicted phosphohydrolases );  **C1E\_2551** ( Transcriptional regulator COG0583 Transcriptional regulator );  **C1E\_2552** ( Predicted hydrolases or acyltransferases (alpha/beta hydrolase superfamily) COG0596 Predicted hydrolases or acyltransferases (alpha/beta hydrolase superfamily) );  **C1E\_2553** ( Gluconolactonase COG3386 Gluconolactonase );  **C1E\_2554** ( Cation/multidrug efflux pump COG0841 Cation/multidrug efflux pump ); |
| 12 | 9482 | 1053:362542..372023 | **C1E\_4801** ( gi|116006770|ref|YP\_787954.1| hypothetical protein pBP136\_p34 );  **C1E\_4802** ( Thermostable 8-oxoguanine DNA glycosylase COG1059 Thermostable 8-oxoguanine DNA glycosylase );  **C1E\_4803** ( Putative gene predicted by FgenesB );  **C1E\_4804** ( Predicted PP-loop superfamily ATPase COG0603 Predicted PP-loop superfamily ATPase );  **C1E\_4805** ( Sugar kinases, ribokinase family COG0524 Sugar kinases, ribokinase family );  **C1E\_4806** ( Putative gene predicted by FgenesB );  **C1E\_4807** ( gi|167361405|ref|ZP\_02295988.1| conserved hypothetical protein );  **C1E\_4808** ( gi|169344607|ref|ZP\_02865573.1| hypothetical protein CPC\_A0224 );  **C1E\_4809** ( Restriction endonuclease COG1403 Restriction endonuclease );  **C1E\_4810** ( Small-conductance mechanosensitive channel COG0668 Small-conductance mechanosensitive channel ); |
| 13 | 9120 | 684:163538..172657 | **C1E\_2554** ( Cation/multidrug efflux pump COG0841 Cation/multidrug efflux pump );  **C1E\_2555** ( Membrane-fusion protein COG0845 Membrane-fusion protein );  **C1E\_2556** ( gi|116625141|ref|YP\_827297.1| ASPIC/UnbV domain-containing protein );  **C1E\_2557** ( Transcriptional regulator COG0583 Transcriptional regulator );  **C1E\_2558** ( Arabinose efflux permease COG2814 Arabinose efflux permease );  **C1E\_2559** ( Uncharacterized conserved protein COG1430 Uncharacterized conserved protein );  **C1E\_2560** ( Glucose/sorbosone dehydrogenases COG2133 Glucose/sorbosone dehydrogenases );  **C1E\_2561** ( gi|26990408|ref|NP\_745833.1| hypothetical protein PP\_3700 ); |
| 14 | 9041 | 98:77035..86075 | **C1E\_0108** ( Branched-chain amino acid aminotransferase/4-amino-4-deoxychorismate lyase COG0115 Branched-chain amino acid aminotransferase/4-amino-4-deoxychorismate lyase );  **C1E\_0109** ( Putative gene predicted by FgenesB );  **C1E\_0110** ( gi|111021415|ref|YP\_704387.1| hypothetical protein RHA1\_ro04443 );  **C1E\_0111** ( ABC-type transport system involved in cytochrome bd biosynthesis, fused ATPase and permease components COG4987 ABC-type transport system involved in cytochrome bd biosynthesis, fused ATPase and permease components );  **C1E\_0112** ( gi|111021417|ref|YP\_704389.1| hypothetical protein RHA1\_ro04445 );  **C1E\_0113** ( Biotin carboxylase COG0439 Biotin carboxylase );  **C1E\_0114** ( Putative gene predicted by FgenesB ); |
| 15 | 8834 | 684:317133..325966 | **C1E\_2711** ( Putative gene predicted by FgenesB );  **C1E\_2712** ( gi|186473776|ref|YP\_001861118.1| hypothetical protein Bphy\_4982 );  **C1E\_2713** ( gi|167456887|ref|ZP\_02323103.1| hypothetical protein A2cp1DRAFT\_1523 );  **C1E\_2714** ( Putative gene predicted by FgenesB );  **C1E\_2715** ( gi|83746582|ref|ZP\_00943632.1| Hypothetical Protein RRSL\_03756 ); |
| 16 | 8638 | 1053:106796..115433 | **C1E\_4558** ( Putative gene predicted by FgenesB );  **C1E\_4559** ( gi|21242950|ref|NP\_642532.1| hypothetical protein XAC2215 );  **C1E\_4560** ( Putative gene predicted by FgenesB );  **C1E\_4561** ( gi|21242949|ref|NP\_642531.1| hypothetical protein XAC2214 );  **C1E\_4562** ( gi|197123729|ref|YP\_002135680.1| hypothetical protein AnaeK\_3335 );  **C1E\_4563** ( gi|190895190|ref|YP\_001985483.1| hypothetical protein RHECIAT\_PC0000863 );  **C1E\_4564** ( Putative gene predicted by FgenesB ); |
| 17 | 8090 | 684:179864..187953 | **C1E\_2571** ( NADH:flavin oxidoreductases, Old Yellow Enzyme family COG1902 NADH:flavin oxidoreductases, Old Yellow Enzyme family );  **C1E\_2572** ( Transcriptional regulator COG1309 Transcriptional regulator );  **C1E\_2573** ( gi|82617198|emb|CAI64105.1| hypothetical protein );  **C1E\_2574** ( gi|91200149|emb|CAJ73193.1| hypothetical protein );  **C1E\_2575** ( Putative gene predicted by FgenesB );  **C1E\_2576** ( Putative gene predicted by FgenesB );  **C1E\_2577** ( Putative gene predicted by FgenesB );  **C1E\_2578** ( Putative gene predicted by FgenesB ); |
| 18 | 7402 | 679:22149..29550 | **C1E\_2250** ( gi|77459725|ref|YP\_349232.1| hypothetical protein Pfl01\_3503 );  **C1E\_2251** ( Site-specific recombinases, DNA invertase Pin homologs COG1961 Site-specific recombinases, DNA invertase Pin homologs );  **C1E\_2252** ( Putative gene predicted by FgenesB );  **C1E\_2253** ( Putative gene predicted by FgenesB );  **C1E\_2254** ( Putative gene predicted by FgenesB );  **C1E\_2255** ( Putative gene predicted by FgenesB );  **C1E\_2256** ( Putative gene predicted by FgenesB );  **C1E\_2257** ( Uncharacterized conserved protein COG4983 Uncharacterized conserved protein );  **C1E\_2258** ( Putative gene predicted by FgenesB );  **C1E\_2259** ( Putative gene predicted by FgenesB );  **C1E\_2260** ( gi|71908115|ref|YP\_285702.1| Phage integrase ); |
| 19 | 7034 | 1053:404811..411844 | **C1E\_4848** ( Putative gene predicted by FgenesB );  **C1E\_4849** ( Restriction endonuclease COG1403 Restriction endonuclease );  **C1E\_4850** ( gi|134288364|ref|YP\_001110527.1| hypothetical protein Bcep1808\_6837 );  **C1E\_4851** ( gi|148977726|ref|ZP\_01814287.1| hypothetical purine NTPase );  **C1E\_4852** ( gi|98978971|gb|ABF59983.1| hypothetical protein QG7\_0013 );  **C1E\_4853** ( gi|98978972|gb|ABF59984.1| conserved hypothetical protein ); |
| 20 | 6591 | 1053:398133..404723 | **C1E\_4838** ( gi|26990414|ref|NP\_745839.1| hypothetical protein PP\_3706 );  **C1E\_4839** ( gi|66045888|ref|YP\_235729.1| hypothetical protein Psyr\_2652 );  **C1E\_4840** ( gi|146281035|ref|YP\_001171188.1| phage Hau3 resistance protein );  **C1E\_4841** ( gi|146281036|ref|YP\_001171189.1| EA31 protein );  **C1E\_4842** ( Putative gene predicted by FgenesB );  **C1E\_4843** ( gi|191164129|ref|ZP\_03025998.1| hypothetical protein GM21DRAFT\_3410 );  **C1E\_4844** ( gi|213970972|ref|ZP\_03399093.1| hypothetical protein PSPTOT1\_4214 );  **C1E\_4845** ( gi|213970986|ref|ZP\_03399107.1| ATP-dependent DNA helicase, UvrD/Rep family );  **C1E\_4846** ( gi|28871871|ref|NP\_794490.1| hypothetical protein PSPTO\_4755 );  **C1E\_4847** ( Fructose-2,6-bisphosphatase COG0406 Fructose-2,6-bisphosphatase ); |
| 21 | 6050 | 1102:92558..98607 | **C1E\_5711** ( Permeases of the major facilitator superfamily COG0477 Permeases of the major facilitator superfamily );  **C1E\_5712** ( Demethylmenaquinone methyltransferase COG0684 Demethylmenaquinone methyltransferase );  **C1E\_5713** ( Phosphoglycerate dehydrogenase and related dehydrogenases COG0111 Phosphoglycerate dehydrogenase and related dehydrogenases );  **C1E\_5714** ( Permeases of the major facilitator superfamily COG0477 Permeases of the major facilitator superfamily );  **C1E\_5715** ( Transcriptional regulator COG1414 Transcriptional regulator ); |
| 22 | 6003 | 684:102632..108634 | **C1E\_2499** ( Putative gene predicted by FgenesB );  **C1E\_2500** ( Putative gene predicted by FgenesB );  **C1E\_2501** ( Transcriptional regulators containing a DNA-binding HTH domain and an aminotransferase domain (MocR family) and their eukaryotic orthologs COG1167 Transcriptional regulators containing a DNA-binding HTH domain and an aminotransferase domain (MocR family) and their eukaryotic orthologs );  **C1E\_2502** ( Histone acetyltransferase HPA2 and related acetyltransferases COG0454 Histone acetyltransferase HPA2 and related acetyltransferases );  **C1E\_2503** ( gi|163754668|ref|ZP\_02161790.1| hypothetical protein KAOT1\_17273 );  **C1E\_2504** ( gi|37528687|ref|NP\_932032.1| hypothetical protein plu4879 ); |
| 23 | 5984 | 684:173704..179687 | **C1E\_2562** ( gi|66047874|ref|YP\_237715.1| hypothetical protein Psyr\_4647 );  **C1E\_2563** ( Predicted transcriptional regulators COG1396 Predicted transcriptional regulators );  **C1E\_2564** ( gi|28867267|ref|NP\_789886.1| HAD superfamily hydrolase );  **C1E\_2565** ( Putative gene predicted by FgenesB );  **C1E\_2566** ( Putative gene predicted by FgenesB );  **C1E\_2567** ( Putative gene predicted by FgenesB );  **C1E\_2568** ( Putative gene predicted by FgenesB );  **C1E\_2569** ( gi|167035796|ref|YP\_001671027.1| metallophosphoesterase );  **C1E\_2570** ( gi|26991154|ref|NP\_746579.1| Cro/CI family transcriptional regulator );  **C1E\_2571** ( NADH:flavin oxidoreductases, Old Yellow Enzyme family COG1902 NADH:flavin oxidoreductases, Old Yellow Enzyme family ); |
| 24 | 5234 | 256:36770..42003 | **C1E\_0896** ( ATPases of the AAA+ class COG0464 ATPases of the AAA+ class );  **C1E\_0897** ( Subtilisin-like serine proteases COG1404 Subtilisin-like serine proteases );  **C1E\_0898** ( gi|149908533|ref|ZP\_01897195.1| hypothetical protein PE36\_15944 );  **C1E\_0899** ( Putative gene predicted by FgenesB ); |
| 25 | 4989 | 122:85820..90808 | **C1E\_0363** ( GDP-D-mannose dehydratase COG1089 GDP-D-mannose dehydratase );  **C1E\_0364** ( Uncharacterized protein conserved in bacteria COG4529 Uncharacterized protein conserved in bacteria );  **C1E\_0365** ( Adenylylsulfate kinase and related kinases COG0529 Adenylylsulfate kinase and related kinases );  **C1E\_0366** ( Predicted acyltransferases COG1835 Predicted acyltransferases );  **C1E\_0367** ( Mannose-1-phosphate guanylyltransferase COG0836 Mannose-1-phosphate guanylyltransferase ); |
| 26 | 4795 | 955:35644..40438 | **C1E\_3923** ( Putative copper export protein COG1276 Putative copper export protein );  **C1E\_3924** ( Predicted metal-binding protein COG3019 Predicted metal-binding protein );  **C1E\_3925** ( gi|146307237|ref|YP\_001187702.1| outer membrane efflux protein );  **C1E\_3926** ( Membrane-fusion protein COG0845 Membrane-fusion protein );  **C1E\_3927** ( Putative silver efflux pump COG3696 Putative silver efflux pump ); |
| 27 | 4399 | 1160:380000..384398 | **C1E\_6105** ( gi|120556622|ref|YP\_960973.1| hypothetical protein Maqu\_3717 );  **C1E\_6106** ( gi|120556230|ref|YP\_960581.1| hypothetical protein Maqu\_3323 );  **C1E\_6107** ( gi|28868007|ref|NP\_790626.1| hypothetical protein PSPTO\_0779 ); |
| 28 | 4345 | 1053:411929..416273 | **C1E\_4854** ( Putative gene predicted by FgenesB );  **C1E\_4855** ( gi|168136318|ref|ZP\_02579547.1| hypothetical protein BcerB\_15332 );  **C1E\_4856** ( gi|26990401|ref|NP\_745826.1| transcriptional regulator MvaT, P16 subunit, putative ); |
| 29 | 4162 | 684:188035..192196 | **C1E\_2579** ( Putative gene predicted by FgenesB );  **C1E\_2580** ( Transposase and inactivated derivatives COG2801 Transposase and inactivated derivatives );  **C1E\_2581** ( Putative gene predicted by FgenesB );  **C1E\_2582** ( Putative gene predicted by FgenesB );  **C1E\_2583** ( Methyl-accepting chemotaxis protein COG0840 Methyl-accepting chemotaxis protein ); |
| 30 | 4151 | 256:42209..46359 | **C1E\_0900** ( gi|157411390|gb|ABV54348.1| hypothetical protein );  **C1E\_0901** ( Transcriptional regulator COG1309 Transcriptional regulator );  **C1E\_0902** ( gi|114328442|ref|YP\_745599.1| putative cytoplasmic protein );  **C1E\_0903** ( Predicted hydrolases or acyltransferases (alpha/beta hydrolase superfamily) COG0596 Predicted hydrolases or acyltransferases (alpha/beta hydrolase superfamily) ); |
| 31 | 3720 | 684:194978..198697 | **C1E\_2587** ( Methyl-accepting chemotaxis protein COG0840 Methyl-accepting chemotaxis protein );  **C1E\_2588** ( gi|213972096|ref|ZP\_03400188.1| hypothetical protein PSPTOT1\_2454 );  **C1E\_2589** ( Dienelactone hydrolase and related enzymes COG0412 Dienelactone hydrolase and related enzymes );  **C1E\_2590** ( Putative gene predicted by FgenesB ); |
| 32 | 3537 | 733:69542..73078 | **C1E\_2858** ( gi|71737532|ref|YP\_274516.1| insecticidal toxin complex protein, putative ); |
| 33 | 3262 | 1039:129449..132710 | **C1E\_4255** ( Putative gene predicted by FgenesB );  **C1E\_4256** ( Putative gene predicted by FgenesB ); |
| 34 | 3134 | 832:327515..330648 | **C1E\_3210** ( Permeases of the major facilitator superfamily COG0477 Permeases of the major facilitator superfamily );  **C1E\_3211** ( Dihydrodipicolinate synthase/N-acetylneuraminate lyase COG0329 Dihydrodipicolinate synthase/N-acetylneuraminate lyase );  **C1E\_3212** ( gi|187927663|ref|YP\_001898150.1| dihydrodipicolinate synthase );  **C1E\_3213** ( Alcohol dehydrogenase, class IV COG1454 Alcohol dehydrogenase, class IV );  **C1E\_3214** ( Uncharacterized protein conserved in bacteria COG3395 Uncharacterized protein conserved in bacteria ); |
| 35 | 3127 | 1053:358176..361302 | **C1E\_4794** ( gi|153000097|ref|YP\_001365778.1| hypothetical protein Shew185\_1568 );  **C1E\_4795** ( gi|153000096|ref|YP\_001365777.1| AAA ATPase );  **C1E\_4796** ( gi|153000096|ref|YP\_001365777.1| AAA ATPase );  **C1E\_4797** ( gi|70729403|ref|YP\_259141.1| ultraviolet light resistance protein B, interruption-C ); |
| 36 | 3123 | 98:41278..44400 | **C1E\_0063** ( Rhs family protein COG3209 Rhs family protein );  **C1E\_0064** ( gi|85702814|ref|ZP\_01033918.1| hypothetical protein ROS217\_18772 );  **C1E\_0065** ( Putative gene predicted by FgenesB );  **C1E\_0066** ( Rhs family protein COG3209 Rhs family protein ); |
| 37 | 3067 | 1160:361866..364932 | **C1E\_6087** ( Uncharacterized protein conserved in bacteria COG3515 Uncharacterized protein conserved in bacteria );  **C1E\_6088** ( Hemolysin-coregulated protein (uncharacterized) COG3157 Hemolysin-coregulated protein (uncharacterized) );  **C1E\_6089** ( Uncharacterized protein conserved in bacteria COG3501 Uncharacterized protein conserved in bacteria );  **C1E\_6090** ( Predicted permeases COG0730 Predicted permeases ); |
| 38 | 3061 | 1160:321986..325046 | **C1E\_6050** ( Aspartate/tyrosine/aromatic aminotransferase COG0436 Aspartate/tyrosine/aromatic aminotransferase );  **C1E\_6051** ( FAD/FMN-containing dehydrogenases COG0277 FAD/FMN-containing dehydrogenases ); |
| 39 | 3034 | 122:81797..84830 | **C1E\_0360** ( Lipopolysaccharide biosynthesis protein COG3754 Lipopolysaccharide biosynthesis protein );  **C1E\_0361** ( Glycosyltransferase COG0438 Glycosyltransferase );  **C1E\_0362** ( Nucleoside-diphosphate-sugar epimerases COG0451 Nucleoside-diphosphate-sugar epimerases );  **C1E\_0363** ( GDP-D-mannose dehydratase COG1089 GDP-D-mannose dehydratase ); |
| 40 | 2837 | 672:99588..102424 | **C1E\_2034** ( Predicted dinucleotide-binding enzymes COG2085 Predicted dinucleotide-binding enzymes );  **C1E\_2035** ( Predicted transcriptional regulators COG1733 Predicted transcriptional regulators );  **C1E\_2036** ( gi|68637852|emb|CAI36057.1| cysteine protease ); |
| 41 | 2833 | 891:140000..142832 | **C1E\_3421** ( gi|5759102|gb|AAD50977.1|AF170066\_1 IS870-like transposase );  **C1E\_3422** ( Putative gene predicted by FgenesB );  **C1E\_3423** ( Large exoproteins involved in heme utilization or adhesion COG3210 Large exoproteins involved in heme utilization or adhesion ); |
| 42 | 2696 | 1053:355080..357775 | **C1E\_4791** ( gi|163854509|ref|YP\_001628807.1| hypothetical protein Bpet0205 );  **C1E\_4792** ( gi|148550219|ref|YP\_001270321.1| hypothetical protein Pput\_5017 ); |
| 43 | 2640 | 122:91046..93685 | **C1E\_0367** ( Mannose-1-phosphate guanylyltransferase COG0836 Mannose-1-phosphate guanylyltransferase );  **C1E\_0368** ( Glycosyltransferases involved in cell wall biogenesis COG0463 Glycosyltransferases involved in cell wall biogenesis ); |
| 44 | 2596 | 679:47843..50438 | **C1E\_2283** ( gi|67158320|ref|ZP\_00419311.1| Hemolysin-type calcium-binding region );  **C1E\_2284** ( RTX toxins and related Ca2+-binding proteins COG2931 RTX toxins and related Ca2+-binding proteins ); |
| 45 | 2424 | 955:53905..56328 | **C1E\_3940** ( Putative gene predicted by FgenesB );  **C1E\_3941** ( gi|42560559|ref|NP\_964009.1| Orf12 );  **C1E\_3942** ( ATPases involved in chromosome partitioning COG1192 ATPases involved in chromosome partitioning );  **C1E\_3943** ( Putative gene predicted by FgenesB );  **C1E\_3944** ( Putative gene predicted by FgenesB );  **C1E\_3945** ( gi|38257042|ref|NP\_940696.1| Orf10 ); |
| 46 | 2391 | 1087:20290..22680 | **C1E\_4932** ( gi|71737381|ref|YP\_273046.1| type IV pilus protein PilM, putative );  **C1E\_4933** ( Site-specific recombinases, DNA invertase Pin homologs COG1961 Site-specific recombinases, DNA invertase Pin homologs );  **C1E\_4934** ( Putative gene predicted by FgenesB );  **C1E\_4935** ( Putative gene predicted by FgenesB );  **C1E\_4936** ( gi|194555969|ref|YP\_002090996.1| hypothetical protein PA2G\_05863 ); |
| 47 | 2380 | 1087:461677..464056 | **C1E\_5458** ( gi|77456375|ref|YP\_345880.1| peptidase M50 );  **C1E\_5459** ( Histone acetyltransferase HPA2 and related acetyltransferases COG0454 Histone acetyltransferase HPA2 and related acetyltransferases );  **C1E\_5460** ( gi|77456382|ref|YP\_345887.1| hypothetical protein Pfl01\_0154 );  **C1E\_5461** ( Microcystin-dependent protein COG4675 Microcystin-dependent protein );  **C1E\_5462** ( gi|77456385|ref|YP\_345890.1| sulfotransferase ); |
| 48 | 2277 | 955:75119..77395 | **C1E\_3964** ( gi|49188583|ref|YP\_025680.1| type III secreted effector hopPmaA ); |
| 49 | 2218 | 955:27047..29264 | **C1E\_3913** ( Spermidine/putrescine-binding periplasmic protein COG0687 Spermidine/putrescine-binding periplasmic protein );  **C1E\_3914** ( DNA-binding HTH domain-containing proteins COG2771 DNA-binding HTH domain-containing proteins );  **C1E\_3915** ( gi|170697494|ref|ZP\_02888585.1| hypothetical protein BamIOP4010DRAFT\_0647 ); |
| 50 | 2155 | 928:22065..24219 | **C1E\_3706** ( gi|126659740|ref|ZP\_01730868.1| hypothetical protein CY0110\_23281 );  **C1E\_3707** ( gi|66043313|ref|YP\_233154.1| putative lipoprotein ); |
| 51 | 2139 | 679:88213..90351 | **C1E\_2321** ( gi|66044048|ref|YP\_233889.1| hypothetical protein Psyr\_0793 );  **C1E\_2322** ( FOG: TPR repeat, SEL1 subfamily COG0790 FOG: TPR repeat, SEL1 subfamily );  **C1E\_2323** ( Putative gene predicted by FgenesB ); |
| 52 | 2110 | 955:182441..184550 | **C1E\_4073** ( Deoxycytidylate deaminase COG2131 Deoxycytidylate deaminase );  **C1E\_4074** ( McrBC 5-methylcytosine restriction system component COG4268 McrBC 5-methylcytosine restriction system component ); |
| 53 | 2070 | 955:66700..68769 | **C1E\_3955** ( gi|49188565|ref|YP\_025663.1| hypothetical protein PMA4326A45 );  **C1E\_3956** ( gi|49188564|ref|YP\_025662.1| hypothetical protein PMA4326A44 );  **C1E\_3957** ( gi|38257083|ref|NP\_940737.1| stability protein ); |
| 54 | 2064 | 1053:416522..418585 | **C1E\_4856** ( gi|26990401|ref|NP\_745826.1| transcriptional regulator MvaT, P16 subunit, putative );  **C1E\_4857** ( Putative gene predicted by FgenesB );  **C1E\_4858** ( gi|148550222|ref|YP\_001270324.1| hypothetical protein Pput\_5020 ); |
| 55 | 2009 | 98:73449..75457 | **C1E\_0104** ( Putative gene predicted by FgenesB );  **C1E\_0105** ( Histone acetyltransferase HPA2 and related acetyltransferases COG0454 Histone acetyltransferase HPA2 and related acetyltransferases ); |
| 56 | 1920 | 1053:349705..351624 | **C1E\_4786** ( FOG: TPR repeat, SEL1 subfamily COG0790 FOG: TPR repeat, SEL1 subfamily );  **C1E\_4787** ( Putative gene predicted by FgenesB );  **C1E\_4788** ( Putative gene predicted by FgenesB ); |
| 57 | 1852 | 955:45094..46945 | **C1E\_3929** ( Cation transport ATPase COG2217 Cation transport ATPase );  **C1E\_3930** ( gi|8388756|dbj|BAA96509.1| ORFH ); |
| 58 | 1833 | 1160:385734..387566 | **C1E\_6109** ( gi|28868007|ref|NP\_790626.1| hypothetical protein PSPTO\_0779 );  **C1E\_6110** ( gi|28868007|ref|NP\_790626.1| hypothetical protein PSPTO\_0779 ); |
| 59 | 1820 | 1087:42958..44777 | **C1E\_4965** ( Putative gene predicted by FgenesB );  **C1E\_4966** ( Putative gene predicted by FgenesB );  **C1E\_4967** ( Putative gene predicted by FgenesB );  **C1E\_4968** ( Putative gene predicted by FgenesB );  **C1E\_4969** ( Putative gene predicted by FgenesB ); |
| 60 | 1782 | 955:64874..66655 | **C1E\_3953** ( ATP-dependent exoDNAse (exonuclease V), alpha subunit - helicase superfamily I member COG0507 ATP-dependent exoDNAse (exonuclease V), alpha subunit - helicase superfamily I member );  **C1E\_3954** ( gi|38257084|ref|NP\_940738.1| Orf53 ); |
| 61 | 1781 | 1160:343716..345496 | **C1E\_6071** ( Histidyl-tRNA synthetase COG0124 Histidyl-tRNA synthetase );  **C1E\_6072** ( Transcriptional regulator COG0583 Transcriptional regulator );  **C1E\_6073** ( NADPH:quinone reductase and related Zn-dependent oxidoreductases COG0604 NADPH:quinone reductase and related Zn-dependent oxidoreductases ); |
| 62 | 1707 | 955:31002..32708 | **C1E\_3917** ( Putative multicopper oxidases COG2132 Putative multicopper oxidases );  **C1E\_3918** ( Uncharacterized protein conserved in bacteria COG3544 Uncharacterized protein conserved in bacteria );  **C1E\_3919** ( Putative gene predicted by FgenesB );  **C1E\_3920** ( Putative multicopper oxidases COG2132 Putative multicopper oxidases ); |
| 63 | 1661 | 672:97788..99448 | **C1E\_2032** ( gi|28872491|ref|NP\_795110.1| phosphoglycerate mutase family protein );  **C1E\_2033** ( Uncharacterized protein conserved in bacteria COG3575 Uncharacterized protein conserved in bacteria ); |
| 64 | 1648 | 98:37102..38749 | **C1E\_0061** ( gi|66045559|ref|YP\_235400.1| YD repeat-containing protein );  **C1E\_0062** ( Rhs family protein COG3209 Rhs family protein ); |
| 65 | 1646 | 1087:465610..467255 | **C1E\_5463** ( gi|119489070|ref|ZP\_01621976.1| hypothetical protein L8106\_22246 );  **C1E\_5464** ( gi|192809377|ref|ZP\_03038052.1| S-layer domain protein );  **C1E\_5465** ( gi|170719861|ref|YP\_001747549.1| Pyrrolo-quinoline quinone ); |
| 66 | 1642 | 955:42375..44016 | **C1E\_3927** ( Putative silver efflux pump COG3696 Putative silver efflux pump );  **C1E\_3928** ( Predicted transcriptional regulators COG0789 Predicted transcriptional regulators );  **C1E\_3929** ( Cation transport ATPase COG2217 Cation transport ATPase ); |
| 67 | 1573 | 684:274547..276119 | **C1E\_2667** ( Putative gene predicted by FgenesB );  **C1E\_2668** ( Putative gene predicted by FgenesB );  **C1E\_2669** ( Putative gene predicted by FgenesB ); |
| 68 | 1545 | 1053:154484..156028 | **C1E\_4605** ( gi|158423769|ref|YP\_001525061.1| hypothetical protein AZC\_2145 ); |
| 69 | 1538 | 955:50786..52323 | **C1E\_3935** ( gi|116622258|ref|YP\_824414.1| hypothetical protein Acid\_3152 );  **C1E\_3936** ( gi|153801432|ref|ZP\_01956018.1| conserved hypothetical protein );  **C1E\_3937** ( Putative gene predicted by FgenesB ); |
| 70 | 1535 | 672:26268..27802 | **C1E\_1962** ( gi|170721709|ref|YP\_001749397.1| hypothetical protein PputW619\_2532 );  **C1E\_1963** ( gi|83749882|ref|ZP\_00946847.1| Core protein );  **C1E\_1964** ( Rhs family protein COG3209 Rhs family protein ); |
| 71 | 1484 | 684:151532..153015 | **C1E\_2544** ( Predicted hydrolase (HAD superfamily) COG1011 Predicted hydrolase (HAD superfamily) );  **C1E\_2545** ( Nucleoside-diphosphate-sugar epimerases COG0451 Nucleoside-diphosphate-sugar epimerases );  **C1E\_2546** ( Predicted oxidoreductases (related to aryl-alcohol dehydrogenases) COG0667 Predicted oxidoreductases (related to aryl-alcohol dehydrogenases) ); |
| 72 | 1448 | 684:101116..102563 | **C1E\_2497** ( Predicted pyridoxal phosphate-dependent enzyme apparently involved in regulation of cell wall biogenesis COG0399 Predicted pyridoxal phosphate-dependent enzyme apparently involved in regulation of cell wall biogenesis );  **C1E\_2498** ( Putative gene predicted by FgenesB ); |
| 73 | 1397 | 554:224745..226141 | **C1E\_1492** ( Bacteriophage tail assembly protein COG5525 Bacteriophage tail assembly protein );  **C1E\_1493** ( gi|213967636|ref|ZP\_03395783.1| tail fiber assembly domain protein ); |
| 74 | 1396 | 684:309841..311236 | **C1E\_2702** ( Putative gene predicted by FgenesB ); |
| 75 | 1345 | 1053:226234..227578 | **C1E\_4671** ( gi|213970832|ref|ZP\_03398955.1| hypothetical protein PSPTOT1\_5671 );  **C1E\_4672** ( gi|66047150|ref|YP\_236991.1| hypothetical protein Psyr\_3923 );  **C1E\_4673** ( Uncharacterized protein conserved in bacteria COG4645 Uncharacterized protein conserved in bacteria ); |
| 76 | 1341 | 891:145252..146592 | **C1E\_3424** ( Large exoproteins involved in heme utilization or adhesion COG3210 Large exoproteins involved in heme utilization or adhesion ); |
| 77 | 1322 | 479:147217..148538 | **C1E\_1185** ( gi|114330127|ref|YP\_746349.1| hypothetical protein Neut\_0096 );  **C1E\_1186** ( gi|71734323|ref|YP\_275644.1| hypothetical protein PSPPH\_3490 ); |
| 78 | 1290 | 1087:28173..29462 | **C1E\_4947** ( Putative gene predicted by FgenesB );  **C1E\_4948** ( Putative gene predicted by FgenesB );  **C1E\_4949** ( Putative gene predicted by FgenesB ); |
| 79 | 1287 | 955:25663..26949 | **C1E\_3911** ( gi|29171513|ref|NP\_808697.1| PbsX family transcriptional regulator );  **C1E\_3912** ( Putative gene predicted by FgenesB );  **C1E\_3913** ( Spermidine/putrescine-binding periplasmic protein COG0687 Spermidine/putrescine-binding periplasmic protein ); |
| 80 | 1269 | 672:41848..43116 | **C1E\_1977** ( gi|28872139|ref|NP\_794758.1| hypothetical protein PSPTO\_5025 );  **C1E\_1978** ( gi|213970755|ref|ZP\_03398879.1| hypothetical protein PSPTOT1\_4095 );  **C1E\_1979** ( gi|213970756|ref|ZP\_03398880.1| hypothetical protein PSPTOT1\_4096 ); |
| 81 | 1257 | 684:192827..194083 | **C1E\_2583** ( Methyl-accepting chemotaxis protein COG0840 Methyl-accepting chemotaxis protein );  **C1E\_2584** ( gi|26989362|ref|NP\_744787.1| methyl-accepting chemotaxis sensory transducer );  **C1E\_2585** ( gi|167034181|ref|YP\_001669412.1| methyl-accepting chemotaxis sensory transducer );  **C1E\_2586** ( gi|71725273|ref|YP\_272259.1| RulB protein ); |
| 82 | 1257 | 928:135997..137253 | **C1E\_3808** ( Protein involved in cell division COG2184 Protein involved in cell division );  **C1E\_3809** ( gi|70728392|ref|YP\_258141.1| hypothetical protein PFL\_1010 );  **C1E\_3810** ( Phosphatidylserine/ phosphatidylglycerophosphate/cardioli pin synthases and related enzymes COG1502 Phosphatidylserine/phosphatidylglycerophosphate/cardioli pin synthases and related enzymes ); |
| 83 | 1227 | 88:956..2182 | **C1E\_0016** ( gi|213969657|ref|ZP\_03397792.1| hypothetical protein PSPTOT1\_1083 ); |
| 84 | 1226 | 955:18553..19778 | **C1E\_3902** ( gi|38257074|ref|NP\_940728.1| VirB6 );  **C1E\_3903** ( Putative gene predicted by FgenesB );  **C1E\_3904** ( gi|49188547|ref|YP\_025645.1| VirB5 ); |
| 85 | 1224 | 1160:350628..351851 | **C1E\_6077** ( Uncharacterized protein conserved in bacteria COG3455 Uncharacterized protein conserved in bacteria );  **C1E\_6078** ( Uncharacterized protein conserved in bacteria COG3522 Uncharacterized protein conserved in bacteria ); |
| 86 | 1207 | 1087:72007..73213 | **C1E\_5008** ( Putative gene predicted by FgenesB );  **C1E\_5009** ( gi|7677395|gb|AAF67149.1|AF231452\_2 ORF2 );  **C1E\_5010** ( gi|55669685|pdb|1S28|A Chain A, Crystal Structure Of Avrpphf Orf1, The Chaperone For The Type Iii Effector Avrpphf Orf2 From P. Syringae Chain B, Crystal Structure Of Avrpphf Orf1, The Chaperone For The Type Iii Effector Avrpphf Orf2 From P. Syringae Chain C, Crystal Structure Of Avrpphf Orf1, The Chaperone For The Type Iii Effector Avrpphf Orf2 From P. Syringae Chain D, Crystal Structure Of Avrpphf Orf1, The Chaperone For The Type Iii Effector Avrpphf Orf2 From P. Syringae );  **C1E\_5011** ( Nucleotidyltransferase/DNA polymerase involved in DNA repair COG0389 Nucleotidyltransferase/DNA polymerase involved in DNA repair ); |
| 87 | 1203 | 672:18724..19926 | **C1E\_1952** ( gi|194565434|ref|YP\_002100456.1| hypothetical protein BDAG\_03748 ); |
| 88 | 1166 | 554:353902..355067 | **C1E\_1636** ( gi|148545935|ref|YP\_001266037.1| hypothetical protein Pput\_0689 );  **C1E\_1637** ( gi|71734479|ref|YP\_276607.1| hypothetical protein PSPPH\_4491 ); |
| 89 | 1141 | 672:22033..23173 | **C1E\_1955** ( gi|170721705|ref|YP\_001749393.1| hypothetical protein PputW619\_2526 );  **C1E\_1956** ( Rhs family protein COG3209 Rhs family protein );  **C1E\_1957** ( Putative gene predicted by FgenesB ); |
| 90 | 1136 | 672:103431..104566 | **C1E\_2038** ( gi|66048145|ref|YP\_237986.1| hypothetical protein Psyr\_4921 );  **C1E\_2039** ( gi|154814556|gb|ABS87317.1| AvrPto1-Cya fusion protein ); |
| 91 | 1126 | 672:118154..119279 | **C1E\_2056** ( Predicted transcriptional regulator COG2944 Predicted transcriptional regulator );  **C1E\_2057** ( Putative gene predicted by FgenesB ); |
| 92 | 1125 | 672:286680..287804 | **C1E\_2218** ( gi|66048030|ref|YP\_237871.1| hypothetical protein Psyr\_4806 );  **C1E\_2219** ( gi|213972294|ref|ZP\_03400359.1| hypothetical protein PSPTOT1\_5602 );  **C1E\_2220** ( Rhs family protein COG3209 Rhs family protein ); |
| 93 | 1112 | 98:71853..72964 | **C1E\_0101** ( gi|213971325|ref|ZP\_03399441.1| hypothetical protein PSPTOT1\_2226 );  **C1E\_0102** ( gi|28872544|ref|NP\_795163.1| Rhs element Vgr protein );  **C1E\_0103** ( gi|28869728|ref|NP\_792347.1| hypothetical protein PSPTO\_2535 ); |
| 94 | 1104 | 672:40470..41573 | **C1E\_1974** ( Putative gene predicted by FgenesB );  **C1E\_1975** ( gi|213970755|ref|ZP\_03398879.1| hypothetical protein PSPTOT1\_4095 );  **C1E\_1976** ( gi|213970756|ref|ZP\_03398880.1| hypothetical protein PSPTOT1\_4096 );  **C1E\_1977** ( gi|28872139|ref|NP\_794758.1| hypothetical protein PSPTO\_5025 ); |
| 95 | 1095 | 1053:984..2078 | **C1E\_4452** ( gi|213966548|ref|ZP\_03394699.1| conserved hypothetical protein );  **C1E\_4453** ( gi|213966547|ref|ZP\_03394698.1| hypothetical protein PSPTOT1\_0305 ); |
| 96 | 1095 | 1087:45382..46476 | **C1E\_4971** ( Putative gene predicted by FgenesB );  **C1E\_4972** ( Putative gene predicted by FgenesB );  **C1E\_4973** ( Putative gene predicted by FgenesB ); |
| 97 | 1068 | 1039:137498..138565 | **C1E\_4263** ( DNA modification methylase COG0863 DNA modification methylase );  **C1E\_4264** ( Putative gene predicted by FgenesB );  **C1E\_4265** ( Putative gene predicted by FgenesB ); |
| 98 | 1016 | 684:377906..378921 | **C1E\_2778** ( Putative gene predicted by FgenesB ); |
| 99 | 1014 | 672:24747..25760 | **C1E\_1960** ( gi|161506541|ref|YP\_001573662.1| hypothetical protein Bmul\_6209 );  **C1E\_1961** ( gi|161506542|ref|YP\_001573663.1| RHS protein ); |
| 100 | 1011 | 554:165631..166641 | **C1E\_1433** ( Peptide methionine sulfoxide reductase COG0225 Peptide methionine sulfoxide reductase );  **C1E\_1434** ( Putative gene predicted by FgenesB );  **C1E\_1435** ( Putative gene predicted by FgenesB ); |
| 101 | 1009 | 1087:329981..330989 | **C1E\_5314** ( Putative gene predicted by FgenesB ); |
| 102 | 1004 | 98:57804..58807 | **C1E\_0083** ( Methyl-accepting chemotaxis protein COG0840 Methyl-accepting chemotaxis protein );  **C1E\_0084** ( Predicted SAM-dependent methyltransferases COG1092 Predicted SAM-dependent methyltransferases ); |


Untitled Document
