# Supplementary material for: A draft genome sequence and functional screen reveals the repertoire of type III secreted proteins of Pseudomonas syringae pathovar tabaci 11528
Source: BMC Genomics. 2009 Aug 24;10:395. doi: 10.1186/1471-2164-10-395 (PMC2745422; doi:10.1186/1471-2164-10-395)
Supplement: Additional file 5 — Table S3. Verification of predicted genes by capillary sequencing. We verified a selection of genes predicted from the Illumina-based Pta11528 genome sequence assembly by capillary sequencing of cloned PCR products. Sequence reads were trimmed to remove poor quality nucleotide calls and the trimmed sequences were aligned against predicted proteins using TBLASTN. [file 1471-2164-10-395-S5.html]

Verification of predicted genes by capillary sequencing


# Verification of predicted genes by capillary sequencing

We verified a selection of genes predicted from the Illumina-based *Pta*11528 genome sequence assembly by capillary sequencing of cloned PCR products. Sequence reads were trimmed to remove poor quality nucleotide calls and the trimmed sequences were aligned against predicted proteins using TBLASTN.

### The predicted proteins that we verified:

**C1E\_0551** 174:125987..126916 DnaJ-class molecular chaperone COG2214

**C1E\_1425** 554:155221..156516 Acetylornithine deacetylase/Succinyl-diaminopimelate desuccinylase and related deacylases Operon 80 Gene 1 COG0624

**C1E\_2036** 672:101352..102155 Cysteine protease

**C1E\_2087** 672:146130..147095 ABC-type nitrate/sulfonate/bicarbonate transport systems, periplasmic components COG0715

**C1E\_3964** 955:74860..77184 Type III secreted effector HopPmaA (HopW1)

**C1E\_3975** 955:85214..86053 Type III effector HopAB2

**C1E\_4987** 1087:57088..58188 N-Dimethylarginine dimethylaminohydrolase COG1834

**C1E\_4990** 1087:59133..60425 Permeases of the major facilitator superfamily

**C1E\_5009** 1087:72050..72664 ORF2

**C1E\_5021** 1087:77437..78576 Type III effector HopT1-1

**C1E\_5022** 1087:78582..79433 Type III effector HopO1-1

**C1E\_5023** 1087:79682..80245 Type III chaperone ShcO1

**C1E\_5300** 1087:315085..316227 AvrPphE

**C1E\_5301** 1087:316323..318641 Type III effector HrpK1

**C1E\_5342** 1087:352578..353465 Type III effector HopAA1-1

**C1E\_5343** 1087:353470..354039 Type III effector HopAA1-2

---

### **C1E\_0551** 174:125987..126916 DnaJ-class molecular chaperone Operon 70 Gene 1 COG2214 DnaJ-class molecular chaperone

|  |  |  |  |
| --- | --- | --- | --- |
| 1e-180 | capillary\_sequence\_23 | ``` C1E_0551 1-309  capillary_sequence_23 106-1032 ``` | ``` MNVVSHIAGKVALQVNSSLLEQKGRLNERQQKGLAVILDALRGKEPVTHVETHEGGGRFNLARAAFDVASVVWERDKSMHNVMSFLGVSDSQGKMLFSLGKKLADAMAKPEPAQGKDNCEAHHAFFSSNLKLNKLMNDIADQLINEIRQSNTDRVRRPTPGPSWRPESAQQQAHPQTPPRTRPQANSTPPPPKAKPNAGAERPSTQRPHNTPAADASAKVSDSAPAKSPVKPLYEHLGLSDMTADLSAVKTAYKKASLKNHPDKNVGNEAEATERFKVISNAFKILSDPELRKKYDNGLINEKGDEVQI MNVVSHIAGKVALQVNSSLLEQKGRLNERQQKGLAVILDALRGKEPVTHVETHEGGGRFNLARAAFDVASVVWERDKSMHNVMSFLGVSDSQGKMLFSLGKKLADAMAKPEPAQGKDNCEAHHAFFSSNLKLNKLMNDIADQLINEIRQSNTDRVRRPTPGPSWRPESAQQQAHPQTPPRTRPQANSTPPPPKAKPNAGAERPSTQRPHNTPAADASAKVSDSAPAKSPVKPLYEHLGLSDMTADLSAVKTAYKKASLKNHPDKNVGNEAEATERFKVISNAFKILSDPELRKKYDNGLINEKGDEVQI MNVVSHIAGKVALQVNSSLLEQKGRLNERQQKGLAVILDALRGKEPVTHVETHEGGGRFNLARAAFDVASVVWERDKSMHNVMSFLGVSDSQGKMLFSLGKKLADAMAKPEPAQGKDNCEAHHAFFSSNLKLNKLMNDIADQLINEIRQSNTDRVRRPTPGPSWRPESAQQQAHPQTPPRTRPQANSTPPPPKAKPNAGAERPSTQRPHNTPAADASAKVSDSAPAKSPVKPLYEHLGLSDMTADLSAVKTAYKKASLKNHPDKNVGNEAEATERFKVISNAFKILSDPELRKKYDNGLINEKGDEVQI ``` |
| 1e-149 | capillary\_sequence\_59 | ``` C1E_0551 1-258  capillary_sequence_59 106-879 ``` | ``` MNVVSHIAGKVALQVNSSLLEQKGRLNERQQKGLAVILDALRGKEPVTHVETHEGGGRFNLARAAFDVASVVWERDKSMHNVMSFLGVSDSQGKMLFSLGKKLADAMAKPEPAQGKDNCEAHHAFFSSNLKLNKLMNDIADQLINEIRQSNTDRVRRPTPGPSWRPESAQQQAHPQTPPRTRPQANSTPPPPKAKPNAGAERPSTQRPHNTPAADASAKVSDSAPAKSPVKPLYEHLGLSDMTADLSAVKTAYKKASL MNVVSHIAGKVALQVNSSLLEQKGRLNERQQKGLAVILDALRGKEPVTHVETHEGGGRFNLARAAFDVASVVWERDKSMHNVMSFLGVSDSQGKMLFSLGKKLADAMAKPEPAQGKDNCEAHHAFFSSNLKLNKLMNDIADQLINEIRQSNTDRVRRPTPGPSWRPESAQQQAHPQTPPRTRPQANSTPPPPKAKPNAGAERPSTQRPHNTPAADASAKVSDSAPAKSPVKPLYEHLGLSDMTADLSAVKTAYKKASL MNVVSHIAGKVALQVNSSLLEQKGRLNERQQKGLAVILDALRGKEPVTHVETHEGGGRFNLARAAFDVASVVWERDKSMHNVMSFLGVSDSQGKMLFSLGKKLADAMAKPEPAQGKDNCEAHHAFFSSNLKLNKLMNDIADQLINEIRQSNTDRVRRPTPGPSWRPESAQQQAHPQTPPRTRPQANSTPPPPKAKPNAGAERPSTQRPHNTPAADASAKVSDSAPAKSPVKPLYEHLGLSDMTADLSAVKTAYKKASL ``` |
| 1e-149 | capillary\_sequence\_57 | ``` C1E_0551 1-258  capillary_sequence_57 2-775 ``` | ``` MNVVSHIAGKVALQVNSSLLEQKGRLNERQQKGLAVILDALRGKEPVTHVETHEGGGRFNLARAAFDVASVVWERDKSMHNVMSFLGVSDSQGKMLFSLGKKLADAMAKPEPAQGKDNCEAHHAFFSSNLKLNKLMNDIADQLINEIRQSNTDRVRRPTPGPSWRPESAQQQAHPQTPPRTRPQANSTPPPPKAKPNAGAERPSTQRPHNTPAADASAKVSDSAPAKSPVKPLYEHLGLSDMTADLSAVKTAYKKASL MNVVSHIAGKVALQVNSSLLEQKGRLNERQQKGLAVILDALRGKEPVTHVETHEGGGRFNLARAAFDVASVVWERDKSMHNVMSFLGVSDSQGKMLFSLGKKLADAMAKPEPAQGKDNCEAHHAFFSSNLKLNKLMNDIADQLINEIRQSNTDRVRRPTPGPSWRPESAQQQAHPQTPPRTRPQANSTPPPPKAKPNAGAERPSTQRPHNTPAADASAKVSDSAPAKSPVKPLYEHLGLSDMTADLSAVKTAYKKASL MNVVSHIAGKVALQVNSSLLEQKGRLNERQQKGLAVILDALRGKEPVTHVETHEGGGRFNLARAAFDVASVVWERDKSMHNVMSFLGVSDSQGKMLFSLGKKLADAMAKPEPAQGKDNCEAHHAFFSSNLKLNKLMNDIADQLINEIRQSNTDRVRRPTPGPSWRPESAQQQAHPQTPPRTRPQANSTPPPPKAKPNAGAERPSTQRPHNTPAADASAKVSDSAPAKSPVKPLYEHLGLSDMTADLSAVKTAYKKASL ``` |
| 1e-149 | capillary\_sequence\_20 | ``` C1E_0551 1-258  capillary_sequence_20 2-775 ``` | ``` MNVVSHIAGKVALQVNSSLLEQKGRLNERQQKGLAVILDALRGKEPVTHVETHEGGGRFNLARAAFDVASVVWERDKSMHNVMSFLGVSDSQGKMLFSLGKKLADAMAKPEPAQGKDNCEAHHAFFSSNLKLNKLMNDIADQLINEIRQSNTDRVRRPTPGPSWRPESAQQQAHPQTPPRTRPQANSTPPPPKAKPNAGAERPSTQRPHNTPAADASAKVSDSAPAKSPVKPLYEHLGLSDMTADLSAVKTAYKKASL MNVVSHIAGKVALQVNSSLLEQKGRLNERQQKGLAVILDALRGKEPVTHVETHEGGGRFNLARAAFDVASVVWERDKSMHNVMSFLGVSDSQGKMLFSLGKKLADAMAKPEPAQGKDNCEAHHAFFSSNLKLNKLMNDIADQLINEIRQSNTDRVRRPTPGPSWRPESAQQQAHPQTPPRTRPQANSTPPPPKAKPNAGAERPSTQRPHNTPAADASAKVSDSAPAKSPVKPLYEHLGLSDMTADLSAVKTAYKKASL MNVVSHIAGKVALQVNSSLLEQKGRLNERQQKGLAVILDALRGKEPVTHVETHEGGGRFNLARAAFDVASVVWERDKSMHNVMSFLGVSDSQGKMLFSLGKKLADAMAKPEPAQGKDNCEAHHAFFSSNLKLNKLMNDIADQLINEIRQSNTDRVRRPTPGPSWRPESAQQQAHPQTPPRTRPQANSTPPPPKAKPNAGAERPSTQRPHNTPAADASAKVSDSAPAKSPVKPLYEHLGLSDMTADLSAVKTAYKKASL ``` |
| 1e-143 | capillary\_sequence\_58 | ``` C1E_0551 1-246  capillary_sequence_58 106-843 ``` | ``` MNVVSHIAGKVALQVNSSLLEQKGRLNERQQKGLAVILDALRGKEPVTHVETHEGGGRFNLARAAFDVASVVWERDKSMHNVMSFLGVSDSQGKMLFSLGKKLADAMAKPEPAQGKDNCEAHHAFFSSNLKLNKLMNDIADQLINEIRQSNTDRVRRPTPGPSWRPESAQQQAHPQTPPRTRPQANSTPPPPKAKPNAGAERPSTQRPHNTPAADASAKVSDSAPAKSPVKPLYEHLGLSDMTADL MNVVSHIAGKVALQVNSSLLEQKGRLNERQQKGLAVILDALRGKEPVTHVETHEGGGRFNLARAAFDVASVVWERDKSMHNVMSFLGVSDSQGKMLFSLGKKLADAMAKPEPAQGKDNCEAHHAFFSSNLKLNKLMNDIADQLINEIRQSNTDRVRRPTPGPSWRPESAQQQAHPQTPPRTRPQANSTPPPPKAKPNAGAERPSTQRPHNTPAADASAKVSDSAPAKSPVKPLYEHLGLSDMTADL MNVVSHIAGKVALQVNSSLLEQKGRLNERQQKGLAVILDALRGKEPVTHVETHEGGGRFNLARAAFDVASVVWERDKSMHNVMSFLGVSDSQGKMLFSLGKKLADAMAKPEPAQGKDNCEAHHAFFSSNLKLNKLMNDIADQLINEIRQSNTDRVRRPTPGPSWRPESAQQQAHPQTPPRTRPQANSTPPPPKAKPNAGAERPSTQRPHNTPAADASAKVSDSAPAKSPVKPLYEHLGLSDMTADL ``` |
| 1e-143 | capillary\_sequence\_1 | ``` C1E_0551 1-246  capillary_sequence_1 106-843 ``` | ``` MNVVSHIAGKVALQVNSSLLEQKGRLNERQQKGLAVILDALRGKEPVTHVETHEGGGRFNLARAAFDVASVVWERDKSMHNVMSFLGVSDSQGKMLFSLGKKLADAMAKPEPAQGKDNCEAHHAFFSSNLKLNKLMNDIADQLINEIRQSNTDRVRRPTPGPSWRPESAQQQAHPQTPPRTRPQANSTPPPPKAKPNAGAERPSTQRPHNTPAADASAKVSDSAPAKSPVKPLYEHLGLSDMTADL MNVVSHIAGKVALQVNSSLLEQKGRLNERQQKGLAVILDALRGKEPVTHVETHEGGGRFNLARAAFDVASVVWERDKSMHNVMSFLGVSDSQGKMLFSLGKKLADAMAKPEPAQGKDNCEAHHAFFSSNLKLNKLMNDIADQLINEIRQSNTDRVRRPTPGPSWRPESAQQQAHPQTPPRTRPQANSTPPPPKAKPNAGAERPSTQRPHNTPAADASAKVSDSAPAKSPVKPLYEHLGLSDMTADL MNVVSHIAGKVALQVNSSLLEQKGRLNERQQKGLAVILDALRGKEPVTHVETHEGGGRFNLARAAFDVASVVWERDKSMHNVMSFLGVSDSQGKMLFSLGKKLADAMAKPEPAQGKDNCEAHHAFFSSNLKLNKLMNDIADQLINEIRQSNTDRVRRPTPGPSWRPESAQQQAHPQTPPRTRPQANSTPPPPKAKPNAGAERPSTQRPHNTPAADASAKVSDSAPAKSPVKPLYEHLGLSDMTADL ``` |
| 1e-124 | capillary\_sequence\_56 | ``` C1E_0551 1-175  capillary_sequence_56 106-669 ``` | ``` MNVVSHIAGKVALQVNSSLLEQKGRLNERQQKGLAVILDALRGKEPVTHVETHEGGGRFNLARAAFDVASVVWERDKSMHNVMSFLGVSDSQGKMLFSLGKKLADAMAKPEPAQGKDNCEAHHAFFSSNLKLNKLMNDIADQLINEIRQSNTDRVRR---PTPGPSWRPES----------AQQQAHP MNVVSHIAGKVALQVNSSLLEQKGRLNERQQKGLAVILDALRGKEPVTHVETHEGGGRFNLARAAFDVASVVWERDKSMHNVMSFLGVSDSQGKMLFSLGKKLADAMAKPEPAQGKDNCEAHHAFFSSNLKLNKLMNDIADQLINEIRQSNTDRVRR     PG    P S          A+ Q HP MNVVSHIAGKVALQVNSSLLEQKGRLNERQQKGLAVILDALRGKEPVTHVETHEGGGRFNLARAAFDVASVVWERDKSMHNVMSFLGVSDSQGKMLFSLGKKLADAMAKPEPAQGKDNCEAHHAFFSSNLKLNKLMNDIADQLINEIRQSNTDRVRRXHLAHPGDLKAPNSKHIRKRLHALARXQQHP ``` |
| 1e-124 | capillary\_sequence\_56 | ``` C1E_0551 183-258  capillary_sequence_56 650-877 ``` | ```                                                                                                                                                                                       PQANSTPPPPKAKPNAGAERPSTQRPHNTPAADASAKVSDSAPAKSPVKPLYEHLGLSDMTADLSAVKTAYKKASL                                                                                                                                                                                       P ANSTPPPPKAKPNAGAERPSTQRPHNTPAADASAKVSDSAPAKSPVKPLYEHLGLSDMTADL   +TAY KASL                                                                                                                                                                                       PAANSTPPPPKAKPNAGAERPSTQRPHNTPAADASAKVSDSAPAKSPVKPLYEHLGLSDMTADLXXSQTAYXKASL ``` |

---

### **C1E\_1425** 554:155221..156516 Acetylornithine deacetylase/Succinyl-diaminopimelate desuccinylase and related deacylases Operon 80 Gene 1 COG0624 Acetylornithine deacetylase/Succinyl-diaminopimelate desuccinylase and related deacylases

|  |  |  |  |
| --- | --- | --- | --- |
| 0.0 | capillary\_sequence\_28 | ``` C1E_1425 1-431  capillary_sequence_28 251-1543 ``` | ``` MKKSISLLTTLFFCHGALAGTMLDEQSITSYVDSHSAEQVALLEKLVNINSGTDNVDGVVKVGDLMKAELEALGFDTRWHELPAGMNHAGSLVAVHDGNKSAKRILLIGHLDTVFPATSKFQEYSLIDGGNKAKGPGVIDDKGGLVTILYALQALKHSGALQDMNISVVLVGDEELAAKPTEISREWLIAEAKRSDIALGFEFALSPNQLITERRGLSEWFLTSTGIDKHSATIFQPETGFGAVYESARVLDEIRSKLSGEQGLTINPGLILGGATAQEDVPSGQGTASGRKTTIARIVSVHGDLRFSSEEQRSSAEARLQEIASHSLPQTHSELKIKAIMPVMVDRESNRKLLQAYSKVSQDLDGPALESAPSAERGGADISYVNKYVTASLDGLGAWGQGAHSENETIDLSSLPVVAKRAALFISRYGK MKKSISLLTTLFFCHGALAGTMLDEQSITSYVDSHSAEQVALLEKLVNINSGTDNVDGVVKVGDLMKAELEALGFDTRWHELPAGMNHAGSLVAVHDGNKSAKRILLIGHLDTVFPATSKFQEYSLIDGGNKAKGPGVIDDKGGLVTILYALQALKHSGALQDMNISVVLVGDEELAAKPTEISREWLIAEAKRSDIALGFEFALSPNQLITERRGLSEWFLTSTGIDKHSATIFQPETGFGAVYESARVLDEIRSKLSGEQGLTINPGLILGGATAQEDVPSGQGTASGRKTTIARIVSVHGDLRFSSEEQRSSAEARLQEIASHSLPQTHSELKIKAIMPVMVDRESNRKLLQAYSKVSQDLDGPALESAPSAERGGADISYVNKYVTASLDGLGAWGQGAHSENETIDLSSLPVVAKRAALFISRYGK MKKSISLLTTLFFCHGALAGTMLDEQSITSYVDSHSAEQVALLEKLVNINSGTDNVDGVVKVGDLMKAELEALGFDTRWHELPAGMNHAGSLVAVHDGNKSAKRILLIGHLDTVFPATSKFQEYSLIDGGNKAKGPGVIDDKGGLVTILYALQALKHSGALQDMNISVVLVGDEELAAKPTEISREWLIAEAKRSDIALGFEFALSPNQLITERRGLSEWFLTSTGIDKHSATIFQPETGFGAVYESARVLDEIRSKLSGEQGLTINPGLILGGATAQEDVPSGQGTASGRKTTIARIVSVHGDLRFSSEEQRSSAEARLQEIASHSLPQTHSELKIKAIMPVMVDRESNRKLLQAYSKVSQDLDGPALESAPSAERGGADISYVNKYVTASLDGLGAWGQGAHSENETIDLSSLPVVAKRAALFISRYGK ``` |
| 0.0 | capillary\_sequence\_74 | ``` C1E_1425 64-407  capillary_sequence_74 3-1034 ``` | ```                                                                DLMKAELEALGFDTRWHELPAGMNHAGSLVAVHDGNKSAKRILLIGHLDTVFPATSKFQEYSLIDGGNKAKGPGVIDDKGGLVTILYALQALKHSGALQDMNISVVLVGDEELAAKPTEISREWLIAEAKRSDIALGFEFALSPNQLITERRGLSEWFLTSTGIDKHSATIFQPETGFGAVYESARVLDEIRSKLSGEQGLTINPGLILGGATAQEDVPSGQGTASGRKTTIARIVSVHGDLRFSSEEQRSSAEARLQEIASHSLPQTHSELKIKAIMPVMVDRESNRKLLQAYSKVSQDLDGPALESAPSAERGGADISYVNKYVTASLDGLGAWGQGAHSEN                                                                DLMKAELEALGFDTRWHELPAGMNHAGSLVAVHDGNKSAKRILLIGHLDTVFPATSKFQEYSLIDGGNKAKGPGVIDDKGGLVTILYALQALKHSGALQDMNISVVLVGDEELAAKPTEISREWLIAEAKRSDIALGFEFALSPNQLITERRGLSEWFLTSTGIDKHSATIFQPETGFGAVYESARVLDEIRSKLSGEQGLTINPGLILGGATAQEDVPSGQGTASGRKTTIARIVSVHGDLRFSSEEQRSSAEARLQEIASHSLPQTHSELKIKAIMPVMVDRESNRKLLQAYSKVSQDLDGPALESAPSAERGGADISYVNKYVTASLDGLGAWGQGAHSEN                                                                DLMKAELEALGFDTRWHELPAGMNHAGSLVAVHDGNKSAKRILLIGHLDTVFPATSKFQEYSLIDGGNKAKGPGVIDDKGGLVTILYALQALKHSGALQDMNISVVLVGDEELAAKPTEISREWLIAEAKRSDIALGFEFALSPNQLITERRGLSEWFLTSTGIDKHSATIFQPETGFGAVYESARVLDEIRSKLSGEQGLTINPGLILGGATAQEDVPSGQGTASGRKTTIARIVSVHGDLRFSSEEQRSSAEARLQEIASHSLPQTHSELKIKAIMPVMVDRESNRKLLQAYSKVSQDLDGPALESAPSAERGGADISYVNKYVTASLDGLGAWGQGAHSEN ``` |
| 0.0 | capillary\_sequence\_8 | ``` C1E_1425 64-407  capillary_sequence_8 1-1032 ``` | ```                                                                DLMKAELEALGFDTRWHELPAGMNHAGSLVAVHDGNKSAKRILLIGHLDTVFPATSKFQEYSLIDGGNKAKGPGVIDDKGGLVTILYALQALKHSGALQDMNISVVLVGDEELAAKPTEISREWLIAEAKRSDIALGFEFALSPNQLITERRGLSEWFLTSTGIDKHSATIFQPETGFGAVYESARVLDEIRSKLSGEQGLTINPGLILGGATAQEDVPSGQGTASGRKTTIARIVSVHGDLRFSSEEQRSSAEARLQEIASHSLPQTHSELKIKAIMPVMVDRESNRKLLQAYSKVSQDLDGPALESAPSAERGGADISYVNKYVTASLDGLGAWGQGAHSEN                                                                DLMKAELEALGFDTRWHELPAGMNHAGSLVAVHDGNKSAKRILLIGHLDTVFPATSKFQEYSLIDGGNKAKGPGVIDDKGGLVTILYALQALKHSGALQDMNISVVLVGDEELAAKPTEISREWLIAEAKRSDIALGFEFALSPNQLITERRGLSEWFLTSTGIDKHSATIFQPETGFGAVYESARVLDEIRSKLSGEQGLTINPGLILGGATAQEDVPSGQGTASGRKTTIARIVSVHGDLRFSSEEQRSSAEARLQEIASHSLPQTHSELKIKAIMPVMVDRESNRKLLQAYSKVSQDLDGPALESAPSAERGGADISYVNKYVTASLDGLGAWGQGAHSEN                                                                DLMKAELEALGFDTRWHELPAGMNHAGSLVAVHDGNKSAKRILLIGHLDTVFPATSKFQEYSLIDGGNKAKGPGVIDDKGGLVTILYALQALKHSGALQDMNISVVLVGDEELAAKPTEISREWLIAEAKRSDIALGFEFALSPNQLITERRGLSEWFLTSTGIDKHSATIFQPETGFGAVYESARVLDEIRSKLSGEQGLTINPGLILGGATAQEDVPSGQGTASGRKTTIARIVSVHGDLRFSSEEQRSSAEARLQEIASHSLPQTHSELKIKAIMPVMVDRESNRKLLQAYSKVSQDLDGPALESAPSAERGGADISYVNKYVTASLDGLGAWGQGAHSEN ``` |
| 1e-175 | capillary\_sequence\_72 | ``` C1E_1425 70-407  capillary_sequence_72 1-1014 ``` | ```                                                                      LEALGFDTRWHELPAGMNHAGSLVAVHDGNKSAKRILLIGHLDTVFPATSKFQEYSLIDGGNKAKGPGVIDDKGGLVTILYALQALKHSGALQDMNISVVLVGDEELAAKPTEISREWLIAEAKRSDIALGFEFALSPNQLITERRGLSEWFLTSTGIDKHSATIFQPETGFGAVYESARVLDEIRSKLSGEQGLTINPGLILGGATAQEDVPSGQGTASGRKTTIARIVSVHGDLRFSSEEQRSSAEARLQEIASHSLPQTHSELKIKAIMPVMVDRESNRKLLQAYSKVSQDLDGPALESAPSAERGGADISYVNKYVTASLDGLGAWGQGAHSEN                                                                      +EA GFDTRWHELPAGMNHAGSL                      F ATSKFQEYSLIDGGNKAKGPGVIDDKGGLVTILYALQALKHSGALQDMNISVVLVGDEELAAKPTEISREWLIAEAKRSDIALGFEFALSPNQLITERRGLSEWFLTSTGIDKHSATIFQPETGFGAVYESARVLDEIRSKLSGEQGLTINPGLILGGATAQEDVPSGQGTASGRKTTIARIVSVHGDLRFSSEEQRSSAEARLQEIASHSLPQTHSELKIKAIMPVMVDRESNRKLLQAYSKVSQDLDGPALESAPSAERGGADISYVNKYVTASLDGLGAWGQGAHSEN                                                                      VEAXGFDTRWHELPAGMNHAGSLSRCMTVTSXRNEFC**AIWTRCFRATSKFQEYSLIDGGNKAKGPGVIDDKGGLVTILYALQALKHSGALQDMNISVVLVGDEELAAKPTEISREWLIAEAKRSDIALGFEFALSPNQLITERRGLSEWFLTSTGIDKHSATIFQPETGFGAVYESARVLDEIRSKLSGEQGLTINPGLILGGATAQEDVPSGQGTASGRKTTIARIVSVHGDLRFSSEEQRSSAEARLQEIASHSLPQTHSELKIKAIMPVMVDRESNRKLLQAYSKVSQDLDGPALESAPSAERGGADISYVNKYVTASLDGLGAWGQGAHSEN ``` |
| 1e-144 | capillary\_sequence\_75 | ``` C1E_1425 1-254  capillary_sequence_75 215-976 ``` | ``` MKKSISLLTTLFFCHGALAGTMLDEQSITSYVDSHSAEQVALLEKLVNINSGTDNVDGVVKVGDLMKAELEALGFDTRWHELPAGMNHAGSLVAVHDGNKSAKRILLIGHLDTVFPATSKFQEYSLIDGGNKAKGPGVIDDKGGLVTILYALQALKHSGALQDMNISVVLVGDEELAAKPTEISREWLIAEAKRSDIALGFEFALSPNQLITERRGLSEWFLTSTGIDKHSATIFQPETGFGAVYESARVLDEI MKKSISLLTTLFFCHGALAGTMLDEQSITSYVDSHSAEQVALLEKLVNINSGTDNVDGVVKVGDLMKAELEALGFDTRWHELPAGMNHAGSLVAVHDGNKSAKRILLIGHLDTVFPATSKFQEYSLIDGGNKAKGPGVIDDKGGLVTILYALQALKHSGALQDMNISVVLVGDEELAAKPTEISREWLIAEAKRSDIALGFEFALSPNQLITERRGLSEWFLTSTGIDKHSATIFQPETGFGAVYESARVLDEI MKKSISLLTTLFFCHGALAGTMLDEQSITSYVDSHSAEQVALLEKLVNINSGTDNVDGVVKVGDLMKAELEALGFDTRWHELPAGMNHAGSLVAVHDGNKSAKRILLIGHLDTVFPATSKFQEYSLIDGGNKAKGPGVIDDKGGLVTILYALQALKHSGALQDMNISVVLVGDEELAAKPTEISREWLIAEAKRSDIALGFEFALSPNQLITERRGLSEWFLTSTGIDKHSATIFQPETGFGAVYESARVLDEI ``` |
| 1e-144 | capillary\_sequence\_9 | ``` C1E_1425 1-254  capillary_sequence_9 215-976 ``` | ``` MKKSISLLTTLFFCHGALAGTMLDEQSITSYVDSHSAEQVALLEKLVNINSGTDNVDGVVKVGDLMKAELEALGFDTRWHELPAGMNHAGSLVAVHDGNKSAKRILLIGHLDTVFPATSKFQEYSLIDGGNKAKGPGVIDDKGGLVTILYALQALKHSGALQDMNISVVLVGDEELAAKPTEISREWLIAEAKRSDIALGFEFALSPNQLITERRGLSEWFLTSTGIDKHSATIFQPETGFGAVYESARVLDEI MKKSISLLTTLFFCHGALAGTMLDEQSITSYVDSHSAEQVALLEKLVNINSGTDNVDGVVKVGDLMKAELEALGFDTRWHELPAGMNHAGSLVAVHDGNKSAKRILLIGHLDTVFPATSKFQEYSLIDGGNKAKGPGVIDDKGGLVTILYALQALKHSGALQDMNISVVLVGDEELAAKPTEISREWLIAEAKRSDIALGFEFALSPNQLITERRGLSEWFLTSTGIDKHSATIFQPETGFGAVYESARVLDEI MKKSISLLTTLFFCHGALAGTMLDEQSITSYVDSHSAEQVALLEKLVNINSGTDNVDGVVKVGDLMKAELEALGFDTRWHELPAGMNHAGSLVAVHDGNKSAKRILLIGHLDTVFPATSKFQEYSLIDGGNKAKGPGVIDDKGGLVTILYALQALKHSGALQDMNISVVLVGDEELAAKPTEISREWLIAEAKRSDIALGFEFALSPNQLITERRGLSEWFLTSTGIDKHSATIFQPETGFGAVYESARVLDEI ``` |
| 1e-141 | capillary\_sequence\_73 | ``` C1E_1425 1-262  capillary_sequence_73 226-1011 ``` | ``` MKKSISLLTTLFFCHGALAGTMLDEQSITSYVDSHSAEQVALLEKLVNINSGTDNVDGVVKVGDLMKAELEALGFDTRWHELPAGMNHAGSLVAVHDGNKSAKRILLIGHLDTVFPATSKFQEYSLIDGGNKAKGPGVIDDKGGLVTILYALQALKHSGALQDMNISVVLVGDEELAAKPTEISREWLIAEAKRSDIALGFEFALSPNQLITERRGLSEWFLTSTGIDKHSATIFQPETGFGAVYESARVLDEIRSKLSGEQ MKKSISLLTTLFFCHGALAGTMLDEQSITSYVDSHSAEQVALLEKLVNINSGTDNVDGVVKVGDLMKAELEALGFDTRWHELPAGMNHAGSLVAVHDGNKSAKRILLIGHLDTVFPATSKFQEYSLIDGGNKAKGPGVIDDKGGLVTILYALQALKHSGALQDMNISVVLVGDEELAAKPTEISREWLIAEAKRSDIALGFEFALSPNQLITERRGLSEWFLTSTGIDKHSATIFQPETGFGAVY SA  L +     S EQ MKKSISLLTTLFFCHGALAGTMLDEQSITSYVDSHSAEQVALLEKLVNINSGTDNVDGVVKVGDLMKAELEALGFDTRWHELPAGMNHAGSLVAVHDGNKSAKRILLIGHLDTVFPATSKFQEYSLIDGGNKAKGPGVIDDKGGLVTILYALQALKHSGALQDMNISVVLVGDEELAAKPTEISREWLIAEAKRSDIALGFEFALSPNQLITERRGLSEWFLTSTGIDKHSATIFQPETGFGAVYXSAXCLMKFXPNFSXEQ ``` |

---

### **C1E\_2036** 672:101352..102155 Operon 58 Gene 1 gi|68637852|emb|CAI36057.1| cysteine protease

|  |  |  |  |
| --- | --- | --- | --- |
| 1e-145 | capillary\_sequence\_54 | ``` C1E_2036 1-251  capillary_sequence_54 210-962 ``` | ``` MKINIATSSIAILHHQENHAPKASSAPQPEHAQTNQQMPLDLALRPKTRGIHPFLALMLGDKGCVSSSNVNLEDDSTTQVNLEDFAVASRDVNRNNICAGLSTEWLVMSNSGDAQSRMDHLDHNGEGQSNGAQRHQVYDNALASALSNDDEAPFFTASTAVIEDAGFSLRREPKTARASGGSAQLAQTLANDLAQTGRKHLLSLRFASVQGHAIACSCEGSRFKLFDPNLGEFQSSRSEAPQMLKALIDHY MKINIATSSIAILHHQENHAPKASSAPQPEHAQTNQQMPLDLALRPKTRGIHPFLALMLGDKGCVSSSNVNLEDDSTTQVNLEDFAVASRDVNRNNICAGLSTEWLVMSNSGDAQSRMDHLDHNGEGQSNGAQRHQVYDNALASALSNDDEAPFFTASTAVIEDAGFSLRREPKTARASGGSAQLAQTLANDLAQTGRKHLLSLRFASVQGHAIACSCEGSRFKLFDPNLGEFQSSRSEAPQMLKALIDHY MKINIATSSIAILHHQENHAPKASSAPQPEHAQTNQQMPLDLALRPKTRGIHPFLALMLGDKGCVSSSNVNLEDDSTTQVNLEDFAVASRDVNRNNICAGLSTEWLVMSNSGDAQSRMDHLDHNGEGQSNGAQRHQVYDNALASALSNDDEAPFFTASTAVIEDAGFSLRREPKTARASGGSAQLAQTLANDLAQTGRKHLLSLRFASVQGHAIACSCEGSRFKLFDPNLGEFQSSRSEAPQMLKALIDHY ``` |
| 1e-145 | capillary\_sequence\_52 | ``` C1E_2036 1-251  capillary_sequence_52 210-962 ``` | ``` MKINIATSSIAILHHQENHAPKASSAPQPEHAQTNQQMPLDLALRPKTRGIHPFLALMLGDKGCVSSSNVNLEDDSTTQVNLEDFAVASRDVNRNNICAGLSTEWLVMSNSGDAQSRMDHLDHNGEGQSNGAQRHQVYDNALASALSNDDEAPFFTASTAVIEDAGFSLRREPKTARASGGSAQLAQTLANDLAQTGRKHLLSLRFASVQGHAIACSCEGSRFKLFDPNLGEFQSSRSEAPQMLKALIDHY MKINIATSSIAILHHQENHAPKASSAPQPEHAQTNQQMPLDLALRPKTRGIHPFLALMLGDKGCVSSSNVNLEDDSTTQVNLEDFAVASRDVNRNNICAGLSTEWLVMSNSGDAQSRMDHLDHNGEGQSNGAQRHQVYDNALASALSNDDEAPFFTASTAVIEDAGFSLRREPKTARASGGSAQLAQTLANDLAQTGRKHLLSLRFASVQGHAIACSCEGSRFKLFDPNLGEFQSSRSEAPQMLKALIDHY MKINIATSSIAILHHQENHAPKASSAPQPEHAQTNQQMPLDLALRPKTRGIHPFLALMLGDKGCVSSSNVNLEDDSTTQVNLEDFAVASRDVNRNNICAGLSTEWLVMSNSGDAQSRMDHLDHNGEGQSNGAQRHQVYDNALASALSNDDEAPFFTASTAVIEDAGFSLRREPKTARASGGSAQLAQTLANDLAQTGRKHLLSLRFASVQGHAIACSCEGSRFKLFDPNLGEFQSSRSEAPQMLKALIDHY ``` |
| 1e-145 | capillary\_sequence\_35 | ``` C1E_2036 1-251  capillary_sequence_35 239-991 ``` | ``` MKINIATSSIAILHHQENHAPKASSAPQPEHAQTNQQMPLDLALRPKTRGIHPFLALMLGDKGCVSSSNVNLEDDSTTQVNLEDFAVASRDVNRNNICAGLSTEWLVMSNSGDAQSRMDHLDHNGEGQSNGAQRHQVYDNALASALSNDDEAPFFTASTAVIEDAGFSLRREPKTARASGGSAQLAQTLANDLAQTGRKHLLSLRFASVQGHAIACSCEGSRFKLFDPNLGEFQSSRSEAPQMLKALIDHY MKINIATSSIAILHHQENHAPKASSAPQPEHAQTNQQMPLDLALRPKTRGIHPFLALMLGDKGCVSSSNVNLEDDSTTQVNLEDFAVASRDVNRNNICAGLSTEWLVMSNSGDAQSRMDHLDHNGEGQSNGAQRHQVYDNALASALSNDDEAPFFTASTAVIEDAGFSLRREPKTARASGGSAQLAQTLANDLAQTGRKHLLSLRFASVQGHAIACSCEGSRFKLFDPNLGEFQSSRSEAPQMLKALIDHY MKINIATSSIAILHHQENHAPKASSAPQPEHAQTNQQMPLDLALRPKTRGIHPFLALMLGDKGCVSSSNVNLEDDSTTQVNLEDFAVASRDVNRNNICAGLSTEWLVMSNSGDAQSRMDHLDHNGEGQSNGAQRHQVYDNALASALSNDDEAPFFTASTAVIEDAGFSLRREPKTARASGGSAQLAQTLANDLAQTGRKHLLSLRFASVQGHAIACSCEGSRFKLFDPNLGEFQSSRSEAPQMLKALIDHY ``` |
| 1e-145 | capillary\_sequence\_17 | ``` C1E_2036 1-251  capillary_sequence_17 210-962 ``` | ``` MKINIATSSIAILHHQENHAPKASSAPQPEHAQTNQQMPLDLALRPKTRGIHPFLALMLGDKGCVSSSNVNLEDDSTTQVNLEDFAVASRDVNRNNICAGLSTEWLVMSNSGDAQSRMDHLDHNGEGQSNGAQRHQVYDNALASALSNDDEAPFFTASTAVIEDAGFSLRREPKTARASGGSAQLAQTLANDLAQTGRKHLLSLRFASVQGHAIACSCEGSRFKLFDPNLGEFQSSRSEAPQMLKALIDHY MKINIATSSIAILHHQENHAPKASSAPQPEHAQTNQQMPLDLALRPKTRGIHPFLALMLGDKGCVSSSNVNLEDDSTTQVNLEDFAVASRDVNRNNICAGLSTEWLVMSNSGDAQSRMDHLDHNGEGQSNGAQRHQVYDNALASALSNDDEAPFFTASTAVIEDAGFSLRREPKTARASGGSAQLAQTLANDLAQTGRKHLLSLRFASVQGHAIACSCEGSRFKLFDPNLGEFQSSRSEAPQMLKALIDHY MKINIATSSIAILHHQENHAPKASSAPQPEHAQTNQQMPLDLALRPKTRGIHPFLALMLGDKGCVSSSNVNLEDDSTTQVNLEDFAVASRDVNRNNICAGLSTEWLVMSNSGDAQSRMDHLDHNGEGQSNGAQRHQVYDNALASALSNDDEAPFFTASTAVIEDAGFSLRREPKTARASGGSAQLAQTLANDLAQTGRKHLLSLRFASVQGHAIACSCEGSRFKLFDPNLGEFQSSRSEAPQMLKALIDHY ``` |
| 1e-140 | capillary\_sequence\_55 | ``` C1E_2036 1-243  capillary_sequence_55 239-967 ``` | ``` MKINIATSSIAILHHQENHAPKASSAPQPEHAQTNQQMPLDLALRPKTRGIHPFLALMLGDKGCVSSSNVNLEDDSTTQVNLEDFAVASRDVNRNNICAGLSTEWLVMSNSGDAQSRMDHLDHNGEGQSNGAQRHQVYDNALASALSNDDEAPFFTASTAVIEDAGFSLRREPKTARASGGSAQLAQTLANDLAQTGRKHLLSLRFASVQGHAIACSCEGSRFKLFDPNLGEFQSSRSEAPQM MKINIATSSIAILHHQENHAPKASSAPQPEHAQTNQQMPLDLALRPKTRGIHPFLALMLGDKGCVSSSNVNLEDDSTTQVNLEDFAVASRDVNRNNICAGLSTEWLVMSNSGDAQSRMDHLDHNGEGQSNGAQRHQVYDNALASALSNDDEAPFFTASTAVIEDAGFSLRREPKTARASGGSAQLAQTLANDLAQTGRKHLLSLRFASVQGHAIACSCEGSRFKLFDPNLGEFQSSRSEAPQM MKINIATSSIAILHHQENHAPKASSAPQPEHAQTNQQMPLDLALRPKTRGIHPFLALMLGDKGCVSSSNVNLEDDSTTQVNLEDFAVASRDVNRNNICAGLSTEWLVMSNSGDAQSRMDHLDHNGEGQSNGAQRHQVYDNALASALSNDDEAPFFTASTAVIEDAGFSLRREPKTARASGGSAQLAQTLANDLAQTGRKHLLSLRFASVQGHAIACSCEGSRFKLFDPNLGEFQSSRSEAPQM ``` |
| 1e-140 | capillary\_sequence\_16 | ``` C1E_2036 1-243  capillary_sequence_16 1-729 ``` | ``` MKINIATSSIAILHHQENHAPKASSAPQPEHAQTNQQMPLDLALRPKTRGIHPFLALMLGDKGCVSSSNVNLEDDSTTQVNLEDFAVASRDVNRNNICAGLSTEWLVMSNSGDAQSRMDHLDHNGEGQSNGAQRHQVYDNALASALSNDDEAPFFTASTAVIEDAGFSLRREPKTARASGGSAQLAQTLANDLAQTGRKHLLSLRFASVQGHAIACSCEGSRFKLFDPNLGEFQSSRSEAPQM MKINIATSSIAILHHQENHAPKASSAPQPEHAQTNQQMPLDLALRPKTRGIHPFLALMLGDKGCVSSSNVNLEDDSTTQVNLEDFAVASRDVNRNNICAGLSTEWLVMSNSGDAQSRMDHLDHNGEGQSNGAQRHQVYDNALASALSNDDEAPFFTASTAVIEDAGFSLRREPKTARASGGSAQLAQTLANDLAQTGRKHLLSLRFASVQGHAIACSCEGSRFKLFDPNLGEFQSSRSEAPQM MKINIATSSIAILHHQENHAPKASSAPQPEHAQTNQQMPLDLALRPKTRGIHPFLALMLGDKGCVSSSNVNLEDDSTTQVNLEDFAVASRDVNRNNICAGLSTEWLVMSNSGDAQSRMDHLDHNGEGQSNGAQRHQVYDNALASALSNDDEAPFFTASTAVIEDAGFSLRREPKTARASGGSAQLAQTLANDLAQTGRKHLLSLRFASVQGHAIACSCEGSRFKLFDPNLGEFQSSRSEAPQM ``` |
| 1e-139 | capillary\_sequence\_53 | ``` C1E_2036 1-243  capillary_sequence_53 1-729 ``` | ``` MKINIATSSIAILHHQENHAPKASSAPQPEHAQTNQQMPLDLALRPKTRGIHPFLALMLGDKGCVSSSNVNLEDDSTTQVNLEDFAVASRDVNRNNICAGLSTEWLVMSNSGDAQSRMDHLDHNGEGQSNGAQRHQVYDNALASALSNDDEAPFFTASTAVIEDAGFSLRREPKTARASGGSAQLAQTLANDLAQTGRKHLLSLRFASVQGHAIACSCEGSRFKLFDPNLGEFQSSRSEAPQM MKINIATSSIAILHHQENHAPKASSAPQPEHAQTNQQMPLDLALRPKTRGIHPFLALMLGDKGCVSSSNVNLEDDSTTQVNLEDFAVASRDVNRNNICAGLSTEWLVMSNSGDAQSRMDHLDHNGEGQSNGAQRHQVYDNALASALSNDDEAPFFTASTAVIEDAGFSLRREPKTARASGGSAQLAQTLANDLAQTGRKHLLSLRFASVQGHAIACSCEGSRFKLFDP+LGEFQSSRSEAPQM MKINIATSSIAILHHQENHAPKASSAPQPEHAQTNQQMPLDLALRPKTRGIHPFLALMLGDKGCVSSSNVNLEDDSTTQVNLEDFAVASRDVNRNNICAGLSTEWLVMSNSGDAQSRMDHLDHNGEGQSNGAQRHQVYDNALASALSNDDEAPFFTASTAVIEDAGFSLRREPKTARASGGSAQLAQTLANDLAQTGRKHLLSLRFASVQGHAIACSCEGSRFKLFDPHLGEFQSSRSEAPQM ``` |

---

### **C1E\_2087** 672:146130..147095 ABC-type nitrate/sulfonate/bicarbonate transport systems, periplasmic components Operon 88 Gene 1 COG0715 ABC-type nitrate/sulfonate/bicarbonate transport systems, periplasmic components

|  |  |  |  |
| --- | --- | --- | --- |
| 1e-171 | capillary\_sequence\_24 | ``` C1E_2087 1-321  capillary_sequence_24 798-1760 ``` | ``` MRTVILRRGLVALFAAAVALGAVTQAQAEDLRIGYQKYGTLVLLKAKGSLEKRLAEQGVKVQWTEFPGGPQLLEGLNVGSIDFGVTGETPPVFAQAAGADLLYVAYEPPAPTSEAILVHKDSPITSVKDLKGKKVVLNKGSNVHYLLVKALEDAGLKYTDIQTVFLPPADARAAFERGSVDAWVIWDPYQAAAEKQLQARTLKDGTGIVDNHQFYLATKPYAQKNPKVIQALIEEVRAVGEWSKAHPDEVTKQVAPLLGLPADITLTSVKRQGYGALFITPPVVAAQQKIADTFYQLKLIPKPLSIADVVWTPPAAVAQAQ MRTVILRRGLVALFAAAVALGAVTQAQAEDLRIGYQKYGTLVLLKAKGSLEKRLAEQGVKVQWTEFPGGPQLLEGLNVGSIDFGVTGETPPVFAQAAGADLLYVAYEPPAPTSEAILVHKDSPITSVKDLKGKKVVLNKGSNVHYLLVKALEDAGLKYTDIQTVFLPPADARAAFERGSVDAWVIWDPYQAAAEKQLQARTLKDGTGIVDNHQFYLATKPYAQKNPKVIQALIEEVRAVGEWSKAHPDEVTKQVAPLLGLPADITLTSVKRQGYGALFITPPVVAAQQKIADTFYQ    P+   I  V  +   AVAQAQ MRTVILRRGLVALFAAAVALGAVTQAQAEDLRIGYQKYGTLVLLKAKGSLEKRLAEQGVKVQWTEFPGGPQLLEGLNVGSIDFGVTGETPPVFAQAAGADLLYVAYEPPAPTSEAILVHKDSPITSVKDLKGKKVVLNKGSNVHYLLVKALEDAGLKYTDIQTVFLPPADARAAFERGSVDAWVIWDPYQAAAEKQLQARTLKDGTGIVDNHQFYLATKPYAQKNPKVIQALIEEVRAVGEWSKAHPDEVTKQVAPLLGLPADITLTSVKRQGYGALFITPPVVAAQQKIADTFYQGNSKPRHGRIKCVR*SLEDAVAQAQ ``` |
| 1e-117 | capillary\_sequence\_84 | ``` C1E_2087 131-321  capillary_sequence_84 315-887 ``` | ```                                                                                                                                   KGKKVVLNKGSNVHYLLVKALEDAGLKYTDIQTVFLPPADARAAFERGSVDAWVIWDPYQAAAEKQLQARTLKDGTGIVDNHQFYLATKPYAQKNPKVIQALIEEVRAVGEWSKAHPDEVTKQVAPLLGLPADITLTSVKRQGYGALFITPPVVAAQQKIADTFYQLKLIPKPLSIADVVWTPPAAVAQAQ                                                                                                                                   KGKKVVLNKGSNVHYLLVKALEDAGLKYTDIQTVFLPPADARAAFERGSVDAWVIWDPYQAAAEKQLQARTLKDGTGIVDNHQFYLATKPYAQKNPKVIQALIEEVRAVGEWSKAHPDEVTKQVAPLLGLPADITLTSVKRQGYGALFITPPVVAAQQKIADTFYQLKLIPKPLSIADVVWTPPAAVAQAQ                                                                                                                                   KGKKVVLNKGSNVHYLLVKALEDAGLKYTDIQTVFLPPADARAAFERGSVDAWVIWDPYQAAAEKQLQARTLKDGTGIVDNHQFYLATKPYAQKNPKVIQALIEEVRAVGEWSKAHPDEVTKQVAPLLGLPADITLTSVKRQGYGALFITPPVVAAQQKIADTFYQLKLIPKPLSIADVVWTPPAAVAQAQ ``` |
| 1e-117 | capillary\_sequence\_84 | ``` C1E_2087 109-134  capillary_sequence_84 875-952 ``` | ```                                                                                                             PAPTSEAILVHKDSPITSVKDLKGKK                                                                                                             PAPTSEAILVHKDSPITSVK  K +K                                                                                                             PAPTSEAILVHKDSPITSVKXXKARK ``` |
| 1e-117 | capillary\_sequence\_84 | ``` C1E_2087 95-105  capillary_sequence_84 961-993 ``` | ```                                                                                               QAAGADLLYVA                                                                                               QAAGADLLYVA                                                                                               QAAGADLLYVA ``` |
| 1e-117 | capillary\_sequence\_41 | ``` C1E_2087 131-321  capillary_sequence_41 315-887 ``` | ```                                                                                                                                   KGKKVVLNKGSNVHYLLVKALEDAGLKYTDIQTVFLPPADARAAFERGSVDAWVIWDPYQAAAEKQLQARTLKDGTGIVDNHQFYLATKPYAQKNPKVIQALIEEVRAVGEWSKAHPDEVTKQVAPLLGLPADITLTSVKRQGYGALFITPPVVAAQQKIADTFYQLKLIPKPLSIADVVWTPPAAVAQAQ                                                                                                                                   KGKKVVLNKGSNVHYLLVKALEDAGLKYTDIQTVFLPPADARAAFERGSVDAWVIWDPYQAAAEKQLQARTLKDGTGIVDNHQFYLATKPYAQKNPKVIQALIEEVRAVGEWSKAHPDEVTKQVAPLLGLPADITLTSVKRQGYGALFITPPVVAAQQKIADTFYQLKLIPKPLSIADVVWTPPAAVAQAQ                                                                                                                                   KGKKVVLNKGSNVHYLLVKALEDAGLKYTDIQTVFLPPADARAAFERGSVDAWVIWDPYQAAAEKQLQARTLKDGTGIVDNHQFYLATKPYAQKNPKVIQALIEEVRAVGEWSKAHPDEVTKQVAPLLGLPADITLTSVKRQGYGALFITPPVVAAQQKIADTFYQLKLIPKPLSIADVVWTPPAAVAQAQ ``` |
| 1e-117 | capillary\_sequence\_41 | ``` C1E_2087 109-134  capillary_sequence_41 875-952 ``` | ```                                                                                                             PAPTSEAILVHKDSPITSVKDLKGKK                                                                                                             PAPTSEAILVHKDSPITSVKD K +K                                                                                                             PAPTSEAILVHKDSPITSVKDRKARK ``` |

---

### **C1E\_3964** 955:74860..77184 Operon 49 Gene 1 gi|49188583|ref|YP\_025680.1| type III secreted effector hopPmaA

|  |  |  |  |
| --- | --- | --- | --- |
| 0.0 | capillary\_sequence\_89 | ``` C1E_3964 1-774  capillary_sequence_89 389-2713 ``` | ``` MSPAQIIRTPHSFPPSFTGTSSSAENSHAQSPQQVLTRAFVASGELNAAFGRTSTASEQDFTSLLGTLQRELERKTLSFPDIAELANQLAEAAKGDQGGHWLGRDEQQTLKGMIDRCKSQLAHTHASDASYDPLAQVCENLKTARLHQSIRQMTGEAHAKVRGVPDLLALIQLDPDVLAEKPVGMTSYVNFGSFICMAKARTAELSEDLRSDPNEVALLLHPHADTILELERLPDALAALTENCPDTPTRDDLRSLAKETGELLQQLRANDLLPRSEEVSSYQGETSVRSREVVEPKLTLCQAGGNGQGQLEASSARPESLRYAPTRAASSGSEARVPGQAVGGKIADDAQKVAGLYAEKKRTNWTQANGVAGKISHKIQSLLGMRDAGSRVQAFVAFMADGKGRPGATMLDLGD-----------GWMRATRVIKGEAALIDFQCDSDGKVVDARHPGRFPVLPQGNEREAFKTVLQELKFRGAETLSKVPVYYVNRNTRGYVIPTHGYVVAGHPNRGRKSGAVLYGVGGDPKRGPVALDEKLLGHLVGRSDSKTSSKLSAPVKAAISALAGASFATREDFYDAYCAVRGDAVDPLERHNEISSIYRLLPLSTMEMWPKKADDYRVARPAAPERDLRAFENLPKDIGRKAQLKKVSNVDSIDLLEAKRQFTLHQLYQDEMLGRNGTGVPSADFKPKVDAQRRDQLVASTPKFQRLPPHTTDKVGNCNTGASSLLQRAVDTYTEKNNLPPEKVTAASIFGIGSSHRLAIWDPLDGSSSNKSSKDR MSPAQIIRTPHSFPPSFTGTSSSAENSHAQSPQQVLTRAFVASGELNAAFGRTSTASEQDFTSLLGTLQRELERKTLSFPDIAELANQLAEAAKGDQGGHWLGRDEQQTLKGMIDRCKSQLAHTHASDASYDPLAQVCENLKTARLHQSIRQMTGEAHAKVRGVPDLLALIQLDPDVLAEKPVGMTSYVNFGSFICMAKARTAELSEDLRSDPNEVALLLHPHADTILELERLPDALAALTENCPDTPTRDDLRSLAKETGELLQQLRANDLLPRSEEVSSYQGETSVRSREVVEPKLTLCQAGGNGQGQLEASSARPESLRYAPTRAASSGSEARVPGQAVGGKIADDAQKVAGLYAEKKR     A G  G   ++ Q  +   DA  RV           GR  +  ++ G            G   ATRVIKGEAALIDFQCDSDGKVVDARHPGRFPVLPQGNEREAFKTVLQELKFRGAETLSKVPVYYVNRNTRGYVIPTHGYVVAGHPNRGRKSGAVLYGVGGDPKRGPVALDEKLLGHLVGRSDSKTSSKLSAPVKAAISALAGASFATREDFYDAYCAVRGDAVDPLERHNEISSIYRLLPLSTMEMWPKKADDYRVARPAAPERDLRAFENLPKDIGRKAQLKKVSNVDSIDLLEAKRQFTLHQLYQDEMLGRNGTGVPSADFKPKVDAQRRDQLVASTPKFQRLPPHTTDKVGNCNTGASSLLQRAVDTYTEKNNLPPEKVTAASIFGIGSSHRLAIWDPLDGSSSNKSSKDR MSPAQIIRTPHSFPPSFTGTSSSAENSHAQSPQQVLTRAFVASGELNAAFGRTSTASEQDFTSLLGTLQRELERKTLSFPDIAELANQLAEAAKGDQGGHWLGRDEQQTLKGMIDRCKSQLAHTHASDASYDPLAQVCENLKTARLHQSIRQMTGEAHAKVRGVPDLLALIQLDPDVLAEKPVGMTSYVNFGSFICMAKARTAELSEDLRSDPNEVALLLHPHADTILELERLPDALAALTENCPDTPTRDDLRSLAKETGELLQQLRANDLLPRSEEVSSYQGETSVRSREVVEPKLTLCQAGGNGQGQLEASSARPESLRYAPTRAASSGSEARVPGQAVGGKIADDAQKVAGLYAEKKRQ---LAPGKRGSGENQPQDSIPAGDARFRVWC-------PGGRSHSWPMERGGPRRQRCSI*AMGG*GATRVIKGEAALIDFQCDSDGKVVDARHPGRFPVLPQGNEREAFKTVLQELKFRGAETLSKVPVYYVNRNTRGYVIPTHGYVVAGHPNRGRKSGAVLYGVGGDPKRGPVALDEKLLGHLVGRSDSKTSSKLSAPVKAAISALAGASFATREDFYDAYCAVRGDAVDPLERHNEISSIYRLLPLSTMEMWPKKADDYRVARPAAPERDLRAFENLPKDIGRKAQLKKVSNVDSIDLLEAKRQFTLHQLYQDEMLGRNGTGVPSADFKPKVDAQRRDQLVASTPKFQRLPPHTTDKVGNCNTGASSLLQRAVDTYTEKNNLPPEKVTAASIFGIGSSHRLAIWDPLDGSSSNKSSKDR ``` |
| 0.0 | capillary\_sequence\_29 | ``` C1E_3964 356-774  capillary_sequence_29 1452-2708 ``` | ```                                                                                                                                                                                                                                                                                                                                                                    LYAEKKRTNWTQANGVAGKISHKIQSLLGMRDAGSRVQAFVAFMADGKGRPGATMLDLGDGWMRATRVIKGEAALIDFQCDSDGKVVDARHPGRFPVLPQGNEREAFKTVLQELKFRGAETLSKVPVYYVNRNTRGYVIPTHGYVVAGHPNRGRKSGAVLYGVGGDPKRGPVALDEKLLGHLVGRSDSKTSSKLSAPVKAAISALAGASFATREDFYDAYCAVRGDAVDPLERHNEISSIYRLLPLSTMEMWPKKADDYRVARPAAPERDLRAFENLPKDIGRKAQLKKVSNVDSIDLLEAKRQFTLHQLYQDEMLGRNGTGVPSADFKPKVDAQRRDQLVASTPKFQRLPPHTTDKVGNCNTGASSLLQRAVDTYTEKNNLPPEKVTAASIFGIGSSHRLAIWDPLDGSSSNKSSKDR                                                                                                                                                                                                                                                                                                                                                                    ++ ++KR NW QANGV GKIS KIQSLLGMRD+G  VQ  VAFMADGKGRPGATMLDLGDGWMRATRVIKGEAALIDFQCDSDGKVVDARHPGRFPVLPQGNEREAFKTVLQELKFRGAETLSKVPVYYVNRNTRGYVIPTHGYVVAGHPNRGRKSGAVLYGVGGDPKRGPVALDEKLLGHLVGRSDSKTSSKLSAPVKAAISALAGASFATREDFYDAYCAVRGDAVDPLERHNEISSIYRLLPLSTMEMWPKKADDYRVARPAAPERDLRAFENLPKDIGRKAQLKKVSNVDSIDLLEAKRQFTLHQLYQDEMLGRNGTGVPSADFKPKVDAQRRDQLVASTPKFQRLPPHTTDKVGNCNTGASSLLQRAVDTYTEKNNLPPEKVTAASIFGIGSSHRLAIWDPLDGSSSNKSSKDR                                                                                                                                                                                                                                                                                                                                                                    VFMQRKRGNWPQANGVPGKISPKIQSLLGMRDSGFGVQGVVAFMADGKGRPGATMLDLGDGWMRATRVIKGEAALIDFQCDSDGKVVDARHPGRFPVLPQGNEREAFKTVLQELKFRGAETLSKVPVYYVNRNTRGYVIPTHGYVVAGHPNRGRKSGAVLYGVGGDPKRGPVALDEKLLGHLVGRSDSKTSSKLSAPVKAAISALAGASFATREDFYDAYCAVRGDAVDPLERHNEISSIYRLLPLSTMEMWPKKADDYRVARPAAPERDLRAFENLPKDIGRKAQLKKVSNVDSIDLLEAKRQFTLHQLYQDEMLGRNGTGVPSADFKPKVDAQRRDQLVASTPKFQRLPPHTTDKVGNCNTGASSLLQRAVDTYTEKNNLPPEKVTAASIFGIGSSHRLAIWDPLDGSSSNKSSKDR ``` |
| 0.0 | capillary\_sequence\_29 | ``` C1E_3964 1-392  capillary_sequence_29 389-1555 ``` | ``` MSPAQIIRTPHSFPPSFTGTSSSAENSHAQSPQQVLTRAFVASGELNAAFGRTSTASEQDFTSLLGTLQRELERKTLSFPDIAELANQLAEAAKGDQGGHWLGRDEQQTLKGMIDRCKSQLAHTHASDASYDPLAQVCENLKTARLHQSIRQMTGEAHAKVRGVPDLLALIQLDPDVLAEKPVGMTSYVNFGSFICMAKARTAELSEDLRSDPNEVALLLHPHADTILELERLPDALAALTENCPDTPTRDDLRSLAKETGELLQQLRANDLLPRSEEVSSYQGETSVRSREVVEPKLTLCQAGGNGQGQLEASSARPESLRYAPTRAASSGSEARVPGQAVGGKIADDAQKVAGLYAEKKRTNWTQANGVAGKISHKIQSLLGMRDAGSRV MSPAQIIRTPHSFPPSFTGTSSSAENSHAQSPQQVLTRAFVASGELNAAFGRTSTASEQDFTSLLGTLQRELERKTLSFPDIAELANQLAEAAKGDQGGHWLGRDEQQTLKGMIDRCKSQLAHTHASDASYDPLAQVCENLKTARLHQSIRQMTGEAHAKVRGVPDLLALIQLDPDVLAEKPVGMTSYVNFGSFICMAKARTAELSEDLRSDPNEVALLLHPHADTILELERLPDALAALTENCPDTPTRDDLRSLAKETGELLQQLRANDLLPRSEEVSSYQGETSVRSREVVEPKLTLCQAGGNGQGQLEASSARPESLRYAPTRAASSGSEARVPGQAVGGKIADDAQKVAGLYAEKKR     A G  G   ++ Q  +   DA  RV MSPAQIIRTPHSFPPSFTGTSSSAENSHAQSPQQVLTRAFVASGELNAAFGRTSTASEQDFTSLLGTLQRELERKTLSFPDIAELANQLAEAAKGDQGGHWLGRDEQQTLKGMIDRCKSQLAHTHASDASYDPLAQVCENLKTARLHQSIRQMTGEAHAKVRGVPDLLALIQLDPDVLAEKPVGMTSYVNFGSFICMAKARTAELSEDLRSDPNEVALLLHPHADTILELERLPDALAALTENCPDTPTRDDLRSLAKETGELLQQLRANDLLPRSEEVSSYQGETSVRSREVVEPKLTLCQAGGNGQGQLEASSARPESLRYAPTRAASSGSEARVPGQAVGGKIADDAQKVAGLYAEKKR---QLAPGKRGSGENQPQDSIPAGDARFRV ``` |
| 0.0 | capillary\_sequence\_10 | ``` C1E_3964 424-753  capillary_sequence_10 1-990 ``` | ```                                                                                                                                                                                                                                                                                                                                                                                                                                        IKGEAALIDFQCDSDGKVVDARHPGRFPVLPQGNEREAFKTVLQELKFRGAETLSKVPVYYVNRNTRGYVIPTHGYVVAGHPNRGRKSGAVLYGVGGDPKRGPVALDEKLLGHLVGRSDSKTSSKLSAPVKAAISALAGASFATREDFYDAYCAVRGDAVDPLERHNEISSIYRLLPLSTMEMWPKKADDYRVARPAAPERDLRAFENLPKDIGRKAQLKKVSNVDSIDLLEAKRQFTLHQLYQDEMLGRNGTGVPSADFKPKVDAQRRDQLVASTPKFQRLPPHTTDKVGNCNTGASSLLQRAVDTYTEKNNLPPEKVTAASIFGIGSS                                                                                                                                                                                                                                                                                                                                                                                                                                        IKGEAALIDFQCDSDGKVVDARHPGRFPVLPQGNEREAFKTVLQELKFRGAETLSKVPVYYVNRNTRGYVIPTHGYVVAGHPNRGRKSGAVLYGVGGDPKRGPVALDEKLLGHLVGRSDSKTSSKLSAPVKAAISALAGASFATREDFYDAYCAVRGDAVDPLERHNEISSIYRLLPLSTMEMWPKKADDYRVARPAAPERDLRAFENLPKDIGRKAQLKKVSNVDSIDLLEAKRQFTLHQLYQDEMLGRNGTGVPSADFKPKVDAQRRDQLVASTPKFQRLPPHTTDKVGNCNTGASSLLQRAVDTYTEKNNLPPEKVTAASIFGIGSS                                                                                                                                                                                                                                                                                                                                                                                                                                        IKGEAALIDFQCDSDGKVVDARHPGRFPVLPQGNEREAFKTVLQELKFRGAETLSKVPVYYVNRNTRGYVIPTHGYVVAGHPNRGRKSGAVLYGVGGDPKRGPVALDEKLLGHLVGRSDSKTSSKLSAPVKAAISALAGASFATREDFYDAYCAVRGDAVDPLERHNEISSIYRLLPLSTMEMWPKKADDYRVARPAAPERDLRAFENLPKDIGRKAQLKKVSNVDSIDLLEAKRQFTLHQLYQDEMLGRNGTGVPSADFKPKVDAQRRDQLVASTPKFQRLPPHTTDKVGNCNTGASSLLQRAVDTYTEKNNLPPEKVTAASIFGIGSS ``` |
| 1e-165 | capillary\_sequence\_90 | ``` C1E_3964 254-539  capillary_sequence_90 3-860 ``` | ```                                                                                                                                                                                                                                                              RSLAKETGELLQQLRANDLLPRSEEVSSYQGETSVRSREVVEPKLTLCQAGGNGQGQLEASSARPESLRYAPTRAASSGSEARVPGQAVGGKIADDAQKVAGLYAEKKRTNWTQANGVAGKISHKIQSLLGMRDAGSRVQAFVAFMADGKGRPGATMLDLGDGWMRATRVIKGEAALIDFQCDSDGKVVDARHPGRFPVLPQGNEREAFKTVLQELKFRGAETLSKVPVYYVNRNTRGYVIPTHGYVVAGHPNRGRKSGAVLYGVGGDPKRGPVALDEKLLGHLVG                                                                                                                                                                                                                                                              RSLAKETGELLQQLRANDLLPRSEEVSSYQGETSVRSREVVEPKLTLCQAGGNGQGQLEASSARPESLRYAPTRAASSGSEARVPGQAVGGKIADDAQKVAGLYAEKKRTNWTQANGVAGKISHKIQSLLGMRDAGSRVQAFVAFMADGKGRPGATMLDLGDGWMRATRVIKGEAALIDFQCDSDGKVVDARHPGRFPVLPQGNEREAFKTVLQELKFRGAETLSKVPVYYVNRNTRGYVIPTHGYVVAGHPNRGRKSGAVLYGVGGDPKRGPVALDEKLLGHLVG                                                                                                                                                                                                                                                              RSLAKETGELLQQLRANDLLPRSEEVSSYQGETSVRSREVVEPKLTLCQAGGNGQGQLEASSARPESLRYAPTRAASSGSEARVPGQAVGGKIADDAQKVAGLYAEKKRTNWTQANGVAGKISHKIQSLLGMRDAGSRVQAFVAFMADGKGRPGATMLDLGDGWMRATRVIKGEAALIDFQCDSDGKVVDARHPGRFPVLPQGNEREAFKTVLQELKFRGAETLSKVPVYYVNRNTRGYVIPTHGYVVAGHPNRGRKSGAVLYGVGGDPKRGPVALDEKLLGHLVG ``` |
| 1e-164 | capillary\_sequence\_92 | ``` C1E_3964 255-539  capillary_sequence_92 3-857 ``` | ```                                                                                                                                                                                                                                                               SLAKETGELLQQLRANDLLPRSEEVSSYQGETSVRSREVVEPKLTLCQAGGNGQGQLEASSARPESLRYAPTRAASSGSEARVPGQAVGGKIADDAQKVAGLYAEKKRTNWTQANGVAGKISHKIQSLLGMRDAGSRVQAFVAFMADGKGRPGATMLDLGDGWMRATRVIKGEAALIDFQCDSDGKVVDARHPGRFPVLPQGNEREAFKTVLQELKFRGAETLSKVPVYYVNRNTRGYVIPTHGYVVAGHPNRGRKSGAVLYGVGGDPKRGPVALDEKLLGHLVG                                                                                                                                                                                                                                                               SLAKETGELLQQLRANDLLPRSEEVSSYQGETSVRSREVVEPKLTLCQAGGNGQGQLEASSARPESLRYAPTRAASSGSEARVPGQAVGGKIADDAQKVAGLYAEKKRTNWTQANGVAGKISHKIQSLLGMRDAGSRVQAFVAFMADGKGRPGATMLDLGDGWMRATRVIKGEAALIDFQCDSDGKVVDARHPGRFPVLPQGNEREAFKTVLQELKFRGAETLSKVPVYYVNRNTRGYVIPTHGYVVAGHPNRGRKSGAVLYGVGGDPKRGPVALDEKLLGHLVG                                                                                                                                                                                                                                                               SLAKETGELLQQLRANDLLPRSEEVSSYQGETSVRSREVVEPKLTLCQAGGNGQGQLEASSARPESLRYAPTRAASSGSEARVPGQAVGGKIADDAQKVAGLYAEKKRTNWTQANGVAGKISHKIQSLLGMRDAGSRVQAFVAFMADGKGRPGATMLDLGDGWMRATRVIKGEAALIDFQCDSDGKVVDARHPGRFPVLPQGNEREAFKTVLQELKFRGAETLSKVPVYYVNRNTRGYVIPTHGYVVAGHPNRGRKSGAVLYGVGGDPKRGPVALDEKLLGHLVG ``` |
| 1e-164 | capillary\_sequence\_44 | ``` C1E_3964 255-539  capillary_sequence_44 3-857 ``` | ```                                                                                                                                                                                                                                                               SLAKETGELLQQLRANDLLPRSEEVSSYQGETSVRSREVVEPKLTLCQAGGNGQGQLEASSARPESLRYAPTRAASSGSEARVPGQAVGGKIADDAQKVAGLYAEKKRTNWTQANGVAGKISHKIQSLLGMRDAGSRVQAFVAFMADGKGRPGATMLDLGDGWMRATRVIKGEAALIDFQCDSDGKVVDARHPGRFPVLPQGNEREAFKTVLQELKFRGAETLSKVPVYYVNRNTRGYVIPTHGYVVAGHPNRGRKSGAVLYGVGGDPKRGPVALDEKLLGHLVG                                                                                                                                                                                                                                                               SLAKETGELLQQLRANDLLPRSEEVSSYQGETSVRSREVVEPKLTLCQAGGNGQGQLEASSARPESLRYAPTRAASSGSEARVPGQAVGGKIADDAQKVAGLYAEKKRTNWTQANGVAGKISHKIQSLLGMRDAGSRVQAFVAFMADGKGRPGATMLDLGDGWMRATRVIKGEAALIDFQCDSDGKVVDARHPGRFPVLPQGNEREAFKTVLQELKFRGAETLSKVPVYYVNRNTRGYVIPTHGYVVAGHPNRGRKSGAVLYGVGGDPKRGPVALDEKLLGHLVG                                                                                                                                                                                                                                                               SLAKETGELLQQLRANDLLPRSEEVSSYQGETSVRSREVVEPKLTLCQAGGNGQGQLEASSARPESLRYAPTRAASSGSEARVPGQAVGGKIADDAQKVAGLYAEKKRTNWTQANGVAGKISHKIQSLLGMRDAGSRVQAFVAFMADGKGRPGATMLDLGDGWMRATRVIKGEAALIDFQCDSDGKVVDARHPGRFPVLPQGNEREAFKTVLQELKFRGAETLSKVPVYYVNRNTRGYVIPTHGYVVAGHPNRGRKSGAVLYGVGGDPKRGPVALDEKLLGHLVG ``` |
| 1e-157 | capillary\_sequence\_93 | ``` C1E_3964 70-345  capillary_sequence_93 2-829 ``` | ```                                                                      RELERKTLSFPDIAELANQLAEAAKGDQGGHWLGRDEQQTLKGMIDRCKSQLAHTHASDASYDPLAQVCENLKTARLHQSIRQMTGEAHAKVRGVPDLLALIQLDPDVLAEKPVGMTSYVNFGSFICMAKARTAELSEDLRSDPNEVALLLHPHADTILELERLPDALAALTENCPDTPTRDDLRSLAKETGELLQQLRANDLLPRSEEVSSYQGETSVRSREVVEPKLTLCQAGGNGQGQLEASSARPESLRYAPTRAASSGSEARVPGQAVGGK                                                                      RELERKTLSFPDIAELANQLAEAAKGDQGGHWLGRDEQQTLKGMIDRCKSQLAHTHASDASYDPLAQVCENLKTARLHQSIRQMTGEAHAKVRGVPDLLALIQLDPDVLAEKPVGMTSYVNFGSFICMAKARTAELSEDLRSDPNEVALLLHPHADTILELERLPDALAALTENCPDTPTRDDLRSLAKETGELLQQLRANDLLPRSEEVSSYQGETSVRSREVVEPKLTLCQAGGNGQGQLEASSARPESLRYAPTRAASSGSEARVPGQAVGGK                                                                      RELERKTLSFPDIAELANQLAEAAKGDQGGHWLGRDEQQTLKGMIDRCKSQLAHTHASDASYDPLAQVCENLKTARLHQSIRQMTGEAHAKVRGVPDLLALIQLDPDVLAEKPVGMTSYVNFGSFICMAKARTAELSEDLRSDPNEVALLLHPHADTILELERLPDALAALTENCPDTPTRDDLRSLAKETGELLQQLRANDLLPRSEEVSSYQGETSVRSREVVEPKLTLCQAGGNGQGQLEASSARPESLRYAPTRAASSGSEARVPGQAVGGK ``` |
| 1e-157 | capillary\_sequence\_91 | ``` C1E_3964 70-345  capillary_sequence_91 2-829 ``` | ```                                                                      RELERKTLSFPDIAELANQLAEAAKGDQGGHWLGRDEQQTLKGMIDRCKSQLAHTHASDASYDPLAQVCENLKTARLHQSIRQMTGEAHAKVRGVPDLLALIQLDPDVLAEKPVGMTSYVNFGSFICMAKARTAELSEDLRSDPNEVALLLHPHADTILELERLPDALAALTENCPDTPTRDDLRSLAKETGELLQQLRANDLLPRSEEVSSYQGETSVRSREVVEPKLTLCQAGGNGQGQLEASSARPESLRYAPTRAASSGSEARVPGQAVGGK                                                                      RELERKTLSFPDIAELANQLAEAAKGDQGGHWLGRDEQQTLKGMIDRCKSQLAHTHASDASYDPLAQVCENLKTARLHQSIRQMTGEAHAKVRGVPDLLALIQLDPDVLAEKPVGMTSYVNFGSFICMAKARTAELSEDLRSDPNEVALLLHPHADTILELERLPDALAALTENCPDTPTRDDLRSLAKETGELLQQLRANDLLPRSEEVSSYQGETSVRSREVVEPKLTLCQAGGNGQGQLEASSARPESLRYAPTRAASSGSEARVPGQAVGGK                                                                      RELERKTLSFPDIAELANQLAEAAKGDQGGHWLGRDEQQTLKGMIDRCKSQLAHTHASDASYDPLAQVCENLKTARLHQSIRQMTGEAHAKVRGVPDLLALIQLDPDVLAEKPVGMTSYVNFGSFICMAKARTAELSEDLRSDPNEVALLLHPHADTILELERLPDALAALTENCPDTPTRDDLRSLAKETGELLQQLRANDLLPRSEEVSSYQGETSVRSREVVEPKLTLCQAGGNGQGQLEASSARPESLRYAPTRAASSGSEARVPGQAVGGK ``` |
| 1e-157 | capillary\_sequence\_47 | ``` C1E_3964 70-345  capillary_sequence_47 2-829 ``` | ```                                                                      RELERKTLSFPDIAELANQLAEAAKGDQGGHWLGRDEQQTLKGMIDRCKSQLAHTHASDASYDPLAQVCENLKTARLHQSIRQMTGEAHAKVRGVPDLLALIQLDPDVLAEKPVGMTSYVNFGSFICMAKARTAELSEDLRSDPNEVALLLHPHADTILELERLPDALAALTENCPDTPTRDDLRSLAKETGELLQQLRANDLLPRSEEVSSYQGETSVRSREVVEPKLTLCQAGGNGQGQLEASSARPESLRYAPTRAASSGSEARVPGQAVGGK                                                                      RELERKTLSFPDIAELANQLAEAAKGDQGGHWLGRDEQQTLKGMIDRCKSQLAHTHASDASYDPLAQVCENLKTARLHQSIRQMTGEAHAKVRGVPDLLALIQLDPDVLAEKPVGMTSYVNFGSFICMAKARTAELSEDLRSDPNEVALLLHPHADTILELERLPDALAALTENCPDTPTRDDLRSLAKETGELLQQLRANDLLPRSEEVSSYQGETSVRSREVVEPKLTLCQAGGNGQGQLEASSARPESLRYAPTRAASSGSEARVPGQAVGGK                                                                      RELERKTLSFPDIAELANQLAEAAKGDQGGHWLGRDEQQTLKGMIDRCKSQLAHTHASDASYDPLAQVCENLKTARLHQSIRQMTGEAHAKVRGVPDLLALIQLDPDVLAEKPVGMTSYVNFGSFICMAKARTAELSEDLRSDPNEVALLLHPHADTILELERLPDALAALTENCPDTPTRDDLRSLAKETGELLQQLRANDLLPRSEEVSSYQGETSVRSREVVEPKLTLCQAGGNGQGQLEASSARPESLRYAPTRAASSGSEARVPGQAVGGK ``` |
| 1e-123 | capillary\_sequence\_11 | ``` C1E_3964 1-218  capillary_sequence_11 319-972 ``` | ``` MSPAQIIRTPHSFPPSFTGTSSSAENSHAQSPQQVLTRAFVASGELNAAFGRTSTASEQDFTSLLGTLQRELERKTLSFPDIAELANQLAEAAKGDQGGHWLGRDEQQTLKGMIDRCKSQLAHTHASDASYDPLAQVCENLKTARLHQSIRQMTGEAHAKVRGVPDLLALIQLDPDVLAEKPVGMTSYVNFGSFICMAKARTAELSEDLRSDPNEVAL MSPAQIIRTPHSFPPSFTGTSSSAENSHAQSPQQVLTRAFVASGELNAAFGRTSTASEQDFTSLLGTLQRELERKTLSFPDIAELANQLAEAAKGDQGGHWLGRDEQQTLKGMIDRCKSQLAHTHASDASYDPLAQVCENLKTARLHQSIRQMTGEAHAKVRGVPDLLALIQLDPDVLAEKPVGMTSYVNFGSFICMAKARTAELSEDLRSDPNEVAL MSPAQIIRTPHSFPPSFTGTSSSAENSHAQSPQQVLTRAFVASGELNAAFGRTSTASEQDFTSLLGTLQRELERKTLSFPDIAELANQLAEAAKGDQGGHWLGRDEQQTLKGMIDRCKSQLAHTHASDASYDPLAQVCENLKTARLHQSIRQMTGEAHAKVRGVPDLLALIQLDPDVLAEKPVGMTSYVNFGSFICMAKARTAELSEDLRSDPNEVAL ``` |

---

### **C1E\_3975** 955:85214..86053 Operon 56 Gene 1 gi|28870262|ref|NP\_792881.1| type III effector HopAB2

|  |  |  |  |
| --- | --- | --- | --- |
| 1e-162 | capillary\_sequence\_22 | ``` C1E_3975 1-279  capillary_sequence_22 22-858 ``` | ``` MRNRGNNQAAAALQGLVQQGVNLEDLRTALERHLLRHQPIPLDIAYALQSVGIPPSVDTAESLVESPLMDLSVALHRVLGPRPVSAPPRPAVPVHPPAASRRPDGARSSALRVIPEREDYENNVAYGMRLLNLNPGVGVRRVVAAFITDPADRPAVVDDIRAARDPITSQFNQLRTVSKAVVESQNPPFRDAEHHHPDDATHCLFGEPLSLENPDQQVIGLAGNPTDTSELYSQQGNKDLVFMDMKKLAQFLAGKPEHPMNRQPLDARTIANYAFRIVP MRNRGNNQAAAALQGLVQQGVNLEDLRTALERHLLRHQPIPLDIAYALQSVGIPPSVDTAESLVESPLMDLSVALHRVLGPRPVSAPPRPAVPVHPPAASRRPDGARSSALRVIPEREDYENNVAYGMRLLNLNPGVGVRRVVAAFITDPADRPAVVDDIRAARDPITSQFNQLRTVSKAVVESQNPPFRDAEHHHPDDATHCLFGEPLSLENPDQQVIGLAGNPTDTSELYSQQGNKDLVFMDMKKLAQFLAGKPEHPMNRQPLDARTIANYAFRIVP MRNRGNNQAAAALQGLVQQGVNLEDLRTALERHLLRHQPIPLDIAYALQSVGIPPSVDTAESLVESPLMDLSVALHRVLGPRPVSAPPRPAVPVHPPAASRRPDGARSSALRVIPEREDYENNVAYGMRLLNLNPGVGVRRVVAAFITDPADRPAVVDDIRAARDPITSQFNQLRTVSKAVVESQNPPFRDAEHHHPDDATHCLFGEPLSLENPDQQVIGLAGNPTDTSELYSQQGNKDLVFMDMKKLAQFLAGKPEHPMNRQPLDARTIANYAFRIVP ``` |
| 1e-145 | capillary\_sequence\_43 | ``` C1E_3975 1-252  capillary_sequence_43 3-758 ``` | ``` MRNRGNNQAAAALQGLVQQGVNLEDLRTALERHLLRHQPIPLDIAYALQSVGIPPSVDTAESLVESPLMDLSVALHRVLGPRPVSAPPRPAVPVHPPAASRRPDGARSSALRVIPEREDYENNVAYGMRLLNLNPGVGVRRVVAAFITDPADRPAVVDDIRAARDPITSQFNQLRTVSKAVVESQNPPFRDAEHHHPDDATHCLFGEPLSLENPDQQVIGLAGNPTDTSELYSQQGNKDLVFMDMKKLAQFL MRNRGNNQAAAALQGLVQQGVNLEDLRTALERHLLRHQPIPLDIAYALQSVGIPPSVDTAESLVESPLMDLSVALHRVLGPRPVSAPPRPAVPVHPPAASRRPDGARSSALRVIPEREDYENNVAYGMRLLNLNPGVGVRRVVAAFITDPADRPAVVDDIRAARDPITSQFNQLRTVSKAVVESQNPPFRDAEHHHPDDATHCLFGEPLSLENPDQQVIGLAGNPTDTSELYSQQGNKDLVFMDMKKLAQFL MRNRGNNQAAAALQGLVQQGVNLEDLRTALERHLLRHQPIPLDIAYALQSVGIPPSVDTAESLVESPLMDLSVALHRVLGPRPVSAPPRPAVPVHPPAASRRPDGARSSALRVIPEREDYENNVAYGMRLLNLNPGVGVRRVVAAFITDPADRPAVVDDIRAARDPITSQFNQLRTVSKAVVESQNPPFRDAEHHHPDDATHCLFGEPLSLENPDQQVIGLAGNPTDTSELYSQQGNKDLVFMDMKKLAQFL ``` |

---

### **C1E\_4217** 1039:95695..96669 ABC-type nitrate/sulfonate/bicarbonate transport systems, periplasmic components Operon 48 Gene 1 COG0715 ABC-type nitrate/sulfonate/bicarbonate transport systems, periplasmic components


---

### **C1E\_4987** 1087:57088..58188 N-Dimethylarginine dimethylaminohydrolase Operon 38 Gene 3 COG1834 N-Dimethylarginine dimethylaminohydrolase

|  |  |  |  |
| --- | --- | --- | --- |
| 1e-174 | capillary\_sequence\_36 | ``` C1E_4987 1-286  capillary_sequence_36 1028-1885 ``` | ``` MSYQKAEPAYFTTTQSPVEVYTEWDPLEEVVVGIMDDIRVPDWDLGLKAIIPKESSDFFMTYSGRRFPEELLVKARQEVNTLARILETEGVRVKRPNESNHHQPIMTPHFTTGGTFYSAMPRDCLFAIGKKIIEVPMAWRSRYFETFAFRDLLNDYFSQGAEWIAAPKPMLKDDVWQPNYDFEQELPFRSIITEAEPLFDAADFMKMGRDIIGQRSHVTNNKGIEWLRRTLGPDYRVHIYEFEEPGPMHIDTTILPLAPGRVLINKDWVPQIPDIFKDWEILNPPP MSYQKAEPAYFTTTQSPVEVYTEWDPLEEVVVGIMDDIRVPDWDLGLKAIIPKESSDFFMTYSGRRFPEELLVKARQEVNTLARILETEGVRVKRPNESNHHQPIMTPHFTTGGTFYSAMPRDCLFAIGKKIIEVPMAWRSRYFETFAFRDLLNDYFSQGAEWIAAPKPMLKDDVWQPNYDFEQELPFRSIITEAEPLFDAADFMKMGRDIIGQRSHVTNNKGIEWLRRTLGPDYRVHIYEFEEPGPMHIDTTILPLAPGRVLINKDWVPQIPDIFKDWEILNPPP MSYQKAEPAYFTTTQSPVEVYTEWDPLEEVVVGIMDDIRVPDWDLGLKAIIPKESSDFFMTYSGRRFPEELLVKARQEVNTLARILETEGVRVKRPNESNHHQPIMTPHFTTGGTFYSAMPRDCLFAIGKKIIEVPMAWRSRYFETFAFRDLLNDYFSQGAEWIAAPKPMLKDDVWQPNYDFEQELPFRSIITEAEPLFDAADFMKMGRDIIGQRSHVTNNKGIEWLRRTLGPDYRVHIYEFEEPGPMHIDTTILPLAPGRVLINKDWVPQIPDIFKDWEILNPPP ``` |
| 1e-168 | capillary\_sequence\_49 | ``` C1E_4987 1-278  capillary_sequence_49 2-835 ``` | ``` MSYQKAEPAYFTTTQSPVEVYTEWDPLEEVVVGIMDDIRVPDWDLGLKAIIPKESSDFFMTYSGRRFPEELLVKARQEVNTLARILETEGVRVKRPNESNHHQPIMTPHFTTGGTFYSAMPRDCLFAIGKKIIEVPMAWRSRYFETFAFRDLLNDYFSQGAEWIAAPKPMLKDDVWQPNYDFEQELPFRSIITEAEPLFDAADFMKMGRDIIGQRSHVTNNKGIEWLRRTLGPDYRVHIYEFEEPGPMHIDTTILPLAPGRVLINKDWVPQIPDIFKD MSYQKAEPAYFTTTQSPVEVYTEWDPLEEVVVGIMDDIRVPDWDLGLKAIIPKESSDFFMTYSGRRFPEELLVKARQEVNTLARILETEGVRVKRPNESNHHQPIMTPHFTTGGTFYSAMPRDCLFAIGKKIIEVPMAWRSRYFETFAFRDLLNDYFSQGAEWIAAPKPMLKDDVWQPNYDFEQELPFRSIITEAEPLFDAADFMKMGRDIIGQRSHVTNNKGIEWLRRTLGPDYRVHIYEFEEPGPMHIDTTILPLAPGRVLINKDWVPQIPDIFKD MSYQKAEPAYFTTTQSPVEVYTEWDPLEEVVVGIMDDIRVPDWDLGLKAIIPKESSDFFMTYSGRRFPEELLVKARQEVNTLARILETEGVRVKRPNESNHHQPIMTPHFTTGGTFYSAMPRDCLFAIGKKIIEVPMAWRSRYFETFAFRDLLNDYFSQGAEWIAAPKPMLKDDVWQPNYDFEQELPFRSIITEAEPLFDAADFMKMGRDIIGQRSHVTNNKGIEWLRRTLGPDYRVHIYEFEEPGPMHIDTTILPLAPGRVLINKDWVPQIPDIFKD ``` |

---

### **C1E\_4990** 1087:59133..60425 Permeases of the major facilitator superfamily Operon 40 Gene 1 COG0477 Permeases of the major facilitator superfamily

|  |  |  |  |
| --- | --- | --- | --- |
| 0.0 | capillary\_sequence\_49 | ``` C1E_4990 1-430  capillary_sequence_49 1783-3072 ``` | ``` MALSNRPEIIDTADGHSLPNSDNSDLESNWQVRFWMIFTGQTLSLIGSGLTQFVLLWWITDTTGSLAALATAGVVALLPQALISPLGGIFADRYSRRVLMIATDMISALCMSILIVLFLTERVELWHVYWMMFVRSAMQAFQTPAASASVAMLVPRSFLTRAAGLSQAMQGITLVAAAPLGALAISMIPLGWALSIDVVTALLGCLPLLRYRIPQAFNSNHTGLSTLRSEFRDGLHLIWSHPGLRHLYALMGGVVLMITPSFTLVPLLVKEHFGGGAPHVAFIDAMAGAGMLIGAVAVALFAPRQQVKWILWGFATSCFALALTGLMPADRFNIAAVCWLISGMSFILGDAPMTALLQGTIPNHLQGRGLSLLNMVMGLAAPLGLALTTPLGELIGVRWLFVFTGVLGGLICLMGFFSSAVRRLEDGTHY MALSNRPEIIDTADGHSLPNSDNSDLESNWQVRFWMIFTGQTLSLIGSGLTQFVLLWWITDTTGSLAALATAGVVALLPQALISPLGGIFADRYSRRVLMIATDMISALCMSILIVLFLTERVELWHVYWMMFVRSAMQAFQTPAASASVAMLVPRSFLTRAAGLSQAMQGITLVAAAPLGALAISMIPLGWALSIDVVTALLGCLPLLRYRIPQAFNSNHTGLSTLRSEFRDGLHLIWSHPGLRHLYALMGGVVLMITPSFTLVPLLVKEHFGGGAPHVAFIDAMAGAGMLIGAVAVALFAPRQQVKWILWGFATSCFALALTGLMPADRFNIAAVCWLISGMSFILGDAPMTALLQGTIPNHLQGRGLSLLNMVMGLAAPLGLALTTPLGELIGVRWLFVFTGVLGGLICLMGFFSSAVRRLEDGTHY MALSNRPEIIDTADGHSLPNSDNSDLESNWQVRFWMIFTGQTLSLIGSGLTQFVLLWWITDTTGSLAALATAGVVALLPQALISPLGGIFADRYSRRVLMIATDMISALCMSILIVLFLTERVELWHVYWMMFVRSAMQAFQTPAASASVAMLVPRSFLTRAAGLSQAMQGITLVAAAPLGALAISMIPLGWALSIDVVTALLGCLPLLRYRIPQAFNSNHTGLSTLRSEFRDGLHLIWSHPGLRHLYALMGGVVLMITPSFTLVPLLVKEHFGGGAPHVAFIDAMAGAGMLIGAVAVALFAPRQQVKWILWGFATSCFALALTGLMPADRFNIAAVCWLISGMSFILGDAPMTALLQGTIPNHLQGRGLSLLNMVMGLAAPLGLALTTPLGELIGVRWLFVFTGVLGGLICLMGFFSSAVRRLEDGTHY ``` |
| 0.0 | capillary\_sequence\_37 | ``` C1E_4990 1-430  capillary_sequence_37 243-1532 ``` | ``` MALSNRPEIIDTADGHSLPNSDNSDLESNWQVRFWMIFTGQTLSLIGSGLTQFVLLWWITDTTGSLAALATAGVVALLPQALISPLGGIFADRYSRRVLMIATDMISALCMSILIVLFLTERVELWHVYWMMFVRSAMQAFQTPAASASVAMLVPRSFLTRAAGLSQAMQGITLVAAAPLGALAISMIPLGWALSIDVVTALLGCLPLLRYRIPQAFNSNHTGLSTLRSEFRDGLHLIWSHPGLRHLYALMGGVVLMITPSFTLVPLLVKEHFGGGAPHVAFIDAMAGAGMLIGAVAVALFAPRQQVKWILWGFATSCFALALTGLMPADRFNIAAVCWLISGMSFILGDAPMTALLQGTIPNHLQGRGLSLLNMVMGLAAPLGLALTTPLGELIGVRWLFVFTGVLGGLICLMGFFSSAVRRLEDGTHY MALSNRPEIIDTADGHSLPNSDNSDLESNWQVRFWMIFTGQTLSLIGSGLTQFVLLWWITDTTGSLAALATAGVVALLPQALISPLGGIFADRYSRRVLMIATDMISALCMSILIVLFLTERVELWHVYWMMFVRSAMQAFQTPAASASVAMLVPRSFLTRAAGLSQAMQGITLVAAAPLGALAISMIPLGWALSIDVVTALLGCLPLLRYRIPQAFNSNHTGLSTLRSEFRDGLHLIWSHPGLRHLYALMGGVVLMITPSFTLVPLLVKEHFGGGAPHVAFIDAMAGAGMLIGAVAVALFAPRQQVKWILWGFATSCFALALTGLMPADRFNIAAVCWLISGMSFILGDAPMTALLQGTIPNHLQGRGLSLLNMVMGLAAPLGLALTTPLGELIGVRWLFVFTGVLGGLICLMGFFSSAVRRLEDGTHY MALSNRPEIIDTADGHSLPNSDNSDLESNWQVRFWMIFTGQTLSLIGSGLTQFVLLWWITDTTGSLAALATAGVVALLPQALISPLGGIFADRYSRRVLMIATDMISALCMSILIVLFLTERVELWHVYWMMFVRSAMQAFQTPAASASVAMLVPRSFLTRAAGLSQAMQGITLVAAAPLGALAISMIPLGWALSIDVVTALLGCLPLLRYRIPQAFNSNHTGLSTLRSEFRDGLHLIWSHPGLRHLYALMGGVVLMITPSFTLVPLLVKEHFGGGAPHVAFIDAMAGAGMLIGAVAVALFAPRQQVKWILWGFATSCFALALTGLMPADRFNIAAVCWLISGMSFILGDAPMTALLQGTIPNHLQGRGLSLLNMVMGLAAPLGLALTTPLGELIGVRWLFVFTGVLGGLICLMGFFSSAVRRLEDGTHY ``` |
| 0.0 | capillary\_sequence\_80 | ``` C1E_4990 87-415  capillary_sequence_80 2-988 ``` | ```                                                                                       GGIFADRYSRRVLMIATDMISALCMSILIVLFLTERVELWHVYWMMFVRSAMQAFQTPAASASVAMLVPRSFLTRAAGLSQAMQGITLVAAAPLGALAISMIPLGWALSIDVVTALLGCLPLLRYRIPQAFNSNHTGLSTLRSEFRDGLHLIWSHPGLRHLYALMGGVVLMITPSFTLVPLLVKEHFGGGAPHVAFIDAMAGAGMLIGAVAVALFAPRQQVKWILWGFATSCFALALTGLMPADRFNIAAVCWLISGMSFILGDAPMTALLQGTIPNHLQGRGLSLLNMVMGLAAPLGLALTTPLGELIGVRWLFVFTGVLGGLICLMG                                                                                       GGIFADRYSRRVLMIATDMISALCMSILIVLFLTERVELWHVYWMMFVRSAMQAFQTPAASASVAMLVPRSFLTRAAGLSQAMQGITLVAAAPLGALAISMIPLGWALSIDVVTALLGCLPLLRYRIPQAFNSNHTGLSTLRSEFRDGLHLIWSHPGLRHLYALMGGVVLMITPSFTLVPLLVKEHFGGGAPHVAFIDAMAGAGMLIGAVAVALFAPRQQVKWILWGFATSCFALALTGLMPADRFNIAAVCWLISGMSFILGDAPMTALLQGTIPNHLQGRGLSLLNMVMGLAAPLGLALTTPLGELIGVRWLFVFTGVLGGLICLMG                                                                                       GGIFADRYSRRVLMIATDMISALCMSILIVLFLTERVELWHVYWMMFVRSAMQAFQTPAASASVAMLVPRSFLTRAAGLSQAMQGITLVAAAPLGALAISMIPLGWALSIDVVTALLGCLPLLRYRIPQAFNSNHTGLSTLRSEFRDGLHLIWSHPGLRHLYALMGGVVLMITPSFTLVPLLVKEHFGGGAPHVAFIDAMAGAGMLIGAVAVALFAPRQQVKWILWGFATSCFALALTGLMPADRFNIAAVCWLISGMSFILGDAPMTALLQGTIPNHLQGRGLSLLNMVMGLAAPLGLALTTPLGELIGVRWLFVFTGVLGGLICLMG ``` |
| 0.0 | capillary\_sequence\_18 | ``` C1E_4990 87-415  capillary_sequence_18 1-987 ``` | ```                                                                                       GGIFADRYSRRVLMIATDMISALCMSILIVLFLTERVELWHVYWMMFVRSAMQAFQTPAASASVAMLVPRSFLTRAAGLSQAMQGITLVAAAPLGALAISMIPLGWALSIDVVTALLGCLPLLRYRIPQAFNSNHTGLSTLRSEFRDGLHLIWSHPGLRHLYALMGGVVLMITPSFTLVPLLVKEHFGGGAPHVAFIDAMAGAGMLIGAVAVALFAPRQQVKWILWGFATSCFALALTGLMPADRFNIAAVCWLISGMSFILGDAPMTALLQGTIPNHLQGRGLSLLNMVMGLAAPLGLALTTPLGELIGVRWLFVFTGVLGGLICLMG                                                                                       GGIFADRYSRRVLMIATDMISALCMSILIVLFLTERVELWHVYWMMFVRSAMQAFQTPAASASVAMLVPRSFLTRAAGLSQAMQGITLVAAAPLGALAISMIPLGWALSIDVVTALLGCLPLLRYRIPQAFNSNHTGLSTLRSEFRDGLHLIWSHPGLRHLYALMGGVVLMITPSFTLVPLLVKEHFGGGAPHVAFIDAMAGAGMLIGAVAVALFAPRQQVKWILWGFATSCFALALTGLMPADRFNIAAVCWLISGMSFILGDAPMTALLQGTIPNHLQGRGLSLLNMVMGLAAPLGLALTTPLGELIGVRWLFVFTGVLGGLICLMG                                                                                       GGIFADRYSRRVLMIATDMISALCMSILIVLFLTERVELWHVYWMMFVRSAMQAFQTPAASASVAMLVPRSFLTRAAGLSQAMQGITLVAAAPLGALAISMIPLGWALSIDVVTALLGCLPLLRYRIPQAFNSNHTGLSTLRSEFRDGLHLIWSHPGLRHLYALMGGVVLMITPSFTLVPLLVKEHFGGGAPHVAFIDAMAGAGMLIGAVAVALFAPRQQVKWILWGFATSCFALALTGLMPADRFNIAAVCWLISGMSFILGDAPMTALLQGTIPNHLQGRGLSLLNMVMGLAAPLGLALTTPLGELIGVRWLFVFTGVLGGLICLMG ``` |
| 0.0 | capillary\_sequence\_78 | ``` C1E_4990 97-415  capillary_sequence_78 1-957 ``` | ```                                                                                                 RVLMIATDMISALCMSILIVLFLTERVELWHVYWMMFVRSAMQAFQTPAASASVAMLVPRSFLTRAAGLSQAMQGITLVAAAPLGALAISMIPLGWALSIDVVTALLGCLPLLRYRIPQAFNSNHTGLSTLRSEFRDGLHLIWSHPGLRHLYALMGGVVLMITPSFTLVPLLVKEHFGGGAPHVAFIDAMAGAGMLIGAVAVALFAPRQQVKWILWGFATSCFALALTGLMPADRFNIAAVCWLISGMSFILGDAPMTALLQGTIPNHLQGRGLSLLNMVMGLAAPLGLALTTPLGELIGVRWLFVFTGVLGGLICLMG                                                                                                 RVLMIATDMISALCMSIL VLFLTERVELWHVYWMMFVRSAMQAFQTPAASASVAMLVPRSFLTRAAGLSQAMQGITLVAAAPLGALAISMIPLGWALSIDVVTALLGCLPLLRYRIPQAFNSNHTGLSTLRSEFRDGLHLIWSHPGLRHLYALMGGVVLMITPSFTLVPLLVKEHFGGGAPHVAFIDAMAGAGMLIGAVAVALFAPRQQVKWILWGFATSCFALALTGLMPADRFNIAAVCWLISGMSFILGDAPMTALLQGTIPNHLQGRGLSLLNMVMGLAAPLGLALTTPLGELIGVRWLFVFTGVLGGLICLMG                                                                                                 RVLMIATDMISALCMSILXVLFLTERVELWHVYWMMFVRSAMQAFQTPAASASVAMLVPRSFLTRAAGLSQAMQGITLVAAAPLGALAISMIPLGWALSIDVVTALLGCLPLLRYRIPQAFNSNHTGLSTLRSEFRDGLHLIWSHPGLRHLYALMGGVVLMITPSFTLVPLLVKEHFGGGAPHVAFIDAMAGAGMLIGAVAVALFAPRQQVKWILWGFATSCFALALTGLMPADRFNIAAVCWLISGMSFILGDAPMTALLQGTIPNHLQGRGLSLLNMVMGLAAPLGLALTTPLGELIGVRWLFVFTGVLGGLICLMG ``` |
| 1e-144 | capillary\_sequence\_81 | ``` C1E_4990 1-252  capillary_sequence_81 215-970 ``` | ``` MALSNRPEIIDTADGHSLPNSDNSDLESNWQVRFWMIFTGQTLSLIGSGLTQFVLLWWITDTTGSLAALATAGVVALLPQALISPLGGIFADRYSRRVLMIATDMISALCMSILIVLFLTERVELWHVYWMMFVRSAMQAFQTPAASASVAMLVPRSFLTRAAGLSQAMQGITLVAAAPLGALAISMIPLGWALSIDVVTALLGCLPLLRYRIPQAFNSNHTGLSTLRSEFRDGLHLIWSHPGLRHLYALMG MALSNRPEIIDTADGHSLPNSDNSDLESNWQVRFWMIFTGQTLSLIGSGLTQFVLLWWITDTTGSLAALATAGVVALLPQALISPLGGIFADRYSRRVLMIATDMISALCMSILIVLFLTERVELWHVYWMMFVRSAMQAFQTPAASASVAMLVPRSFLTRAAGLSQAMQGITLVAAAPLGALAISMIPLGWALSIDVVTALLGCLPLLRYRIPQAFNSNHTGLSTLRSEFRDGLHLIWSHPGLRHLYALMG MALSNRPEIIDTADGHSLPNSDNSDLESNWQVRFWMIFTGQTLSLIGSGLTQFVLLWWITDTTGSLAALATAGVVALLPQALISPLGGIFADRYSRRVLMIATDMISALCMSILIVLFLTERVELWHVYWMMFVRSAMQAFQTPAASASVAMLVPRSFLTRAAGLSQAMQGITLVAAAPLGALAISMIPLGWALSIDVVTALLGCLPLLRYRIPQAFNSNHTGLSTLRSEFRDGLHLIWSHPGLRHLYALMG ``` |
| 1e-144 | capillary\_sequence\_19 | ``` C1E_4990 1-252  capillary_sequence_19 215-970 ``` | ``` MALSNRPEIIDTADGHSLPNSDNSDLESNWQVRFWMIFTGQTLSLIGSGLTQFVLLWWITDTTGSLAALATAGVVALLPQALISPLGGIFADRYSRRVLMIATDMISALCMSILIVLFLTERVELWHVYWMMFVRSAMQAFQTPAASASVAMLVPRSFLTRAAGLSQAMQGITLVAAAPLGALAISMIPLGWALSIDVVTALLGCLPLLRYRIPQAFNSNHTGLSTLRSEFRDGLHLIWSHPGLRHLYALMG MALSNRPEIIDTADGHSLPNSDNSDLESNWQVRFWMIFTGQTLSLIGSGLTQFVLLWWITDTTGSLAALATAGVVALLPQALISPLGGIFADRYSRRVLMIATDMISALCMSILIVLFLTERVELWHVYWMMFVRSAMQAFQTPAASASVAMLVPRSFLTRAAGLSQAMQGITLVAAAPLGALAISMIPLGWALSIDVVTALLGCLPLLRYRIPQAFNSNHTGLSTLRSEFRDGLHLIWSHPGLRHLYALMG MALSNRPEIIDTADGHSLPNSDNSDLESNWQVRFWMIFTGQTLSLIGSGLTQFVLLWWITDTTGSLAALATAGVVALLPQALISPLGGIFADRYSRRVLMIATDMISALCMSILIVLFLTERVELWHVYWMMFVRSAMQAFQTPAASASVAMLVPRSFLTRAAGLSQAMQGITLVAAAPLGALAISMIPLGWALSIDVVTALLGCLPLLRYRIPQAFNSNHTGLSTLRSEFRDGLHLIWSHPGLRHLYALMG ``` |
| 1e-140 | capillary\_sequence\_79 | ``` C1E_4990 1-254  capillary_sequence_79 224-985 ``` | ``` MALSNRPEIIDTADGHSLPNSDNSDLESNWQVRFWMIFTGQTLSLIGSGLTQFVLLWWITDTTGSLAALATAGVVALLPQALISPLGGIFADRYSRRVLMIATDMISALCMSILIVLFLTERVELWHVYWMMFVRSAMQAFQTPAASASVAMLVPRSFLTRAAGLSQAMQGITLVAAAPLGALAISMIPLGWALSIDVVTALLGCLPLLRYRIPQAFNSNHTGLSTLRSEFRDGLHLIWSHPGLRHLYALMGGV MALSNRPEIIDTADGHSLPNSDNSDLESNWQVRFWMIFTGQTLSLIGSGLTQFVLLWWITDTTGSLAALATAGVVALLPQALISPLGGIFADRYSRRVLMIATDMISALCMSILIVLFLTERVELWHVYWMMFVRSAMQAFQTPAASASVAMLVPRSFLTRAAGLSQAMQGITLVAAAPLGALAISMIPLGWALSIDVVTALLGCLPLLRYRIPQAFNSNHTGLSTLRSEFRDGLHLIWSHPGLR      GGV MALSNRPEIIDTADGHSLPNSDNSDLESNWQVRFWMIFTGQTLSLIGSGLTQFVLLWWITDTTGSLAALATAGVVALLPQALISPLGGIFADRYSRRVLMIATDMISALCMSILIVLFLTERVELWHVYWMMFVRSAMQAFQTPAASASVAMLVPRSFLTRAAGLSQAMQGITLVAAAPLGALAISMIPLGWALSIDVVTALLGCLPLLRYRIPQAFNSNHTGLSTLRSEFRDGLHLIWSHPGLRXSMR*WGGV ``` |

---

### **C1E\_5009** 1087:72050..72664 Operon 49 Gene 1 gi|7677395|gb|AAF67149.1|AF231452\_2 ORF2

|  |  |  |  |
| --- | --- | --- | --- |
| 1e-117 | capillary\_sequence\_27 | ``` C1E_5009 1-204  capillary_sequence_27 649-1260 ``` | ``` MGNICSSGGVSRTYSPPASPVYGSGVSSPSRFVGQYTLTSIHQLSSEERENFLDAHDPMRVYDLNSETSVYRTTPREYVRNGYATGNPNSGATIALHEELQESPYAQHIGARPDQADAYRPRTAHASSLNTPSLNVMAGQGALSALRSYARSDHVTTKMRLGDFLDQGGKVYSDTSAMSAGGDSVEALIVTLPKGRKVPVKILD MGNICSSGGVSRTYSPPASPVYGSGVSSPSRFVGQYTLTSIHQLSSEERENFLDAHDPMRVYDLNSETSVYRTTPREYVRNGYATGNPNSGATIALHEELQESPYAQHIGARPDQADAYRPRTAHASSLNTPSLNVMAGQGALSALRSYARSDHVTTKMRLGDFLDQGGKVYSDTSAMSAGGDSVEALIVTLPKGRKVPVKILD MGNICSSGGVSRTYSPPASPVYGSGVSSPSRFVGQYTLTSIHQLSSEERENFLDAHDPMRVYDLNSETSVYRTTPREYVRNGYATGNPNSGATIALHEELQESPYAQHIGARPDQADAYRPRTAHASSLNTPSLNVMAGQGALSALRSYARSDHVTTKMRLGDFLDQGGKVYSDTSAMSAGGDSVEALIVTLPKGRKVPVKILD ``` |

---

### **C1E\_5021** 1087:77437..78576 Operon 58 Gene 1 gi|213972199|ref|ZP\_03400282.1| type III effector hopT1-1

|  |  |  |  |
| --- | --- | --- | --- |
| 0.0 | capillary\_sequence\_32 | ``` C1E_5021 1-379  capillary_sequence_32 1805-2941 ``` | ``` MMKTVSNHSIPSTNLVVDAGAETLAQKSQPVSSEIQRNSKIEKAVIEHIADHPAAKMTISALVDTLTDVFVRAHGEVKGWAEIVQAVSRPHDSNRHGSGVLSPRFDVMGSVGWNAAAIRATSRVGTLREKGTLFTNLMLSNNFKHLLKRVVSDPALQQKLDGGVDLNYLKACEGDLYVMSGWAARASESREQIGKARYETASNLSQTLISARELAFHRHNPVNHPSAQTKVGFDKGLPEESDLQVLRGHGSSVWSVKPGSDFAKRAEVSGKPIIAGPSGTASRMVAVARFLAPACLKSLGIESEQNLKELVRYACYAYFGQDSHHSMLEVNLGVASHGMPEQWDDTLYNEPFSNSIKGRGFGIDNLAQRQVVRQAAQKS MMKTVSNHSIPSTNLVVDAGAETLAQKSQPVSSEIQRNSKIEKAVIEHIADHPAAKMTISALVDTLTDVFVRAHGEVKGWAEIVQAVSRPHDSNRHGSGVLSPRFDVMGSVGWNAAAIRATSRVGTLREKGTLFTNLMLSNNFKHLLKRVVSDPALQQKLDGGVDLNYLKACEGDLYVMSGWAARASESREQIGKARYETASNLSQTLISARELAFHRHNPVNHPSAQTKVGFDKGLPEESDLQVLRGHGSSVWSVKPGSDFAKRAEVSGKPIIAGPSGTASRMVAVARFLAPACLKSLGIESEQNLKELVRYACYAYFGQDSHHSMLEVNLGVASHGMPEQWDDTLYNEPFSNSIKGRGFGIDNLAQRQVVRQAAQKS MMKTVSNHSIPSTNLVVDAGAETLAQKSQPVSSEIQRNSKIEKAVIEHIADHPAAKMTISALVDTLTDVFVRAHGEVKGWAEIVQAVSRPHDSNRHGSGVLSPRFDVMGSVGWNAAAIRATSRVGTLREKGTLFTNLMLSNNFKHLLKRVVSDPALQQKLDGGVDLNYLKACEGDLYVMSGWAARASESREQIGKARYETASNLSQTLISARELAFHRHNPVNHPSAQTKVGFDKGLPEESDLQVLRGHGSSVWSVKPGSDFAKRAEVSGKPIIAGPSGTASRMVAVARFLAPACLKSLGIESEQNLKELVRYACYAYFGQDSHHSMLEVNLGVASHGMPEQWDDTLYNEPFSNSIKGRGFGIDNLAQRQVVRQAAQKS ``` |

---

### **C1E\_5022** 1087:78582..79433 Operon 58 Gene 2 gi|29171493|ref|NP\_808677.1| type III effector HopO1-1

|  |  |  |  |
| --- | --- | --- | --- |
| 1e-167 | capillary\_sequence\_32 | ``` C1E_5022 1-283  capillary_sequence_32 948-1796 ``` | ``` MGNICGTSGSNHVYSPPISPQHASGSSTPVPSASGTMLSLSHEQILSQNYASNIKGKYRTNPRKGPSPRLSDTLMKQALSSVITQEKKRLKSQPKSIAQDIQPPNSMIKNALDEKDSHPFGDCFSDDEFLAIHLYTSCLYRPINHHLRYAPKNDVAPVVEAMKSGLAKLAQYPDYQVSGQLHRGIKQKMDDGEVMSRFKPGNTYRDDAFMSTSTRMDVTEEFTSDVTLHLQSSSAVNIGPFSKNPYEDEALIPPLTPFKVTSLHKQDDRWHVHLNEIAESSDE MGNICGTSGSNHVYSPPISPQHASGSSTPVPSASGTMLSLSHEQILSQNYASNIKGKYRTNPRKGPSPRLSDTLMKQALSSVITQEKKRLKSQPKSIAQDIQPPNSMIKNALDEKDSHPFGDCFSDDEFLAIHLYTSCLYRPINHHLRYAPKNDVAPVVEAMKSGLAKLAQYPDYQVSGQLHRGIKQKMDDGEVMSRFKPGNTYRDDAFMSTSTRMDVTEEFTSDVTLHLQSSSAVNIGPFSKNPYEDEALIPPLTPFKVTSLHKQDDRWHVHLNEIAESSDE MGNICGTSGSNHVYSPPISPQHASGSSTPVPSASGTMLSLSHEQILSQNYASNIKGKYRTNPRKGPSPRLSDTLMKQALSSVITQEKKRLKSQPKSIAQDIQPPNSMIKNALDEKDSHPFGDCFSDDEFLAIHLYTSCLYRPINHHLRYAPKNDVAPVVEAMKSGLAKLAQYPDYQVSGQLHRGIKQKMDDGEVMSRFKPGNTYRDDAFMSTSTRMDVTEEFTSDVTLHLQSSSAVNIGPFSKNPYEDEALIPPLTPFKVTSLHKQDDRWHVHLNEIAESSDE ``` |

---

### **C1E\_5023** 1087:79682..80245 Operon 59 Gene 1 gi|29171492|ref|NP\_808676.1| type III chaperone ShcO1

|  |  |  |  |
| --- | --- | --- | --- |
| 1e-107 | capillary\_sequence\_32 | ``` C1E_5023 1-187  capillary_sequence_32 136-696 ``` | ``` MLFLRKTEEATTILTPGSTQKVHVAWNLTEVHATKCSFEPKGIHTMRTSVNGLLEHSLKTLGFDTSALQALRDDGYLLWQGKDKQASLLVPSTDGDALFAICTLSRVDPEHDGRLLALALHLNLSPVHTMSACIALDVEQNTLCLRYTHDLGGNGADTLLLALENAQALAEQIKQVIENFRHDQGRR MLFLRKTEEATTILTPGSTQKVHVAWNLTEVHATKCSFEPKGIHTMRTSVNGLLEHSLKTLGFDTSALQALRDDGYLLWQGKDKQASLLVPSTDGDALFAICTLSRVDPEHDGRLLALALHLNLSPVHTMSACIALDVEQNTLCLRYTHDLGGNGADTLLLALENAQALAEQIKQVIENFRHDQGRR MLFLRKTEEATTILTPGSTQKVHVAWNLTEVHATKCSFEPKGIHTMRTSVNGLLEHSLKTLGFDTSALQALRDDGYLLWQGKDKQASLLVPSTDGDALFAICTLSRVDPEHDGRLLALALHLNLSPVHTMSACIALDVEQNTLCLRYTHDLGGNGADTLLLALENAQALAEQIKQVIENFRHDQGRR ``` |

---

### **C1E\_5300** 1087:315085..316227 Operon 198 Gene 1 gi|30231118|gb|AAP23130.1|AF461560\_2 AvrPphE

|  |  |  |  |
| --- | --- | --- | --- |
| 1e-148 | capillary\_sequence\_14 | ``` C1E_5300 118-378  capillary_sequence_14 2-784 ``` | ```                                                                                                                      GRGNIDVDAQRTHLQSGARAVAAKRLRKDAERAGHEPMPGNDEMNWHVLVAMSGQVFGAGNCGEHARIASFAYGALAQESGRSPREKIHLAEQPGKDHVWAETDNSSAGSSPIVMDPWSNGAAILAEDSRFAKDRSAVERTYSFTLAMAAEAGKVTRETAENVLTHTTSRLQKRLADQLPNVSPLEGGRYQQEKSVLDEAFARRVSDKLNSDDPRRALQMEIEAVGVAMSLGAEGVKTVARQAPKVVRQARSVASSKGMPP                                                                                                                      GRGNIDVDAQRTHLQSGARAVAAKRLRKDAERAGHEPMPGNDEMNWHVLVAMSGQVFGAGNCGEHARIASFAYGALAQESGRSPREKIHLAEQPGKDHVWAETDNSSAGSSPIVMDPWSNGAAILAEDSRFAKDRSAVERTYSFTLAMAAEAGKVTRETAENVLTHTTSRLQKRLADQLPNVSPLEGGRYQQEKSVLDEAFARRVSDKLNSDDPRRALQMEIEAVGVAMSLGAEGVKTVARQAPKVVRQARSVASSKGMPP                                                                                                                      GRGNIDVDAQRTHLQSGARAVAAKRLRKDAERAGHEPMPGNDEMNWHVLVAMSGQVFGAGNCGEHARIASFAYGALAQESGRSPREKIHLAEQPGKDHVWAETDNSSAGSSPIVMDPWSNGAAILAEDSRFAKDRSAVERTYSFTLAMAAEAGKVTRETAENVLTHTTSRLQKRLADQLPNVSPLEGGRYQQEKSVLDEAFARRVSDKLNSDDPRRALQMEIEAVGVAMSLGAEGVKTVARQAPKVVRQARSVASSKGMPP ``` |
| 1e-125 | capillary\_sequence\_33 | ``` C1E_5300 1-217  capillary_sequence_33 2618-3268 ``` | ``` MRIHSAGHSLPAPGPSVETTEKAVQSSSAQNPASCSSQTERPEAGSTQVRPNYPYSSVKTRLPPVSSTGQAISDTPSSLPGYLLLRRLDRRPLDEDSIKALVPADEAVREARRALPFGRGNIDVDAQRTHLQSGARAVAAKRLRKDAERAGHEPMPGNDEMNWHVLVAMSGQVFGAGNCGEHARIASFAYGALAQESGRSPREKIHLAEQPGKDHVW MRIHSAGHSLPAPGPSVETTEKAVQSSSAQNPASCSSQTERPEAGSTQVRPNYPYSSVKTRLPPVSSTGQAISDTPSSLPGYLLLRRLDRRPLDEDSIKALVPADEAVREARRALPFGRGNIDVDAQRTHLQSGARAVAAKRLRKDAERAGHEPMPGNDEMNWHVLVAMSGQVFGAGNCGEHARIASFAYGALAQESGRSPREKIHLAEQPGKDHVW MRIHSAGHSLPAPGPSVETTEKAVQSSSAQNPASCSSQTERPEAGSTQVRPNYPYSSVKTRLPPVSSTGQAISDTPSSLPGYLLLRRLDRRPLDEDSIKALVPADEAVREARRALPFGRGNIDVDAQRTHLQSGARAVAAKRLRKDAERAGHEPMPGNDEMNWHVLVAMSGQVFGAGNCGEHARIASFAYGALAQESGRSPREKIHLAEQPGKDHVW ``` |
| 1e-119 | capillary\_sequence\_51 | ``` C1E_5300 167-380  capillary_sequence_51 176-817 ``` | ```                                                                                                                                                                       VAMSGQVFGAGNCGEHARIASFAYGALAQESGRSPREKIHLAEQPGKDHVWAETDNSSAGSSPIVMDPWSNGAAILAEDSRFAKDRSAVERTYSFTLAMAAEAGKVTRETAENVLTHTTSRLQKRLADQLPNVSPLEGGRYQQEKSVLDEAFARRVSDKLNSDDPRRALQMEIEAVGVAMSLGAEGVKTVARQAPKVVRQARSVASSKGMPPRR                                                                                                                                                                       VAMSGQVFGAGNCGEHARIASFAYGALAQESGRSPREKIHLAEQPGKDHVWAETDNSSAGSSPIVMDPWSNGAAILAEDSRFAKDRSAVERTYSFTLAMAAEAGKVTRETAENVLTHTTSRLQKRLADQLPNVSPLEGGRYQQEKSVLDEAFARRVSDKLNSDDPRRALQMEIEAVGVAMSLGAEGVKTVARQAPKVVRQARSVASSKGMPPRR                                                                                                                                                                       VAMSGQVFGAGNCGEHARIASFAYGALAQESGRSPREKIHLAEQPGKDHVWAETDNSSAGSSPIVMDPWSNGAAILAEDSRFAKDRSAVERTYSFTLAMAAEAGKVTRETAENVLTHTTSRLQKRLADQLPNVSPLEGGRYQQEKSVLDEAFARRVSDKLNSDDPRRALQMEIEAVGVAMSLGAEGVKTVARQAPKVVRQARSVASSKGMPPRR ``` |

---

### **C1E\_5301** 1087:316323..318641 Operon 198 Gene 2 gi|71733886|ref|YP\_273554.1| type III effector HrpK1

|  |  |  |  |
| --- | --- | --- | --- |
| 0.0 | capillary\_sequence\_33 | ``` C1E_5301 1-772  capillary_sequence_33 204-2519 ``` | ``` MATMRISSSPSPALGSIVNQPTSGELAAETPLAKASLTQSSAGGDQAFVQFGQANDNTSFFSDAEQSGSSLMSLLTRSSNSESTSSVDQDSDQVSPMTSVLSTASASPAASASGPANAPSATDAAFLDNSEYSSPEALKRWDPMVAHLPPEEREQAAKELNRPIAAAWMAREHGPNADKAMAFINANPALKTAVDVGKDGGNADGKITNKDLKAFAKNMEKAADNADKDLAKYMEDNPGADPQSLEMVRSAAVMRANMPLATAADPHHAVGAPDKTDVDGNVSAEGLKALIKSNPGLSGTLKQSSNMWSQAGFLSQVDEAGLTGRKKAAHSPDQVFDASNMSEWIRKSAPKNGGQFASMLSDAATLNSVSGIDISKLNAQVFEKPKAYTGAQKAAVMIKLQQTQQSVIAGRDLRNTEKTEAGLNERIAQLQADPDVQEYLNKSIPEQERSLVRSDSALQKAVTEQAQNVNSGKALQTDLATADKAVDKHNPNPDYSGAITGLSAQLQLQKDLFPDAQVPTAQQVFNNQPDEVQTKIADSYVRNFSEGGALKQLLGQKKSDAGESLQTADNQKAAYESVLPADFVNGERESYTASTLSELQNSKKGRKLLEGKTDEEGGPSLAAQLAEQGIGGKAFNSVMGFASVSDRLASGDKLGAAQSIIDSSRLGAEAIKGGIDTGAKMMGREASAGLGRLGGQMIGRAVGLVAGEATGLAAGAALGAAIPVIGWAIDGAMALGFGISAIIDAVKKHKAQKAFDHNVDPVLDQFGIAKAH MATMRISSSPSPALGSIVNQPTSGELAAETPLAKASLTQSSAGGDQAFVQFGQANDNTSFFSDAEQSGSSLMSLLTRSSNSESTSSVDQDSDQVSPMTSVLSTASASPAASASGPANAPSATDAAFLDNSEYSSPEALKRWDPMVAHLPPEEREQAAKELNRPIAAAWMAREHGPNADKAMAFINANPALKTAVDVGKDGGNADGKITNKDLKAFAKNMEKAADNADKDLAKYMEDNPGADPQSLEMVRSAAVMRANMPLATAADPHHAVGAPDKTDVDGNVSAEGLKALIKSNPGLSGTLKQSSNMWSQAGFLSQVDEAGLTGRKKAAHSPDQVFDASNMSEWIRKSAPKNGGQFASMLSDAATLNSVSGIDISKLNAQVFEKPKAYTGAQKAAVMIKLQQTQQSVIAGRDLRNTEKTEAGLNERIAQLQADPDVQEYLNKSIPEQERSLVRSDSALQKAVTEQAQNVNSGKALQTDLATADKAVDKHNPNPDYSGAITGLSAQLQLQKDLFPDAQVPTAQQVFNNQPDEVQTKIADSYVRNFSEGGALKQLLGQKKSDAGESLQTADNQKAAYESVLPADFVNGERESYTASTLSELQNSKKGRKLLEGKTDEEGGPSLAAQLAEQGIGGKAFNSVMGFASVSDRLASGDKLGAAQSIIDSSRLGAEAIKGGIDTGAKMMGREASAGLGRLGGQMIGRAVGLVAGEATGLAAGAALGAAIPVIGWAIDGAMALGFGISAIIDAVKKHKAQKAFDHNVDPVLDQFGIAKAH MATMRISSSPSPALGSIVNQPTSGELAAETPLAKASLTQSSAGGDQAFVQFGQANDNTSFFSDAEQSGSSLMSLLTRSSNSESTSSVDQDSDQVSPMTSVLSTASASPAASASGPANAPSATDAAFLDNSEYSSPEALKRWDPMVAHLPPEEREQAAKELNRPIAAAWMAREHGPNADKAMAFINANPALKTAVDVGKDGGNADGKITNKDLKAFAKNMEKAADNADKDLAKYMEDNPGADPQSLEMVRSAAVMRANMPLATAADPHHAVGAPDKTDVDGNVSAEGLKALIKSNPGLSGTLKQSSNMWSQAGFLSQVDEAGLTGRKKAAHSPDQVFDASNMSEWIRKSAPKNGGQFASMLSDAATLNSVSGIDISKLNAQVFEKPKAYTGAQKAAVMIKLQQTQQSVIAGRDLRNTEKTEAGLNERIAQLQADPDVQEYLNKSIPEQERSLVRSDSALQKAVTEQAQNVNSGKALQTDLATADKAVDKHNPNPDYSGAITGLSAQLQLQKDLFPDAQVPTAQQVFNNQPDEVQTKIADSYVRNFSEGGALKQLLGQKKSDAGESLQTADNQKAAYESVLPADFVNGERESYTASTLSELQNSKKGRKLLEGKTDEEGGPSLAAQLAEQGIGGKAFNSVMGFASVSDRLASGDKLGAAQSIIDSSRLGAEAIKGGIDTGAKMMGREASAGLGRLGGQMIGRAVGLVAGEATGLAAGAALGAAIPVIGWAIDGAMALGFGISAIIDAVKKHKAQKAFDHNVDPVLDQFGIAKAH ``` |
| 1e-110 | capillary\_sequence\_15 | ``` C1E_5301 1-200  capillary_sequence_15 126-725 ``` | ``` MATMRISSSPSPALGSIVNQPTSGELAAETPLAKASLTQSSAGGDQAFVQFGQANDNTSFFSDAEQSGSSLMSLLTRSSNSESTSSVDQDSDQVSPMTSVLSTASASPAASASGPANAPSATDAAFLDNSEYSSPEALKRWDPMVAHLPPEEREQAAKELNRPIAAAWMAREHGPNADKAMAFINANPALKTAVDVGKDG MATMRISSSPSPALGSIVNQPTSGELAAETPLAKASLTQSSAGGDQAFVQFGQANDNTSFFSDAEQSGSSLMSLLTRSSNSESTSSVDQDSDQVSPMTSVLSTASASPAASASGPANAPSATDAAFLDNSEYSSPEALKRWDPMVAHLPPEEREQAAKELNRPIAAAWMAREHGPNADKAMAFINANPALKTAVDVGKDG MATMRISSSPSPALGSIVNQPTSGELAAETPLAKASLTQSSAGGDQAFVQFGQANDNTSFFSDAEQSGSSLMSLLTRSSNSESTSSVDQDSDQVSPMTSVLSTASASPAASASGPANAPSATDAAFLDNSEYSSPEALKRWDPMVAHLPPEEREQAAKELNRPIAAAWMAREHGPNADKAMAFINANPALKTAVDVGKDG ``` |
| 1e-105 | capillary\_sequence\_50 | ``` C1E_5301 1-179  capillary_sequence_50 271-807 ``` | ``` MATMRISSSPSPALGSIVNQPTSGELAAETPLAKASLTQSSAGGDQAFVQFGQANDNTSFFSDAEQSGSSLMSLLTRSSNSESTSSVDQDSDQVSPMTSVLSTASASPAASASGPANAPSATDAAFLDNSEYSSPEALKRWDPMVAHLPPEEREQAAKELNRPIAAAWMAREHGPNADK MATMRISSSPSPALGSIVNQPTSGELAAETPLAKASLTQSSAGGDQAFVQFGQANDNTSFFSDAEQSGSSLMSLLTRSSNSESTSSVDQDSDQVSPMTSVLSTASASPAASASGPANAPSATDAAFLDNSEYSSPEALKRWDPMVAHLPPEEREQAAKELNRPIAAAWMAREHGPNAD+ MATMRISSSPSPALGSIVNQPTSGELAAETPLAKASLTQSSAGGDQAFVQFGQANDNTSFFSDAEQSGSSLMSLLTRSSNSESTSSVDQDSDQVSPMTSVLSTASASPAASASGPANAPSATDAAFLDNSEYSSPEALKRWDPMVAHLPPEEREQAAKELNRPIAAAWMAREHGPNADR ``` |
| 1e-105 | capillary\_sequence\_50 | ``` C1E_5301 175-200  capillary_sequence_50 792-869 ``` | ```                                                                                                                                                                               PNADKAMAFINANPALKTAVDVGKDG                                                                                                                                                                               P   +AMAFINANPALKTAVDVGKDG                                                                                                                                                                               PQC*QAMAFINANPALKTAVDVGKDG ``` |

---

### **C1E\_5342** 1087:352578..353465 Operon 211 Gene 1 gi|28868580|ref|NP\_791199.1| type III effector HopAA1-1

|  |  |  |  |
| --- | --- | --- | --- |
| 1e-158 | capillary\_sequence\_25 | ``` C1E_5342 11-295  capillary_sequence_25 920-1774 ``` | ```           GLGRQVVDMGIAVQTYSARNAVRTVLAPALASRPAVQSAVDISVSTAGGLAANAGFGNRMLSVQSRDHLRGGAFVLGLKDKEPKADLNEETDWLDAYNAIKSASYSGAALNAGKRMAGLPLDIATDGLKAVRSLVSATSLVQNGVALAGGFAGVGKLQEMATKNITHPATSAAVSQLTNLAGSAGVFAGWTTAALATDPAVKKAESFIQDTVKSTASSTTGYVADQTVKLAKTVKDKGGEALVNTGASLRNTVNNLRHRPAREADIEEGGIAASPSETPFQPGWS           GL  QVVDMGIAVQTYSARNAVRTVLAPALASRPAVQSAVDISVSTAGGLAANAGFGNRMLSVQSRDHLRGGAFVLGLKDKEPKADLNEETDWLDAYNAIKSASYSGAALNAGKRMAGLPLDIATDGLKAVRSLVSATSLVQNGVALAGGFAGVGKLQEMATKNITHPATSAAVSQLTNLAGSAGVFAGWTTAALATDPAVKKAESFIQDTVKSTASSTTGYVADQTVKLAKTVKDKGGEALVNTGASLRNTVNNLRHRPAREADIEEGGIAASPSETPFQPGWS           GLAGQVVDMGIAVQTYSARNAVRTVLAPALASRPAVQSAVDISVSTAGGLAANAGFGNRMLSVQSRDHLRGGAFVLGLKDKEPKADLNEETDWLDAYNAIKSASYSGAALNAGKRMAGLPLDIATDGLKAVRSLVSATSLVQNGVALAGGFAGVGKLQEMATKNITHPATSAAVSQLTNLAGSAGVFAGWTTAALATDPAVKKAESFIQDTVKSTASSTTGYVADQTVKLAKTVKDKGGEALVNTGASLRNTVNNLRHRPAREADIEEGGIAASPSETPFQPGWS ``` |
| 1e-158 | capillary\_sequence\_25 | ``` C1E_5342 6-14  capillary_sequence_25 904-930 ``` | ```      KRHSPGLGR      KRHSPGLGR      KRHSPGLGR ``` |
| 1e-156 | capillary\_sequence\_60 | ``` C1E_5342 1-283  capillary_sequence_60 1-849 ``` | ``` MAASVKRHSPGLGRQVVDMGIAVQTYSARNAVRTVLAPALASRPAVQSAVDISVSTAGGLAANAGFGNRMLSVQSRDHLRGGAFVLGLKDKEPKADLNEETDWLDAYNAIKSASYSGAALNAGKRMAGLPLDIATDGLKAVRSLVSATSLVQNGVALAGGFAGVGKLQEMATKNITHPATSAAVSQLTNLAGSAGVFAGWTTAALATDPAVKKAESFIQDTVKSTASSTTGYVADQTVKLAKTVKDKGGEALVNTGASLRNTVNNLRHRPAREADIEEGGIAA MAASVKRHSPGLGRQVVDMGIAVQTYSARNAVRTVLAPALASRPAVQSAVDISVSTAGGLAANAGFGNRMLSVQSRDHLRGGAFVLGLKDKEPKADLNEETDWLDAYNAIKSASYSGAALNAGKRMAGLPLDIATDGLKAVRSLVSATSLVQNGVALAGGFAGVGKLQEMATKNITHPATSAAVSQLTNLAGSAGVFAGWTTAALATDPAVKKAESFIQDTVKSTASSTTGYVADQTVKLAKTVKDKGGEALVNTGASLRNTVNNLRHRPAREADIEEGGIAA MAASVKRHSPGLGRQVVDMGIAVQTYSARNAVRTVLAPALASRPAVQSAVDISVSTAGGLAANAGFGNRMLSVQSRDHLRGGAFVLGLKDKEPKADLNEETDWLDAYNAIKSASYSGAALNAGKRMAGLPLDIATDGLKAVRSLVSATSLVQNGVALAGGFAGVGKLQEMATKNITHPATSAAVSQLTNLAGSAGVFAGWTTAALATDPAVKKAESFIQDTVKSTASSTTGYVADQTVKLAKTVKDKGGEALVNTGASLRNTVNNLRHRPAREADIEEGGIAA ``` |
| 1e-149 | capillary\_sequence\_62 | ``` C1E_5342 11-283  capillary_sequence_62 256-1074 ``` | ```           GLGRQVVDMGIAVQTYSARNAVRTVLAPALASRPAVQSAVDISVSTAGGLAANAGFGNRMLSVQSRDHLRGGAFVLGLKDKEPKADLNEETDWLDAYNAIKSASYSGAALNAGKRMAGLPLDIATDGLKAVRSLVSATSLVQNGVALAGGFAGVGKLQEMATKNITHPATSAAVSQLTNLAGSAGVFAGWTTAALATDPAVKKAESFIQDTVKSTASSTTGYVADQTVKLAKTVKDKGGEALVNTGASLRNTVNNLRHRPAREADIEEGGIAA           GL  QVVDMGIAVQTYSARNAVRTVLAPALASRPAVQSAVDISVSTAGGLAANAGFGNRMLSVQSRDHLRGGAFVLGLKDKEPKADLNEETDWLDAYNAIKSASYSGAALNAGKRMAGLPLDIATDGLKAVRSLVSATSLVQNGVALAGGFAGVGKLQEMATKNITHPATSAAVSQLTNLAGSAGVFAGWTTAALATDPAVKKAESFIQDTVKSTASSTTGYVADQTVKLAKTVKDKGGEALVNTGASLRNTVNNLRHRPAREADIEEGGIAA           GLAGQVVDMGIAVQTYSARNAVRTVLAPALASRPAVQSAVDISVSTAGGLAANAGFGNRMLSVQSRDHLRGGAFVLGLKDKEPKADLNEETDWLDAYNAIKSASYSGAALNAGKRMAGLPLDIATDGLKAVRSLVSATSLVQNGVALAGGFAGVGKLQEMATKNITHPATSAAVSQLTNLAGSAGVFAGWTTAALATDPAVKKAESFIQDTVKSTASSTTGYVADQTVKLAKTVKDKGGEALVNTGASLRNTVNNLRHRPAREADIEEGGIAA ``` |
| 1e-149 | capillary\_sequence\_2 | ``` C1E_5342 11-283  capillary_sequence_2 1-819 ``` | ```           GLGRQVVDMGIAVQTYSARNAVRTVLAPALASRPAVQSAVDISVSTAGGLAANAGFGNRMLSVQSRDHLRGGAFVLGLKDKEPKADLNEETDWLDAYNAIKSASYSGAALNAGKRMAGLPLDIATDGLKAVRSLVSATSLVQNGVALAGGFAGVGKLQEMATKNITHPATSAAVSQLTNLAGSAGVFAGWTTAALATDPAVKKAESFIQDTVKSTASSTTGYVADQTVKLAKTVKDKGGEALVNTGASLRNTVNNLRHRPAREADIEEGGIAA           GL  QVVDMGIAVQTYSARNAVRTVLAPALASRPAVQSAVDISVSTAGGLAANAGFGNRMLSVQSRDHLRGGAFVLGLKDKEPKADLNEETDWLDAYNAIKSASYSGAALNAGKRMAGLPLDIATDGLKAVRSLVSATSLVQNGVALAGGFAGVGKLQEMATKNITHPATSAAVSQLTNLAGSAGVFAGWTTAALATDPAVKKAESFIQDTVKSTASSTTGYVADQTVKLAKTVKDKGGEALVNTGASLRNTVNNLRHRPAREADIEEGGIAA           GLAGQVVDMGIAVQTYSARNAVRTVLAPALASRPAVQSAVDISVSTAGGLAANAGFGNRMLSVQSRDHLRGGAFVLGLKDKEPKADLNEETDWLDAYNAIKSASYSGAALNAGKRMAGLPLDIATDGLKAVRSLVSATSLVQNGVALAGGFAGVGKLQEMATKNITHPATSAAVSQLTNLAGSAGVFAGWTTAALATDPAVKKAESFIQDTVKSTASSTTGYVADQTVKLAKTVKDKGGEALVNTGASLRNTVNNLRHRPAREADIEEGGIAA ``` |

---

### **C1E\_5343** 1087:353470..354039 Operon 211 Gene 2 gi|28871842|ref|NP\_794461.1| type III effector HopAA1-2

|  |  |  |  |
| --- | --- | --- | --- |
| 1e-107 | capillary\_sequence\_63 | ``` C1E_5343 1-189  capillary_sequence_63 288-854 ``` | ``` MHINRSAPQPPGIEMESFRTESDASLASSSVRSVSSVSQGNLQAITDYLKDYVFAAHKLPLTDSLDDHAAIYAHNEQIDALIDARARRLSDQGETPLSIGETFAKAEKFDRLATTASSALRATPFAAASVLQYMQPGINKGDWLPAPLKPLTPFISGALSGAMDQVGTKMMDRATGDLHYLSTSPDRHP MHINRSAPQPPGIEMESFRTESDASLASSSVRSVSSVSQGNLQAITDYLKDYVFAAHKLPLTDSLDDHAAIYAHNEQIDALIDARARRLSDQGETPLSIGETFAKAEKFDRLATTASSALRATPFAAASVLQYMQPGINKGDWLPAPLKPLTPFISGALSGAMDQVGTKMMDRATGDLHYLSTSPDRHP MHINRSAPQPPGIEMESFRTESDASLASSSVRSVSSVSQGNLQAITDYLKDYVFAAHKLPLTDSLDDHAAIYAHNEQIDALIDARARRLSDQGETPLSIGETFAKAEKFDRLATTASSALRATPFAAASVLQYMQPGINKGDWLPAPLKPLTPFISGALSGAMDQVGTKMMDRATGDLHYLSTSPDRHP ``` |
| 1e-107 | capillary\_sequence\_61 | ``` C1E_5343 1-189  capillary_sequence_61 294-860 ``` | ``` MHINRSAPQPPGIEMESFRTESDASLASSSVRSVSSVSQGNLQAITDYLKDYVFAAHKLPLTDSLDDHAAIYAHNEQIDALIDARARRLSDQGETPLSIGETFAKAEKFDRLATTASSALRATPFAAASVLQYMQPGINKGDWLPAPLKPLTPFISGALSGAMDQVGTKMMDRATGDLHYLSTSPDRHP MHINRSAPQPPGIEMESFRTESDASLASSSVRSVSSVSQGNLQAITDYLKDYVFAAHKLPLTDSLDDHAAIYAHNEQIDALIDARARRLSDQGETPLSIGETFAKAEKFDRLATTASSALRATPFAAASVLQYMQPGINKGDWLPAPLKPLTPFISGALSGAMDQVGTKMMDRATGDLHYLSTSPDRHP MHINRSAPQPPGIEMESFRTESDASLASSSVRSVSSVSQGNLQAITDYLKDYVFAAHKLPLTDSLDDHAAIYAHNEQIDALIDARARRLSDQGETPLSIGETFAKAEKFDRLATTASSALRATPFAAASVLQYMQPGINKGDWLPAPLKPLTPFISGALSGAMDQVGTKMMDRATGDLHYLSTSPDRHP ``` |
| 1e-107 | capillary\_sequence\_25 | ``` C1E_5343 1-189  capillary_sequence_25 314-880 ``` | ``` MHINRSAPQPPGIEMESFRTESDASLASSSVRSVSSVSQGNLQAITDYLKDYVFAAHKLPLTDSLDDHAAIYAHNEQIDALIDARARRLSDQGETPLSIGETFAKAEKFDRLATTASSALRATPFAAASVLQYMQPGINKGDWLPAPLKPLTPFISGALSGAMDQVGTKMMDRATGDLHYLSTSPDRHP MHINRSAPQPPGIEMESFRTESDASLASSSVRSVSSVSQGNLQAITDYLKDYVFAAHKLPLTDSLDDHAAIYAHNEQIDALIDARARRLSDQGETPLSIGETFAKAEKFDRLATTASSALRATPFAAASVLQYMQPGINKGDWLPAPLKPLTPFISGALSGAMDQVGTKMMDRATGDLHYLSTSPDRHP MHINRSAPQPPGIEMESFRTESDASLASSSVRSVSSVSQGNLQAITDYLKDYVFAAHKLPLTDSLDDHAAIYAHNEQIDALIDARARRLSDQGETPLSIGETFAKAEKFDRLATTASSALRATPFAAASVLQYMQPGINKGDWLPAPLKPLTPFISGALSGAMDQVGTKMMDRATGDLHYLSTSPDRHP ``` |
| 1e-107 | capillary\_sequence\_3 | ``` C1E_5343 1-189  capillary_sequence_3 288-854 ``` | ``` MHINRSAPQPPGIEMESFRTESDASLASSSVRSVSSVSQGNLQAITDYLKDYVFAAHKLPLTDSLDDHAAIYAHNEQIDALIDARARRLSDQGETPLSIGETFAKAEKFDRLATTASSALRATPFAAASVLQYMQPGINKGDWLPAPLKPLTPFISGALSGAMDQVGTKMMDRATGDLHYLSTSPDRHP MHINRSAPQPPGIEMESFRTESDASLASSSVRSVSSVSQGNLQAITDYLKDYVFAAHKLPLTDSLDDHAAIYAHNEQIDALIDARARRLSDQGETPLSIGETFAKAEKFDRLATTASSALRATPFAAASVLQYMQPGINKGDWLPAPLKPLTPFISGALSGAMDQVGTKMMDRATGDLHYLSTSPDRHP MHINRSAPQPPGIEMESFRTESDASLASSSVRSVSSVSQGNLQAITDYLKDYVFAAHKLPLTDSLDDHAAIYAHNEQIDALIDARARRLSDQGETPLSIGETFAKAEKFDRLATTASSALRATPFAAASVLQYMQPGINKGDWLPAPLKPLTPFISGALSGAMDQVGTKMMDRATGDLHYLSTSPDRHP ``` |
